# An RNA-aptamer-based two-color CRISPR labeling system

**DOI:** 10.1038/srep26857

**Published:** 2016-05-27

**Authors:** Siyuan Wang, Jun-Han Su, Feng Zhang, Xiaowei Zhuang

**Affiliations:** 1 Howard Hughes Medical Institute, Cambridge, MA 02138, USA; 2 Department of Chemistry and Chemical Biology and Department of Physics, Harvard University, Cambridge, MA 02138, USA; 3 Broad Institute of MIT and Harvard, Cambridge, MA 02142, USA; 4 McGovern Institute for Brain Research, MIT, Cambridge, MA 02139, USA; 5 Department of Brain and Cognitive Sciences and Department of Biological Engineering, MIT, Cambridge, MA 02139, USA.

## Abstract

The spatial organization and dynamics of chromatin play important roles in essential biological functions. However, direct visualization of endogenous genomic loci in living cells has proven to be laborious until the recent development of CRISPR-Cas9-based chromatin labeling methods. These methods rely on the recognition of specific DNA sequences by CRISPR single-guide RNAs (sgRNAs) and fluorescent–protein-fused catalytically inactive Cas9 to label specific chromatin loci in cells. Previously, multicolor chromatin labeling has been achieved using orthogonal Cas9 proteins from different bacterial species fused to different fluorescent proteins. Here we report the development of an alternative two-color CRISPR labeling method using only the well-characterized *Streptococcus pyogenes* Cas9, by incorporating MS2 or PP7 RNA aptamers into the sgRNA. The MS2 or PP7 aptamers then recruit the corresponding MS2 or PP7 coat proteins fused with different fluorescent proteins to the target genomic loci. Here we demonstrate specific and orthogonal two-color labeling of repetitive sequences in living human cells using this method. By attaching the MS2 or PP7 aptamers to different locations on the sgRNA, we found that extending the tetraloop and stem loop 2 of the sgRNA with MS2 or PP7 aptamers enhances the signal-to-background ratio of chromatin imaging.

Dynamic changes in chromatin organization underlie many essential biological processes^1^,^2^,^3^. To study the intricate relationship between chromatin dynamics and biological functions, various methods have been developed to image specific genomic DNA sequences *in vivo*. Traditionally, locus-specific live cell DNA labeling has relied on fluorescent repressor operator systems (FROS)^4^, which introduce large arrays of operator repeats near a genomic locus of interest. The operator repeats can be subsequently visualized with fluorescently labeled repressor proteins that bind specifically to the operator sites. This method, however, perturbs the endogenous DNA sequence and requires repeated, time-consuming efforts to insert the operator arrays to all target loci. More recent approaches adopted programmable DNA-binding proteins from genome engineering studies, such as transcription activator-like effectors (TALEs) and clustered regularly interspersed short palindromic repeat (CRISPR) – associated protein 9 (Cas9), to directly label the endogenous DNA sequence^5^,^6^. In the latter approach, endogenous genomic loci are labeled using nuclease-deactivated Cas9 (dCas9) conjugated with a fluorescent protein and programed with sgRNAs targeting an array of sequences at each chromatin locus^6^. The programmable and scalable nature of this method should allow for easy adaptation to study the dynamics of various genomic loci in living cells. Recently, this method has been extended to achieve multicolor labeling of repetitive sequences in the human genome by leveraging orthogonal dCas9/sgRNA combinations from diverse bacterial species such as *Streptococcus pyogenes*, *Neisseria meningitides*, *Streptococcus thermophilus* and *Staphylococcus aureus*^7^,^8^. However, the *N. meningitides*, *S. thermophilus* and *S. aureus* CRISPR-Cas9 systems have more complex protospacer adjacent motifs (PAMs) than the *S. pyogenes* CRISPR-Cas9^9^,^10^, and are less well-characterized. Here we report an alternative two-color CRISPR labeling method based on only the well-characterized *S. pyogenes* CRISPR-Cas9 and the specific interactions of two orthogonal RNA aptamers MS2 and PP7 with their respective binding proteins, MS2 coat protein (MCP) and PP7 coat protein (PCP)^11^, a strategy that has been previously used to simultaneously recruit different functional molecules to distinct genomic loci in the same cell^12^,^13^.

## Results

**Figure 1:**
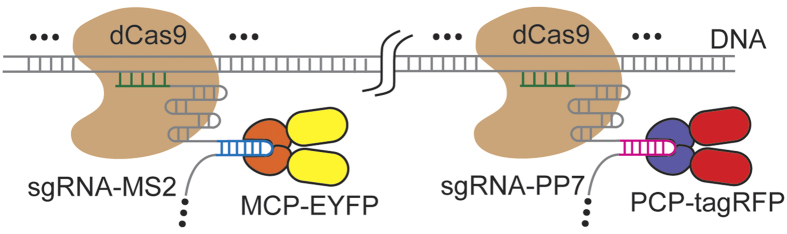
Schematic illustration of the RNA-aptamer-based two-color CRISPR labeling strategy. The DNA and sgRNAs are colored in grey. The spacer regions of the sgRNAs are colored in green. The MS2 and PP7 hairpins are colored in blue and magenta respectively. The dCas9, MCP, EYFP, PCP, and tagRFP proteins are colored in brown, orange, yellow, purple, and red, respectively. The “…” markers indicate more than one dCas9 complexes or MS2/PP7 hairpins.

In our labeling design, an array of dCas9 proteins programmed with sgRNAs are targeted to the genomic locus, and the sgRNAs are labeled by extending the sgRNA to include multiple MS2 or PP7 aptamer hairpins, which recruit MCP or PCP fused to fluorescent proteins (Fig. 1). This strategy should recruit fluorescent proteins to the specific genomic loci to enable labeling. The use of both MS2 and PP7, together with their corresponding coating proteins MCP and PCP fused to fluorescent proteins of different colors should enable multi-color chromatin labeling (Fig. 1).

**Figure 2:**
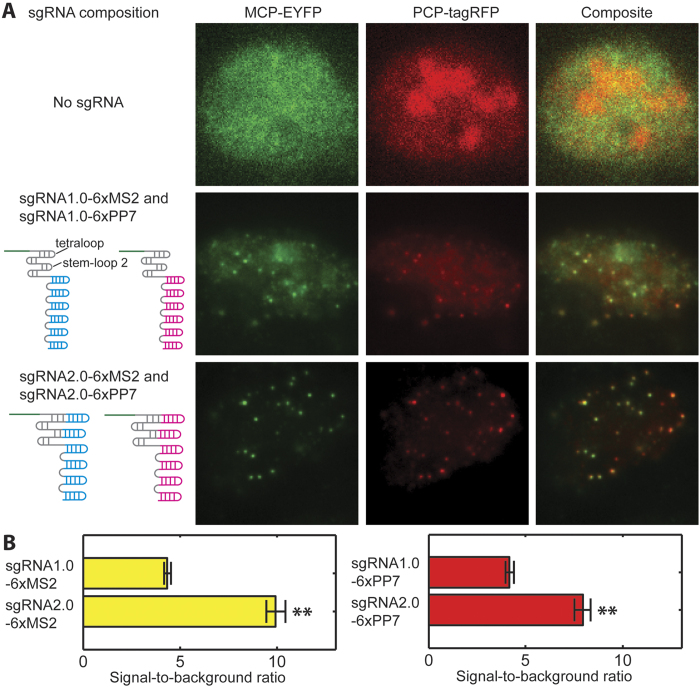
Specific two-color labeling of telomeres with the RNA-aptamer-based CRISPR labeling strategy. (**A**) Schematic illustration of the different sgRNA designs (left) and the corresponding sample images of labeled telomeres (right). First row: No sgRNA was introduced into the cells. Second row: sgRNA1.0-6xMS2 and sgRNA1.0-6xPP7 were co-expressed in the cells. Third row: sgRNA2.0-6xMS2 and sgRNA2.0-6xPP7 were co-expressed in the cells. (**B**) Signal-to-background ratios of the imaged telomere foci. Error bars represent standard errors of the means. N = 100 (100 foci were randomly selected from 10 cells for each analysis). **p < 0.001.

To implement this design, we first generated a U-2 OS cell line stably expressing *S. pyogenes* dCas9 fused to ECFP, MCP fused to EYFP, and PCP fused to tagRFP^14^. All three proteins were engineered to carry nuclear localization signals. The ECFP tag on dCas9 was used solely for the selection of dCas9-positive cells and was not used for imaging labeled genomic loci. The dCas9-ECFP expression was driven by a strong CMV promoter to promote efficient binding of dCas9 to sgRNAs. MCP-EYFP and PCP-tagRFP were expressed from a weak UbC promoter to ensure low background fluorescence in the nuclei. In the absence of sgRNA, MCP-EYFP had a diffuse nuclear localization pattern (Fig. 2A, first row). PCP-tagRFP showed a similar pattern, with more nucleolar enrichment (Fig. 2A, first row). Next, we engineered and compared two different sgRNA-aptamer compositions. In the first composition, termed the sgRNA1.0 design, six MS2 or PP7 hairpins were added to the 3′ end of the sgRNA (Fig. 2A, second row). We used six hairpins instead of one to promote efficient recruitment of the MCP-EYFP or PCP-tagRFP to each sgRNA molecule. In the second composition, termed the sgRNA2.0 design, two MS2 or PP7 hairpins were appended to the tetraloop and stem loop 2 in the middle of the sgRNA^15^, and four more MS2 or PP7 hairpins were added to the 3′ end (Fig. 2A, third row). As a proof of concept, both MS2 and PP7 versions of sgRNA were designed to target telomere repeats.

Cells co-transfected with plasmids expressing sgRNA1.0-6xMS2 and sgRNA1.0-6xPP7, or with plasmid expressing sgRNA2.0-6xMS2 and sgRNA2.0-6xPP7 both showed nuclear foci that resemble telomeres (Fig. 2A, second and third rows). Foci signals from the EYFP and tagRFP color channels coincided in space as expected, indicating telomeres were co-labeled by dCas9s programmed with MS2 and PP7 tagged sgRNAs. The varying ratios of the EYFP-to-tagRFP fluorescence intensity for different foci were largely caused by an intrinsic inhomogeneity in the laser illumination fields of our imaging setup. There could also be additional variance in the ratio of EYFP and tagRFP molecules recruited to each telomere. The high expression of dCas9-ECFP and the resulting strong ECFP fluorescence in the nuclei did not interfere with the identification of fluorescent foci in the EYFP and tagRFP channels (Fig. S1). On average, foci from cells expressing sgRNA2.0 showed higher signal-to-background ratio than those from cells expressing sgRNA1.0 (Fig. 2B).

**Figure 3:**
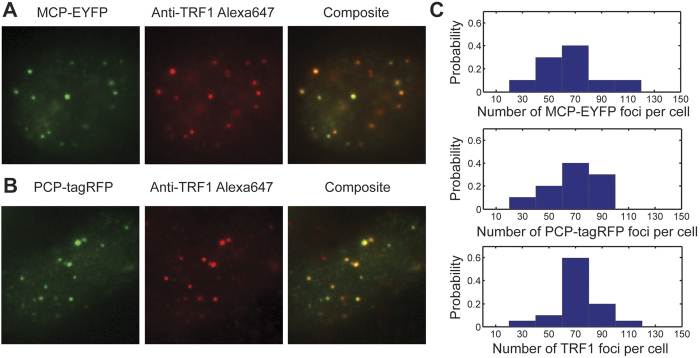
Specificity and efficiency of the RNA-aptamer-based CRISPR labeling strategy. (**A**) Cells transfected with sgRNA2.0-6xMS2 targeting telomeres, and immunostained with primary antibodies targeting telomeric protein TRF1 and secondary antibodies labeled with Alexa 647 dye. (**B**) Cells transfected with sgRNA2.0-6xPP7 targeting telomeres, and immunostained with the same antibodies as in (**A**). (**C**) Numbers of telomeres per cell detected in the EYFP, tagRFP and Alexa 647 channels. N = 10 cells for the EYFP and tagRFP channels, and 20 cells for the Alexa 647 channel.

To confirm that the fluorescently-labeled foci indeed represent telomere labeling, we expressed either sgRNA2.0-6xMS2 or sgRNA2.0-6xPP7 in cells, and performed immunofluorescence staining with primary antibodies targeting the telomeric protein TRF1 and Alexa 647 dye labeled secondary antibodies. Foci in the Alexa 647 channel coincided with those in the EYFP and tagRFP channels, indicating that the EYFP and tagRFP foci indeed corresponded to telomeres (Fig. 3A,B). Next, we counted the numbers of EYFP, tagRFP, and Alexa 647 foci per cell in these samples and found that the three numbers are similar, indicating comparable labeling efficiencies among the MS2- and PP7-based CRISPR labeling and the immunostaining (Fig. 3C). The number of observed telomere foci per cell was consistent with a previous report using peptide nucleic acid probes targeting telomeres in U-2 OS cells^16^.

**Figure 4:**
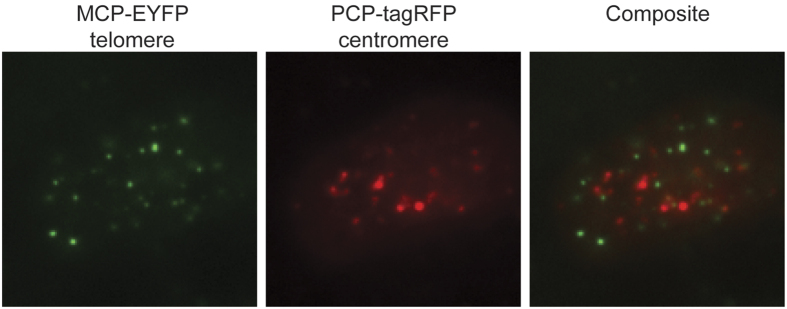
Orthogonality of the RNA-aptamer-based CRISPR labeling strategy. Cells co-transfected with sgRNA2.0-6xMS2 targeting telomeres and sgRNA2.0-6xPP7 targeting centromeres were imaged.

To demonstrate the orthogonality of the two RNA aptamers, we designed a new sgRNA2.0-6xPP7 targeting centromeric repeats, and expressed it in cells together with the sgRNA2.0-6xMS2 targeting telomeres. The cells showed largely non-overlapping EYFP and tagRFP foci in the nuclei (Fig. 4), indicating that the MS2- and PP7-based CRISPR labeling are orthogonal. In contrast, cells co-expressing sgRNA2.0-6xMS2 and sgRNA2.0-6xPP7 constructs both targeting centromeres showed coinciding foci as expected (Fig. S1B).

## Discussion

We have presented here a two-color CRISPR-Cas9 labeling system and demonstrated its ability to enable visualization of endogenous genomic loci in human cells. This method relies on extending sgRNAs with orthogonal RNA aptamers that can be bound by aptamer-coat proteins tagged with different fluorescent proteins. Interestingly, the sgRNA2.0 design supported higher contrast labeling than the sgRNA1.0 design, despite having the same number of aptmer hairpins. A previous study on aptamer-mediated gene activation compared an sgRNA with two MS2 hairpins at the 3′ end and an sgRNA with two MS2 hairpins appended to the tetraloop and stem loop 2, and found the latter design led to higher levels of transcription activation when co-expressed with dCas9 and MCP fused to VP64 transactivator domain^15^. Together with this previous observation, our results suggest that the extension of the tetraloop and stem loop 2 with RNA aptamers and the binding of coat proteins to the extension may help stabilize the dCas9-sgRNA interaction, which in turn helps with the binding of the dCas9-sgRNA complex to DNA.

Although our proof-of-concept demonstration has been on repetitive sequences, one could potentially expand the labeling capacity of this method to non-repetitive loci in the genome by simultaneously expressing sets of sgRNA species targeting adjacent sequences around each locus of interest, as was shown before for single-color CRISPR labeling^6^. Because the *S. pyogenes* CRIPSR-Cas9 system has the simplest PAM sequence among all the CRIPSR-Cas9 systems adopted for DNA labeling^9^,^10^, one potential advantage of our method over previous two-color CRIPSR labeling strategies^7^,^8^ is that our method may allow easier selection of targeting sequences around certain genomic loci of interest. One could also potentially add more colors by incorporating more orthogonal RNA aptamers, such as the com system^12^. While our paper was under review, another group reported similar modifications to sgRNA being effective in DNA labeling^17^, which further supports the promise of our approach. One disadvantage of our design is that it involves one more protein construct than previous methods^7^,^8^ (e.g. for two-color labeling, our design includes three fusion proteins while the previous methods only need two), which leads to a slightly more complex cell-line construction procedure. Further experiments are needed to test the full potential of the RNA-aptamer-based CRISPR labeling system.

## Methods

### Plasmids

A list of the new plasmids in this work is provided in Table 1. All the plasmids were cloned with PCR and isothermal assembly^18^. To generate pLVX-MCP-EYFP, the *UbC-mcp::eyfp* sequence was amplified from plasmid UbC NLS-HA-MCP-YFP^19^ (Addgene Plasmid #31230), and cloned into a Clontech pLVX-TRE3G backbone, replacing the original *TRE3GV* promoter. To generate pLVX- PCP-tagRFP, the *mcp* and *eyfp* sequences in the plasmid above were replaced with *pcp* and *tagRFP*, amplified from plasmids phage-ubc-nls-ha-tdPCP-gfp^20^ (Addgene Plasmid #40650) and pTagRFP-laminB1 (Evrogen) respectively. To introduce dCas9 into cells and quickly screen for high-expression clones with a cell sorter (described in the following sub-section), we generated a plasmid carrying a dCas9 fusion with enhanced cyan fluorescent protein (ECFP): pLVX-dCas9-ECFP. Chemically synthesized *Streptococcus pyogenes cas9* gene carrying the D10A and H840A mutations and flanked by nuclear localization signals (Genewiz) was fused to the *CMV* promoter and *ecfp* sequences amplified from a Clontech pECFP-C1 plasmid, and cloned into the pLVX-TRE3G backbone, replacing the original *TRE3GV* promoter. Next, the original puromycin resistance gene in the plasmid was replaced by a zeocin resistance gene, amplified from pBABE-zeo^21^ (Addgene Plasmid #1766). To generate plasmids carrying different sgRNA constructs, including psgRNA1.0-6xMS2-telo, psgRNA1.0-6xPP7-telo, psgRNA2.0-6xMS2-telo, psgRNA2.0-6xPP7-telo, psgRNA2.0-6xMS2-cent and psgRNA2.0-6xPP7-cent, chemically synthesized sgRNA constructs (Integrated DNA Technologies) were fused to the *U6* promoter amplified from pX260^22^ (Addgene Plasmid #42229), and cloned into a Clontech pEYFP-N1 backbone, replacing the original *CMV* promoter and *eyfp* sequences. The sequence files of all the plasmids, generated with the plasmid editor ApE in GenBank format, are included in the Supplementary Information.

### Cell Lines

We started with U-2 OS cells grown at 37 °C in 5% CO_2_ atmosphere in Eagle’s Minimum Essential Medium (EMEM) (ATCC) supplemented with 10% FBS (Invitrogen). To create a stable cell line that expresses MCP-EYFP, PCP-tagRFP, and dCas9, we first co-infected U-2 OS cells with lentiviruses generated from the pLVX-MCP-EYFP and pLVX-PCP-tagRFP plasmids. For lentivirus production and infection, we followed Clontech’s recommended protocol. The infected cells were selected by adding 3 μg/mL puromycin (Thermo Fisher) to the medium. A single cell was expanded for further cloning to ensure relatively uniform MCP-EYFP and PCP-tagRFP expression. Next, this clone was infected with lentiviruses generated from the pLVX-dCas9-ECFP plasmid, and selected by adding 250 μg/mL zeocin (Thermo Fisher) to the medium. Finally, the selected cells were sorted with a MoFlo Astrios cell sorter (Beckman Coulter) for the ECFP-positive subpopulation.

### Transfection and imaging sample preparation

U-2 OS cells stably expressing MCP-EYFP, PCP-tagRFP and dCas9-ECFP were plated on LabTek II eight-well chambered cover glass (Nunc) at a density of 50,000 cells per well, and grown in Minimum Essential Media (Gibco) with 10% FBS (Invitrogen) for 24 hours. Then the cells were either transfected with one sgRNA plasmid, or co-transfected with equal amounts of two plasmids carrying the MS2/PP7 tagged sgRNA respectively. We used the jetPRIME transfection reagent (Polyplus) and followed the manufacturer’s protocol. 24 hours after transfection, the cells were imaged alive in Dulbecco’s phosphate-buffered saline (DPBS).

### Immunofluorescence labeling of telomeric protein

The immunofluorescence labeling protocol was the same as previously described^23^. For the primary antibody, we used mouse anti-TRF1 diluted 1:200 (Abcam Ab10579). For the secondary antibody, we used donkey anti-mouse Alexa 647 diluted 1:1000 (ThermoFisher A-31571).

### Optical setup and imaging conditions

Fluorescence imaging was primarily performed on a home-built optical setup consisting of an Olympus IX-71 inverted microscope body with a 100x UPlanSApo NA 1.40 objective lens (Olympus), and an active sample stabilization system described previously^23^. To image EYFP, we used a 514-nm laser (Sapphire 514-50 CW, Coherent) for excitation, an FF520-Di02 (Semrock) dichroic mirror, and an FF01-542/27 (Semrock) emission filter. To image tagRFP, we used a 561-nm laser (Sapphire 561-200CW, Coherent), a Di01-R561 (Semrock) dichroic mirror, and an FF01-617/73 (Semrock) emission filter. To image Alexa 647, we used a 640-nm laser (Genesis MX639-1000 STM, Coherent), a T660LPXR (Chroma) dichroic mirror, and an ET705/72m (Chroma) emission filter. The fluorescence emission was detected with an Ixon DV897DCS-BV camera (Andor). The exposure time for each image was 0.1 sec. The laser power densities at the sample were 0.077 kW/cm^2^ at 514 nm, 0.042 kW/cm^2^ at 561 nm, and 0.035 kW/cm^2^ at 640 nm.

The ECFP/EYFP/tagRFP three-color imaging was performed on a Zeiss Cell Observer microscope with a 63x PlanApo NA 1.40 objective lens (Zeiss). The samples were illuminated with sola light engine (Lumencor) at 3.0 V. For ECFP imaging, we used an HE47 – ExBP 436/25, FT 455, EmBP 480/40 filter cube (Zeiss). For EYFP imaging, we used an HE46 – ExBP 500/20, FT 515, EmBP 535/30 filter cube (Zeiss). For tagRFP imaging, we used an HE63 – ExBP 572/25, FT 590, EmBP 629/62 filter cube (Zeiss). The fluorescence emission was detected with an Orca Flash 4.0 camera (Hamamatsu), and the exposure times were 0.25 sec for ECFP, 2 sec for EYFP, and 1.2 sec for tagRFP.

### Quantification of signal-to-background ratio of telomere foci

Z-stack images of the cell nuclei were taken with a step size of 200 nm. The CCD baseline of the camera was first subtracted from the images. For each telomere, we computationally identified the best focused image in the stack. The telomere image was then fit to a 2D Gaussian profile with a baseline. And the ratio of the peak value of the fit to the baseline value was defined as the signal-to-background ratio of this telomere. All analyses were performed with MATLAB R2012a.

### Counting the total number of foci in each nucleus

Z-stack images were taken with a step size of 200 nm and enough steps to cover the depth of each nucleus. Then the fluorescent foci in each image were computationally identified based on their significantly higher intensity above the surround area. The 2D centroid positions of the foci were determined by fitting to 2D Gaussian profiles. Next, foci that were less than one-pixel (167-nm centroid-to-centroid distance) apart in successive z images were linked and identified as the same focus appearing at different imaging depths. The total number of foci was determined after these procedures.

## Additional Information

**How to cite this article**: Wang, S. *et al.* An RNA-aptamer-based two-color CRISPR labeling system. *Sci. Rep.* **6**, 26857; doi: 10.1038/srep26857 (2016).

## Supplementary Material

Supplementary Information

## Figures and Tables

**Table 1:** List of new plasmids presented in this work.

Plasmids	Discription
pLVX-MCP-EYFP	*mcp::eyfp* fusion under the control of a *UbC* promoter, cloned into a Clontech pLVX-TRE3G backbone, replacing the *TRE3GV* promoter.
pLVX-PCP-tagRFP	*pcp::tagRFP* fusion under the control of a *UbC* promoter, cloned into a Clontech pLVX-TRE3G backbone, replacing the *TRE3GV* promoter.
pLVX-dCas9-ECFP	*dCas9::ecfp* fusion under the control of a *CMV* promoter, cloned into a Clontech pLVX-TRE3G backbone, replacing the *TRE3GV* promoter. Also the puromycin resistance marker was replaced with a zeocin resistance marker.
psgRNA1.0-6xMS2-telo	sgRNA targeting telomere with 6 *MS2* hairpins at the 3′ end, expressed from a *U6* promoter, cloned into a Clontech pEYFP-N1 backbone, replacing the *CMV* promoter and *eyfp* sequences.
psgRNA1.0-6xPP7-telo	Same as above except with *PP7* hairpins.
psgRNA2.0-6xMS2-telo	sgRNA targeting telomere with 2 *MS2* hairpins extended from the tetraloop and stem loop 2, and 4 more *MS2* hairpins at the 3′ end, expressed from a *U6* promoter, cloned into a Clontech pEYFP-N1 backbone, replacing the *CMV* promoter and *eyfp* sequences.
psgRNA2.0-6xPP7-telo	Same as above except with *PP7* hairpins.
psgRNA2.0-6xPP7-cent	Same as above except with centromere targeting sequence.
psgRNA2.0-6xMS2-cent	Same as above except with *MS2* hairpins.
