# Supplementary material for: An RNA-aptamer-based two-color CRISPR labeling system
Source: Sci Rep. 2016 May 27;6:26857. doi: 10.1038/srep26857 (PMC4882555; doi:10.1038/srep26857)
Supplement: Supplementary Information [file srep26857-s1.pdf]

Supplementary Materials for

An RNA-aptamer-based two-color CRISPR labeling system

Siyuan Wang, Jun-Han Su, Feng Zhang, Xiaowei Zhuang

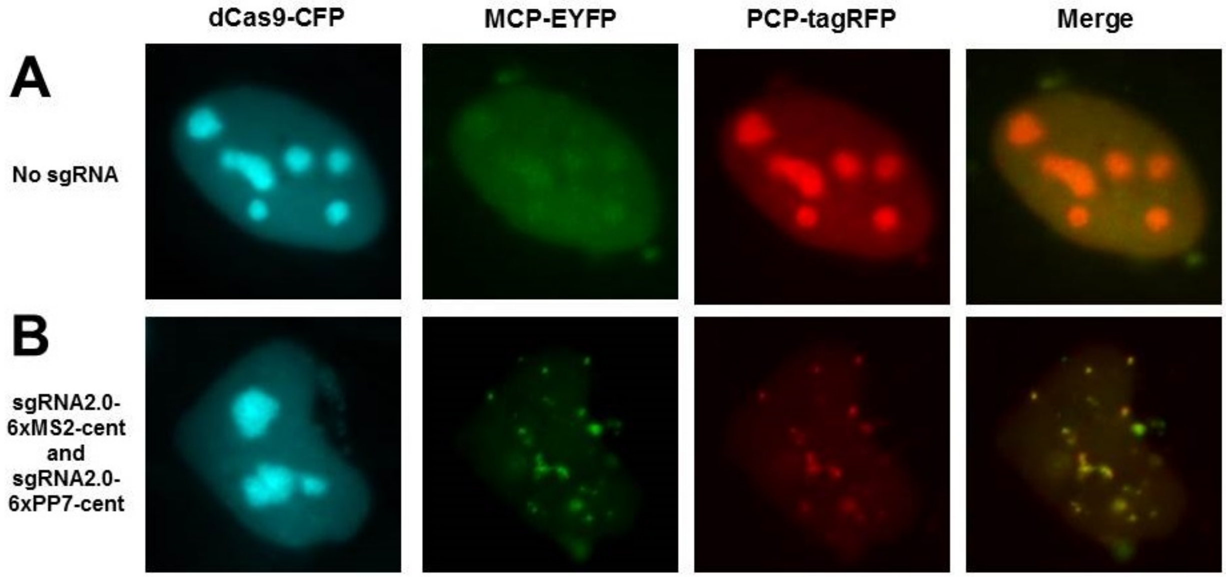

Figure S1. Three-color imaging of the dCas9-ECFP, MCP-EYFP, and PCP-tagRFP. (A) Untransfected cells. (B) Cells co-transfected with sgRNA2.0-6xMS2 and sgRNA2.0-6xPP7 targeting centromeres. First column: CFP fluorescence channel. Second column: EYFP fluorescence channel. Third column: tagRFP fluorescence channel. Fourth column: composite images of the EYFP and tagRFP channels.

Sequence of pLVX-dCas9-ECFP in GenBank format

LOCUS   pLVX_dCas9_ECFP   12765 bp ds-DNA   circular   30-DEC-2015
DEFINITION Lentiviral vector for doxycycline-inducible expression of a gene in
            cells containing the Tet-On 3G transactivator protein.
ACCESSION   .
VERSION   .
KEYWORDS   pLVX-TRE3G
SOURCE   synthetic DNA construct
  ORGANISM synthetic DNA construct
REFERENCE   1 (bases 1 to 7819)
  AUTHORS   Clontech
  TITLE   Direct Submission
  JOURNAL   Exported Friday, Jun 27, 2014 from SnapGene Viewer 2.4.2
            http://www.snapgene.com
COMMENT
COMMENT   ApEinfo:methylated:1
FEATURES   Location/Qualifiers
     LTR   1..634
                     /note="5' LTR"
                     /note="5' long terminal repeat (LTR) from HIV-1"
                     /label=5' LTR
                     /ApEinfo_fwdcolor=pink
                     /ApEinfo_revcolor=pink
                     /ApEinfo_graphicformat=arrow_data {{0 1 2 0 0 -1} {} 0}
                     width 5 offset 0
     misc_feature   681..806
                     /note="HIV-1 psi"
                     /note="packaging signal of human immunodeficiency virus
                     type 1"
                     /label=HIV-1 psi
                     /ApEinfo_fwdcolor=pink
                     /ApEinfo_revcolor=pink
                     /ApEinfo_graphicformat=arrow_data {{0 1 2 0 0 -1} {} 0}
                     width 5 offset 0
     misc_feature   1303..1536
                     /note="RRE"
                     /note="The Rev response element (RRE) of HIV-1 allows for
                     Rev-dependent mRNA export from the nucleus to the
                     cytoplasm."
                     /label=RRE
                     /ApEinfo_fwdcolor=pink
                     /ApEinfo_revcolor=pink
                     /ApEinfo_graphicformat=arrow_data {{0 1 2 0 0 -1} {} 0}
                     width 5 offset 0
     misc_feature   2028..2144
                     /note="cPPT/CTS"
                     /note="central polypurine tract and central termination
                     sequence of HIV-1 (lacking the first T)"
                     /label=cPPT/CTS
                     /ApEinfo_fwdcolor=pink
                     /ApEinfo_revcolor=pink
                     /ApEinfo_graphicformat=arrow_data {{0 1 2 0 0 -1} {} 0}
                     width 5 offset 0
     misc_feature   2908..7008
                     /label=SpCas9 (D10A, H840A)
                     /ApEinfo_fwdcolor=cyan
                     /ApEinfo_revcolor=green
                     /ApEinfo_graphicformat=arrow_data {{0 1 2 0 0 -1} {} 0}
                     width 5 offset 0
     promoter   7775..8274
                     /note="PGK promoter"
                     /note="mouse phosphoglycerate kinase 1 promoter"
                     /label=PGK promoter
                     /ApEinfo_fwdcolor=pink
                     /ApEinfo_revcolor=pink
                     /ApEinfo_graphicformat=arrow_data {{0 1 2 0 0 -1} {} 0}
                     width 5 offset 0
     misc_feature   8687..9275

/note="WPRE"
/note="woodchuck hepatitis virus posttranscriptional
regulatory element"
/label=WPRE
/ApEinfo_fwdcolor=pink
/ApEinfo_revcolor=pink
/ApEinfo_graphicformat=arrow_data {{0 1 2 0 0 -1} {} 0}
width 5 offset 0
LTR 9483..10116
/note="3' LTR"
/note="3' long terminal repeat (LTR) from HIV-1"
/label=3' LTR
/ApEinfo_fwdcolor=pink
/ApEinfo_revcolor=pink
/ApEinfo_graphicformat=arrow_data {{0 1 2 0 0 -1} {} 0}
width 5 offset 0
rep_origin complement(10647..11232)
/direction=LEFT
/note="ori"
/note="high-copy-number ColE1/pMB1/pBR322/pUC origin of
replication"
/label=ori
/ApEinfo_fwdcolor=pink
/ApEinfo_revcolor=pink
/ApEinfo_graphicformat=arrow_data {{0 1 2 0 0 -1} {} 0}
width 5 offset 0
CDS complement(11403..12263)
/codon_start=1
/gene="bla"
/product="beta-lactamase"
/note="AmpR"
/note="confers resistance to ampicillin, carbenicillin,
and related antibiotics"
/translation="MSIQHFRVALIPFFAAFCLPVFAHPETLVKVKDAEDQLGARVGY
I
ELDLNSGKILESFRPEERFPMMSTFKVLLCGAVLSRIDAGQEQLGRRIHYSQNDLVEY
S
PVTEKHLTDGMTVRELCSAAITMSDNTAANLLLTTIGGPKELTAFLHNMGDHVTRLDR
W
EPELNEAIPNDERDTTMPVAMATTLRKLLTGELLTLASRQQLIDWMEADKVAGPLLRS
A
LPAGWFIADKSGAGERGSRGIIAALGPDGKPSRIVVIYTTGSQATMDERNRQIAEIGA
S LIKHW"
/label=beta-lactamase
/ApEinfo_fwdcolor=pink
/ApEinfo_revcolor=pink
/ApEinfo_graphicformat=arrow_data {{0 1 2 0 0 -1} {} 0}
width 5 offset 0
polyA_signal 12416..12550
/note="SV40 poly(A) signal"
/note="SV40 polyadenylation signal"
/label=SV40 poly(A) signal
/ApEinfo_fwdcolor=pink
/ApEinfo_revcolor=pink
/ApEinfo_graphicformat=arrow_data {{0 1 2 0 0 -1} {} 0}
width 5 offset 0
promoter 2719..2788
/label=CMV_promoter
/ApEinfo_fwdcolor=#ccff66
/ApEinfo_revcolor=pink
/ApEinfo_graphicformat=arrow_data {{0 1 2 0 0 -1} {} 0}
width 5 offset 0
misc_feature 7018..7038
/label=NLS
/ApEinfo_fwdcolor=#ff8080
/ApEinfo_revcolor=green
/ApEinfo_graphicformat=arrow_data {{0 1 2 0 0 -1} {} 0}
width 5 offset 0
misc_feature 2264..2551
/label=CAG_enhancer

```
                     /ApEinfo_fwdcolor=pink
                     /ApEinfo_revcolor=pink
                     /ApEinfo_graphicformat=arrow_data {{0 1 2 0 0 -1} {} 0}
                     width 5 offset 0
     misc_feature    2887..2907
                     /label=NLS(1)
                     /ApEinfo_label=NLS
                     /ApEinfo_fwdcolor=#ff8080
                     /ApEinfo_revcolor=green
                     /ApEinfo_graphicformat=arrow_data {{0 1 2 0 0 -1} {} 0}
                     width 5 offset 0
     promoter        2209..2761
                     /label=CMV_immearly_promoter
                     /ApEinfo_fwdcolor=#ccff66
                     /ApEinfo_revcolor=pink
                     /ApEinfo_graphicformat=arrow_data {{0 1 2 0 0 -1} {} 0}
                     width 5 offset 0
     misc_feature    8295..8669
                     /label=ZeoR
                     /ApEinfo_fwdcolor=cyan
                     /ApEinfo_revcolor=green
                     /ApEinfo_graphicformat=arrow_data {{0 1 2 0 0 -1} {} 0}
                     width 5 offset 0
     CDS             7048..7761
                     /gene="ECFP"
                     /note="ORF frame 1"
                     /translation="MVSKGEELFTGVVPILVELDGDVNGHKFSVSGEGEGDATYGKLT
                     LKFICTTGKLPVPWPTLVTTLTWGVQCFSRYPDHMKQHDFFKSAMPEGYVQERTIFFK
                     DDGNYKTRAEVKFEGDTLVNRIELKGIDFKEDGNILGHKLEYNYISHNVYITADKQKN
                     GIKANFKIRHNIEDGSVQLADHYQQNTPIGDGPVLLPDNHYLSTQSALSKDPNEKRDH
                     MVLLEFVTAAGITLGMDELYKSGLRSRAQASNSAVDGTAGPGSTGSR*"
                     /label=ECFP
                     /ApEinfo_fwdcolor=#00ffff
                     /ApEinfo_revcolor=pink
                     /ApEinfo_graphicformat=arrow_data {{0 1 2 0 0 -1} {} 0}
                     width 5 offset 0
ORIGIN
        1 tggaagggct aattcactcc caaagaagac aagatatcct tgatctgtgg atctaccaca
       61 cacaaggcta cttccctgat tagcagaact acacaccagg gccaggggtc agatatccac
      121 tgacctttgg atggtgctac aagctagtac cagttgagcc agataaggta gaagaggcca
      181 ataaaggaga gaacaccagc ttgttacacc ctgtgagcct gcatgggatg gatgacccgg
      241 agagagaagt gttagagtgg aggtttgaca gccgcctagc atttcatcac gtggcccgag
      301 agctgcatcc ggagtacttc aagaactgct gatatcgagc ttgctacaag ggactttccg
      361 ctggggactt tccagggagg cgtggcctgg gcgggactgg ggagtggcga gccctcagat
      421 cctgcatata agcagctgct ttttgcctgt actgggtctc tctggttaga ccagatctga
      481 gcctgggagc tctctggcta actagggaac ccactgctta agcctcaata aagcttgcct
      541 tgagtgcttc aagtagtgtg tgcccgtctg ttgtgtgact ctggtaacta gagatccctc
      601 agaccctttt agtcagtgtg gaaaatctct agcagtggcg cccgaacagg gacttgaaag
      661 cgaaagggaa accagaggag ctctctcgac gcaggactcg gcttgctgaa gcgcgcacgg
      721 caagaggcga ggggcggcga ctggtgagta cgccaaaaat tttgactagc ggaggctaga
      781 aggagagaga tgggtgcgag agcgtcagta ttaagcgggg gagaattaga tcgcgatggg
      841 aaaaaattcg gttaaggcca gggggaaaga aaaaatataa attaaaacat atagtatggg
      901 caagcaggga gctagaacga ttcgcagtta atcctggcct gttagaaaca tcagaaggct
      961 gtagacaaat actgggacag ctacaaccat cccttcagac aggatcagaa gaacttagat
     1021 cattatataa tacagtagca accctctatt gtgtgcatca aaggatagag ataaaagaca
     1081 ccaaggaagc tttagacaag atagaggaag agcaaaacaa aagtaagacc accgcacagc
     1141 aagcggccgg ccgctgatct tcagacctgg aggaggagat atgagggaca attggagaag
     1201 tgaattatat aaatataaag tagtaaaaat tgaaccatta ggagtagcac ccaccaaggc
     1261 aaagagaaga gtggtgcaga gagaaaaaag agcagtggga ataggagctt tgttccttgg
     1321 gttcttggga gcagcaggaa gcactatggg cgcagcgtca atgacgctga cggtacaggc
     1381 cagacaatta ttgtctggta tagtgcagca gcagaacaat ttgctgaggg ctattgaggc
     1441 gcaacagcat ctgttgcaac tcacagtctg gggcatcaag cagctccagg caagaatcct
     1501 ggctgtggaa agatacctaa aggatcaaca gctcctgggg atttggggtt gctctggaaa
     1561 actcatttgc accactgctg tgccttggaa tgctagttgg agtaataaat ctctggaaca
     1621 gatttggaat cacacgacct ggatggagtg ggacagagaa attaacaatt acacaagctt
     1681 aatacactcc ttaattgaag aatcgcaaaa ccagcaagaa aagaatgaac aagaattatt
     1741 ggaattagat aaatgggcaa gtttgtggaa ttggtttaac ataacaaatt ggctgtggta
     1801 tataaaatta ttcataatga tagtaggagg cttggtaggt ttaagaatag tttttgctgt
     1861 actttctata gtgaatagag ttaggcaggg atattcacca ttatcgtttc agacccacct
     1921 cccaaccccg aggggacccg acaggcccga aggaatagaa gaagaaggtg gagagagaga
```

1981 cagagacaga tccattcgat tagtgaacgg atctcgacgg tatcgccttt aaaagaaaag
2041 gggggattgg ggggtacagt gcaggggaaa gaatagtaga cataatagca acagacatac
2101 aaactaaaga actacaaaaa caaattacaa aaattcaaaa ttttcgggtt tattacaggg
2161 acagcagaga tccagtttat cgatgaggcc ctttcgtctt cactcgagAT AGTAATCAAT
2221 TACGGGGTCA TTAGTTCATA GCCCATATAT GGAGTTCCGC GTTACATAAC TTACGGTAAA
2281 TGGCCCGCCT GGCTGACCGC CCAACGACCC CCGCCCATTG ACGTCAATAA TGACGTATGT
2341 TCCCATAGTA ACGCCAATAG GGACTTTCCA TTGACGTCAA TGGGTGGAGT ATTTACGGTA
2401 AACTGCCCAC TTGGCAGTAC ATCAAGTGTA TCATATGCCA AGTACGCCCC CTATTGACGT
2461 CAATGACGGT AAATGGCCCG CCTGGCATTA TGCCCAGTAC ATGACCTTAT GGGACTTTCC
2521 TACTTGGCAG TACATCTACG TATTAGTCAT CGCTATTACC ATGGTGATGC GGTTTTGGCA
2581 GTACATCAAT GGGCGTGGAT AGCGGTTTGA CTCACGGGGA TTTCCAAGTC TCCACCCCAT
2641 TGACGTCAAT GGGAGTTTGT TTTGGCACCA AAATCAACGG GACTTTCCAA AATGTCGTAA
2701 CAACTCCGCC CCATTGACGC AAATGGGCGG TAGGCGTGTA CGGTGGGAGG TCTATATAAG
2761 CAGAGCTGGT TTAGTGAACC GTCAGATCCG CTAGCGCTAC CGGACTCAGA TCTCGAGCTC
2821 AAGCTTCGAA TTCTGCAGTC GACGGTACCG CGGGCCCGGG ATCCACCGGT CGCCACCATG
2881 GCTAGCCCCA AAAAGAAGAG GAAAGTGGAC AAGAAGTACA GCATCGGCCT GGCCATCGGC
2941 ACCAACTCTG TGGGCTGGGC CGTGATCACC GACGAGTACA AGGTGCCCAG CAAGAAATTC
3001 AAGGTGCTGG GCAACACCGA CCGGCACAGC ATCAAGAAGA ACCTGATCGG AGCCCTGCTG
3061 TTCGACAGCG GCGAAACAGC CGAGGCCACC CGGCTGAAGA GAACCGCCAG AAGAAGATAC
3121 ACCAGACGGA AGAACCGGAT CTGCTATCTG CAAGAGATCT TCAGCAACGA GATGGCCAAG
3181 GTGGACGACA GCTTCTTCCA CAGACTGGAA GAGTCCTTCC TGGTGGAAGA GGATAAGAAG
3241 CACGAGCGGC ACCCCATCTT CGGCAACATC GTGGACGAGG TGGCCTACCA CGAGAAGTAC
3301 CCCACCATCT ACCACCTGAG AAAGAAACTG GTGGACAGCA CCGACAAGGC CGACCTGCGG
3361 CTGATCTATC TGGCCCTGGC CCACATGATC AAGTTCCGGG GCCACTTCCT GATCGAGGGC
3421 GACCTGAACC CCGACAACAG CGACGTGGAC AAGCTGTTCA TCCAGCTGGT GCAGACCTAC
3481 AACCAGCTGT TCGAGGAAAA CCCCATCAAC GCCAGCGGCG TGGACGCCAA GGCCATCCTG
3541 TCTGCCAGAC TGAGCAAGAG CAGACGGCTG GAAAATCTGA TCGCCCAGCT GCCCGGCGAG
3601 AAGAAGAATG GCCTGTTCGG CAACCTGATT GCCCTGAGCC TGGGCCTGAC CCCCAACTTC
3661 AAGAGCAACT TCGACCTGGC CGAGGATGCC AAACTGCAGC TGAGCAAGGA CACCTACGAC
3721 GACGACCTGG ACAACCTGCT GGCCCAGATC GGCGACCAGT ACGCCGACCT GTTTCTGGCC
3781 GCCAAGAACC TGTCCGACGC CATCCTGCTG AGCGACATCC TGAGAGTGAA CACCGAGATC
3841 ACCAAGGCCC CCCTGAGCGC CTCTATGATC AAGAGATACG ACGAGCACCA CCAGGACCTG
3901 ACCCTGCTGA AAGCTCTCGT GCGGCAGCAG CTGCCTGAGA AGTACAAAGA GATTTTCTTC
3961 GACCAGAGCA AGAACGGCTA CGCCGGCTAC ATTGACGGCG GAGCCAGCCA GGAAGAGTTC
4021 TACAAGTTCA TCAAGCCCAT CCTGGAAAAG ATGGACGGCA CCGAGGAACT GCTCGTGAAG
4081 CTGAACAGAG AGGACCTGCT GCGGAAGCAG CGGACCTTCG ACAACGGCAG CATCCCCCAC
4141 CAGATCCACC TGGGAGAGCT GCACGCCATT CTGCGGCGGC AGGAAGATTT TTACCCATTC
4201 CTGAAGGACA ACCGGGAAAA GATCGAGAAG ATCCTGACCT TCCGCATCCC CTACTACGTG
4261 GGCCCTCTGG CCAGGGGAAA CAGCAGATTC GCCTGGATGA CCAGAAAGAG CGAGGAAACC
4321 ATCACCCCCT GGAACTTCGA GGAAGTGGTG GACAAGGGCG CTTCCGCCCA GAGCTTCATC
4381 GAGCGGATGA CCAACTTCGA TAAGAACCTG CCCAACGAGA AGGTGCTGCC CAAGCACAGC
4441 CTGCTGTACG AGTACTTCAC CGTGTATAAC GAGCTGACCA AAGTGAAATA CGTGACCGAG
4501 GGAATGAGAA AGCCCGCCTT CCTGAGCGGC GAGCAGAAAA AGGCCATCGT GGACCTGCTG
4561 TTCAAGACCA ACCGGAAAGT GACCGTGAAG CAGCTGAAAG AGGACTACTT CAAGAAAATC
4621 GAGTGCTTCG ACTCCGTGGA AATCTCCGGC GTGGAAGATC GGTTCAACGC CTCCCTGGGC
4681 ACATACCACG ATCTGCTGAA AATTATCAAG GACAAGGACT TCCTGGACAA TGAGGAAAAC
4741 GAGGACATTC TGGAAGATAT CGTGCTGACC CTGACACTGT TTGAGGACAG AGAGATGATC
4801 GAGGAACGGC TGAAAACCTA TGCCCACCTG TTCGACGACA AAGTGATGAA GCAGCTGAAG
4861 CGGCGGAGAT ACACCGGCTG GGGCAGGCTG AGCCGGAAGC TGATCAACGG CATCCGGGAC
4921 AAGCAGTCCG GCAAGACAAT CCTGGATTTC CTGAAGTCCG ACGGCTTCGC CAACAGAAAC
4981 TTCATGCAGC TGATCCACGA CGACAGCCTG ACCTTTAAAG AGGACATCCA GAAAGCCCAG
5041 GTGTCCGGCC AGGGCGATAG CCTGCACGAG CACATTGCCA ATCTGGCCGG CAGCCCCGCC
5101 ATTAAGAAGG GCATCCTGCA GACAGTGAAG GTGGTGGACG AGCTCGTGAA AGTGATGGGC
5161 CGGCACAAGC CCGAGAACAT CGTGATCGAA ATGGCCAGAG AGAACCAGAC CACCCAGAAG
5221 GGACAGAAGA ACAGCCGCGA GAGAATGAAG CGGATCGAAG AGGGCATCAA AGAGCTGGGC
5281 AGCCAGATCC TGAAAGAACA CCCCGTGGAA AACACCCAGC TGCAGAACGA GAAGCTGTAC
5341 CTGTACTACC TGCAGAATGG GCGGGATATG TACGTGGACC AGGAACTGGA CATCAACCGG
5401 CTGTCCGACT ACGATGTGGA CGCTATCGTG CCTCAGAGCT TTCTGAAGGA CGACTCCATC
5461 GACAACAAGG TGCTGACCAG AAGCGACAAG AACCGGGGCA AGAGCGACAA CGTGCCCTCC
5521 GAAGAGGTCG TGAAGAAGAT GAAGAACTAC TGGCGGCAGC TGCTGAACGC CAAGCTGATT
5581 ACCCAGAGAA AGTTCGACAA TCTGACCAAG GCCGAGAGAG GCGGCCTGAG CGAACTGGAT
5641 AAGGCCGGCT TCATCAAGAG ACAGCTGGTG GAAACCCGGC AGATCACAAA GCACGTGGCA
5701 CAGATCCTGG ACTCCCGGAT GAACACTAAG TACGACGAGA ATGACAAGCT GATCCGGGAA
5761 GTGAAAGTGA TCACCCTGAA GTCCAAGCTG GTGTCCGATT TCCGGAAGGA TTTCCAGTTT
5821 TACAAAGTGC GCGAGATCAA CAACTACCAC CACGCCCACG ACGCCTACCT GAACGCCGTC
5881 GTGGGAACCG CCCTGATCAA AAAGTACCCT AAGCTGGAAA GCGAGTTCGT GTACGGCGAC
5941 TACAAGGTGT ACGACGTGCG GAAGATGATC GCCAAGAGCG AGCAGGAAAT CGGCAAGGCT
6001 ACCGCCAAGT ACTTCTTCTA CAGCAACATC ATGAACTTTT TCAAGACCGA GATTACCCTG
6061 GCCAACGGCG AGATCCGGAA GCGGCCTCTG ATCGAGACAA ACGGCGAAAC CGGGGAGATC
6121 GTGTGGGATA AGGGCCGGGA TTTTGCCACC GTGCGGAAAG TGCTGAGCAT GCCCCAAGTG

6181 AATATCGTGA AAAAGACCGA GGTGCAGACA GGCGGCTTCA GCAAAGAGTC TATCCTGCCC
6241 AAGAGGAACA GCGATAAGCT GATCGCCAGA AAGAAGGACT GGGACCCTAA GAAGTACGGC
6301 GGCTTCGACA GCCCCACCGT GGCCTATTCT GTGCTGGTGG TGGCCAAAGT GGAAAAGGGC
6361 AAGTCCAAGA AACTGAAGAG TGTGAAAGAG CTGCTGGGGA TCACCATCAT GGAAAGAAGC
6421 AGCTTCGAGA AGAATCCCAT CGACTTTCTG GAAGCCAAGG GCTACAAAGA AGTGAAAAAG
6481 GACCTGATCA TCAAGCTGCC TAAGTACTCC CTGTTCGAGC TGGAAAACGG CCGGAAGAGA
6541 ATGCTGGCCT CTGCCGGCGA ACTGCAGAAG GGAAACGAAC TGGCCCTGCC CTCCAAATAT
6601 GTGAACTTCC TGTACCTGGC CAGCCACTAT GAGAAGCTGA AGGGCTCCCC CGAGGATAAT
6661 GAGCAGAAAC AGCTGTTTGT GGAACAGCAC AAGCACTACC TGGACGAGAT CATCGAGCAG
6721 ATCAGCGAGT TCTCCAAGAG AGTGATCCTG GCCGACGCTA ATCTGGACAA AGTGCTGTCC
6781 GCCTACAACA AGCACCGGGA TAAGCCCATC AGAGAGCAGG CCGAGAATAT CATCCACCTG
6841 TTTACCCTGA CCAATCTGGG AGCCCCTGCC GCCTTCAAGT ACTTTGACAC CACCATCGAC
6901 CGGAAGAGGT ACACCAGCAC CAAAGAGGTG CTGGACGCCA CCCTGATCCA CCAGAGCATC
6961 ACCGGCCTGT ACGAGACACG GATCGACCTG TCTCAGCTGG GAGGCGACGA GGGAGCTCCC
7021 AAGAAAAAGC GCAAGGTAGG TAGTTCCGTG AGCAAGGGCG AGGAGCTGTT CACCGGGGTG
7081 GTGCCCATCC TGGTCGAGCT GGACGGCGAC GTAAACGGCC ACAAGTTCAG CGTGTCCGGC
7141 GAGGGCGAGG GCGATGCCAC CTACGGCAAG CTGACCCTGA AGTTCATCTG CACCACCGGC
7201 AAGCTGCCCG TGCCCTGGCC CACCCTCGTG ACCACCCTGA CCTGGGGCGT GCAGTGCTTC
7261 AGCCGCTACC CCGACCACAT GAAGCAGCAC GACTTCTTCA AGTCCGCCAT GCCCGAAGGC
7321 TACGTCCAGG AGCGCACCAT CTTCTTCAAG GACGACGGCA ACTACAAGAC CCGCGCCGAG
7381 GTGAAGTTCG AGGGCGACAC CCTGGTGAAC CGCATCGAGC TGAAGGGCAT CGACTTCAAG
7441 GAGGACGGCA ACATCCTGGG GCACAAGCTG GAGTACAACT ACATCAGCCA CAACGTCTAT
7501 ATCACCGCCG ACAAGCAGAA GAACGGCATC AAGGCCAACT TCAAGATCCG CCACAACATC
7561 GAGGACGGCA GCGTGCAGCT CGCCGACCAC TACCAGCAGA ACACCCCCAT CGGCGACGGC
7621 CCCGTGCTGC TGCCCGACAA CCACTACCTG AGCACCCAGT CCGCCCTGAG CAAAGACCCC
7681 AACGAGAAGC GCGATCACAT GGTCCTGCTG GAGTTCGTGA CCGCCGCCGG GATCACTCTC
7741 GGCATGGACG AGCTGTACAA Gtaagaattc taccgggtag gggaggcgct tttcccaagg
7801 cagtctggag catgcgcttt agcagccccg ctgggcactt ggcgctacac aagtggcctc
7861 tggcctcgca cacattccac atccaccggt aggcgccaac cggctccgtt ctttggtggc
7921 cccttcgcgc caccttctac tcctcccta gtcaggaagt tcccccccgc cccgcagctc
7981 gcgtcgtgca ggacgtgaca aatggaagta gcacgtctca ctagtctcgt gcagatggac
8041 agcaccgctg agcaatggaa gcgggtaggc ctttggggca gcggccaata gcagctttgc
8101 tccttcgctt tctgggctca gaggctggga aggggtgggt ccgggggcgg gctcaggggc
8161 gggctcaggg gcggggcggg cgcccgaagg tcctccggag gcccggcatt ctgcacgctt
8221 caaaagcgca cgtctgccgc gctgttctcc tcttcctcat ctccgggcct ttcgacctgc
8281 agcccaagct taccATGGCC AAGTTGACCA GTGCCGTTCC GGTGCTCACC GCGCGCGACG
8341 TCGCCGGAGC GGTCGAGTTC TGGACCGACC GGCTCGGGTT CTCCCGGGAC TTCGTGGAGG
8401 ACGACTTCGC CGGTGTGGTC CGGGACGACG TGACCCTGTT CATCAGCGCG GTCCAGGACC
8461 AGGTGGTGCC GGACAACACC CTGGCCTGGG TGTGGGTGCG CGGCCTGGAC GAGCTGTACG
8521 CCGAGTGGTC GGAGGTCGTG TCCACGAACT TCCGGGACGC CTCCGGGCCG GCCATGACCG
8581 AGATCGGCGA GCAGCCGTGG GGGCGGGAGT TCGCCCTGCG CGACCCGGCC GGCAACTGCG
8641 TGCACTTCGT GGCCGAGGAG CAGGACTGAc ggggcgcgtc tggaacaatc aacctctgga
8701 ttacaaaatt tgtgaaagat tgactggtat tcttaactat gttgctcctt ttacgctatg
8761 tggatacgct gctttaatgc ctttgtatca tgctattgct tcccgtatgg ctttcatttt
8821 ctcctccttg tataaatcct ggttgctgtc tctttatgag gagttgtggc ccgttgtcag
8881 gcaacgtggc gtggtgtgca ctgtgtttgc tgacgcaacc cccactggtt ggggcattgc
8941 caccacctgt cagctccttt ccgggacttt cgctttcccc ctccctattg ccacggcgga
9001 actcatcgcc gcctgccttg cccgctgctg gacaggggct cggctgttgg gcactgacaa
9061 ttccgtggtg ttgtcgggga agctgacgtc ctttccatgg ctgctcgcct gtgttgccac
9121 ctggattctg cgcgggacgt ccttctgcta cgtcccttcg gccctcaatc cagcggacct
9181 tccttcccgc ggcctgctgc cggctctgcg gcctcttccg cgtcttcgcc ttcgccctca
9241 gacgagtcgg atctcccttt gggccgcctc cccgcctgga attaattctg cagtcgagac
9301 ctagaaaaac atggagcaat cacaagtagc aatacagcag ctaccaatgc tgattgtgcc
9361 tggctagaag cacaagagga ggaggaggtg ggttttccca gtcacacctc aggtaccttt
9421 aagaccaatg acttacaagg cagctgtaga tcttagccac tttttaaaag aaaagagggg
9481 actggaaggg ctaattcact cccaacgaag acaagatatc cttgatctgt ggatctacca
9541 cacacaaggc tacttccctg attagcagaa ctacacacca gggccagggg tcagatatcc
9601 actgaccttt ggatggtgct acaagctagt accagttgag ccagataagg tagaagaggc
9661 caataaagga gagaacacca gcttgttaca ccctgtgagc ctgcatggga tggatgaccc
9721 ggagagagaa gtgttagagt ggaggtttga cagccgccta gcatttcatc acgtggcccg
9781 agagctgcat ccggagtact tcaagaactg ctgatatcga gcttgctaca agggactttc
9841 cgctggggac tttccaggga ggcgtggcct gggcgggact ggggagtggc gagccctcag
9901 atcctgcata taagcagctg ctttttgcct gtactgggtc tctctggtta gaccagatct
9961 gagcctggga gctctctggc taactaggga acccactgct taagcctcaa taaagcttgc
10021 cttgagtgct tcaagtagtg tgtgcccgtc tgttgtgtga ctctggtaac tagagatccc
10081 tcagaccctt ttagtcagtg tggaaaatct ctagcagtag tagttcatgt catcttatta
10141 ttcagtattt ataacttgca aagaaatgaa tatcagagag tgagaggcct tgacattgct
10201 agcgttttac cgtcgacctc tagctagagc ttggcgtaat catggtcata gctgtttcct
10261 gtgtgaaatt gttatccgct cacaattcca cacaacatac gagccggaag cataaagtgt
10321 aaagcctggg gtgcctaatg agtgagctaa ctcacattaa ttgcgttgcg ctcactgccc

10381 gctttccagt cgggaaacct gtcgtgccag ctgcattaat gaatcggcca acgcgcgggg
10441 agaggcggtt tgcgtattgg gcgctcttcc gcttcctcgc tcactgactc gctgcgctcg
10501 gtcgttcggc tgcggcgagc ggtatcagct cactcaaagg cggtaatacg gttatccaca
10561 gaatcagggg ataacgcagg aaagaacatg tgagcaaaag gccagcaaaa ggccaggaac
10621 cgtaaaaagg ccgcgttgct ggcgtttttc cataggctcc gcccccctga cgagcatcac
10681 aaaaatcgac gctcaagtca gaggtggcga aacccgacag gactataaag ataccaggcg
10741 tttccccctg gaagctccct cgtgcgctct cctgttccga ccctgccgct taccggatac
10801 ctgtccgcct ttctcccttc gggaagcgtg gcgctttctc atagctcacg ctgtaggtat
10861 ctcagttcgg tgtaggtcgt tcgctccaag ctgggctgtg tgcacgaacc ccccgttcag
10921 cccgaccgct gcgccttatc cggtaactat cgtcttgagt ccaacccggt aagacacgac
10981 ttatcgccac tggcagcagc cactggtaac aggattagca gagcgaggta tgtaggcggt
11041 gctacagagt tcttgaagtg gtggcctaac tacggctaca ctagaagaac agtatttggt
11101 atctgcgctc tgctgaagcc agttaccttc ggaaaaagag ttggtagctc ttgatccggc
11161 aaacaaacca ccgctggtag cggttttttt gtttgcaagc agcagattac gcgcagaaaa
11221 aaaggatctc aagaagatcc tttgatcttt tctacggggt ctgacgctca gtggaacgaa
11281 aactcacgtt aagggatttt ggtcatgaga ttatcaaaaa ggatcttcac ctagatcctt
11341 ttaaattaaa aatgaagttt taaatcaatc taaagtatat atgagtaaac ttggtctgac
11401 agttaccaat gcttaatcag tgaggcacct atctcagcga tctgtctatt tcgttcatcc
11461 atagttgcct gactccccgt cgtgtagata actacgatac gggagggctt accatctggc
11521 cccagtgctg caatgatacc gcgagaccca cgctcaccgg ctccagattt atcagcaata
11581 aaccagccag ccggaagggc cgagcgcaga agtggtcctg caactttatc cgcctccatc
11641 cagtctatta attgttgccg ggaagctaga gtaagtagtt cgccagttaa tagtttgcgc
11701 aacgttgttg ccattgctac aggcatcgtg gtgtcacgct cgtcgtttgg tatggcttca
11761 ttcagctccg gttcccaacg atcaaggcga gttacatgat cccccatgtt gtgcaaaaaa
11821 gcggttagct ccttcggtcc tccgatcgtt gtcagaagta agttggccgc agtgttatca
11881 ctcatggtta tggcagcact gcataattct cttactgtca tgccatccgt aagatgcttt
11941 tctgtgactg gtgagtactc aaccaagtca ttctgagaat agtgtatgcg gcgaccgagt
12001 tgctcttgcc cggcgtcaat acgggataat accgcgccac atagcagaac tttaaaagtg
12061 ctcatcattg gaaaacgttc ttcggggcga aaactctcaa ggatcttacc gctgttgaga
12121 tccagttcga tgtaacccac tcgtgcaccc aactgatctt cagcatcttt tactttcacc
12181 agcgtttctg ggtgagcaaa aacaggaagg caaaatgccg caaaaaaggg aataagggcg
12241 acacggaaat gttgaatact catactcttc ctttttcaat attattgaag catttatcag
12301 ggttattgtc tcatgagcgg atacatattt gaatgtattt agaaaaataa acaaataggg
12361 gttccgcgca catttccccg aaaagtgcca cctgacgtcg acggatcggg agatcaactt
12421 gtttattgca gcttataatg gttacaaata aagcaatagc atcacaaatt tcacaaataa
12481 agcatttttt tcactgcatt ctagttgtgg tttgtccaaa ctcatcaatg tatcttatca
12541 tgtctggatc aactggataa ctcaagctaa ccaaaatcat cccaaacttc ccaccccata
12601 ccctattacc actgccaatt acctagtggt ttcatttact ctaaacctgt gattcctctg
12661 aattattttc attttaaaga aattgtattt gttaaatatg tactacaaac ttagtagttt
12721 ttaaagaaat tgtatttgtt aaatatgtac tacaaactta gtagt
//

Sequence of pLVX-MCP-EYFP in GenBank format

LOCUS pLVX_MCP_EYFP 9924 bp ds-DNA circular 30-DEC-2015
DEFINITION Lentiviral vector for doxycycline-inducible expression of a gene in
cells containing the Tet-On 3G transactivator protein.
ACCESSION .
VERSION .
KEYWORDS pLVX-TRE3G
SOURCE synthetic DNA construct
ORGANISM synthetic DNA construct
REFERENCE 1 (bases 1 to 7819)
AUTHORS Clontech
TITLE Direct Submission
JOURNAL Exported Friday, Jun 27, 2014 from SnapGene Viewer 2.4.2
http://www.snapgene.com
COMMENT
COMMENT

COMMENT   ApEinfo:methylated:1
FEATURES   Location/Qualifiers
misc_feature   3508..4674
/label=MCP-EYFP
/ApEinfo_fwdcolor=cyan
/ApEinfo_revcolor=green
/ApEinfo_graphicformat=arrow_data {{0 1 2 0 0 -1} {} 0}
width 5 offset 0
LTR   1..634
/note="5' LTR"
/note="5' long terminal repeat (LTR) from HIV-1"
/label=5' LTR
/ApEinfo_fwdcolor=pink
/ApEinfo_revcolor=pink
/ApEinfo_graphicformat=arrow_data {{0 1 2 0 0 -1} {} 0}
width 5 offset 0
misc_feature   681..806
/note="HIV-1 psi"
/note="packaging signal of human immunodeficiency virus
type 1"
/label=HIV-1 psi
/ApEinfo_fwdcolor=pink
/ApEinfo_revcolor=pink
/ApEinfo_graphicformat=arrow_data {{0 1 2 0 0 -1} {} 0}
width 5 offset 0
misc_feature   1303..1536
/note="RRE"
/note="The Rev response element (RRE) of HIV-1 allows for
Rev-dependent mRNA export from the nucleus to the
cytoplasm."
/label=RRE
/ApEinfo_fwdcolor=pink
/ApEinfo_revcolor=pink
/ApEinfo_graphicformat=arrow_data {{0 1 2 0 0 -1} {} 0}
width 5 offset 0
misc_feature   2028..2144
/note="cPPT/CTS"
/note="central polypurine tract and central termination
sequence of HIV-1 (lacking the first T)"
/label=cPPT/CTS
/ApEinfo_fwdcolor=pink
/ApEinfo_revcolor=pink
/ApEinfo_graphicformat=arrow_data {{0 1 2 0 0 -1} {} 0}
width 5 offset 0
promoter   4709..5208
/note="PGK promoter"
/note="mouse phosphoglycerate kinase 1 promoter"
/label=PGK promoter
/ApEinfo_fwdcolor=pink
/ApEinfo_revcolor=pink
/ApEinfo_graphicformat=arrow_data {{0 1 2 0 0 -1} {} 0}
width 5 offset 0
promoter   2209..3425
/label=hUbC_promoter
/ApEinfo_fwdcolor=pink
/ApEinfo_revcolor=pink
/ApEinfo_graphicformat=arrow_data {{0 1 2 0 0 -1} {} 0}
width 5 offset 0
CDS   5229..5828
/codon_start=1
/gene="pac from Streptomyces alboniger"
/product="puromycin N-acetyltransferase"
/note="PuroR"
/note="confers resistance to puromycin"
/translation="MTEYKPTVRLATRDDVPRAVRTLAAAFADYPATRHTVDPDRHIE
R
VTELQELFLTRVGLDIGKVWVADDGAAVAVWTTPESVEAGAVFAEIGPRMAELSGSRL
A
AQQQMEGLLAPHRPKEPAWFLATVGVSPDHQGKGLGSAVVLPGVEAAERAGVPAFLET
S APRNLPFYERLGFTVTADVEVPEGPRTWCMTRKPGA"

/label=puromycin N-acetyltransferase
/ApEinfo_fwdcolor=pink
/ApEinfo_revcolor=pink
/ApEinfo_graphicformat=arrow_data {{0 1 2 0 0 -1} {} 0}
width 5 offset 0
misc_feature 5846..6434
/note="WPRE"
/note="woodchuck hepatitis virus posttranscriptional
regulatory element"
/label=WPRE
/ApEinfo_fwdcolor=pink
/ApEinfo_revcolor=pink
/ApEinfo_graphicformat=arrow_data {{0 1 2 0 0 -1} {} 0}
width 5 offset 0
LTR 6642..7275
/note="3' LTR"
/note="3' long terminal repeat (LTR) from HIV-1"
/label=3' LTR
/ApEinfo_fwdcolor=pink
/ApEinfo_revcolor=pink
/ApEinfo_graphicformat=arrow_data {{0 1 2 0 0 -1} {} 0}
width 5 offset 0
rep_origin complement(7806..8391)
/direction=LEFT
/note="ori"
/note="high-copy-number ColE1/pMB1/pBR322/pUC origin of
replication"
/label=ori
/ApEinfo_fwdcolor=pink
/ApEinfo_revcolor=pink
/ApEinfo_graphicformat=arrow_data {{0 1 2 0 0 -1} {} 0}
width 5 offset 0
CDS complement(8562..9422)
/codon_start=1
/gene="bla"
/product="beta-lactamase"
/note="AmpR"
/note="confers resistance to ampicillin, carbenicillin,
and related antibiotics"
/translation="MSIQHFRVALIPFFAAFCLPVFAHPETLVKVKDAEDQLGARVGY
I
ELDLNSGKILESFRPEERFPMMSTFKVLLCGAVLSRIDAGQEQLGRRIHYSQNDLVEY
S
PVTEKHLTDGMTVRELCSAAITMSDNTAANLLLTTIGGPKELTAFLHNMGDHVTRLDR
W
EPELNEAIPNDERDTTMPVAMATTLRKLLTGELLTLASRQQLIDWMEADKVAGPLLRS
A
LPAGWFIADKSGAGERGSRGIIAALGPDGKPSRIVVIYTTGSQATMDERNRQIAEIGA
S LIKHW"
/label=beta-lactamase
/ApEinfo_fwdcolor=pink
/ApEinfo_revcolor=pink
/ApEinfo_graphicformat=arrow_data {{0 1 2 0 0 -1} {} 0}
width 5 offset 0
misc_feature 3538..3564
/translation="MGPKKKRKVGYPYDVPDYAIEGRHMLAVKMASNFTQFVLVDNGG
TGDVTVAPSNFANGIAEWISSNSRSQAYKVTCSVRQSSAQNRKYTIKVEVPKGAWRSY
LNMELTIPIFATNSDCELIVKAMQGLLKDGNPIPSAIAANSGIYADSRMVSKGEELFT
GVVPILVELDGDVNGHKFSVSGEGEGDATYGKLTLKFICTTGKLPVPWPTLVTTFGYG
LQCFARYPDHMKQHDFFKSAMPEGYVQERTIFFKDDGNYKTRAEVKFEGDTLVNRIEL
KGIDFKEDGNILGHKLEYNYNSHNVYIMADKQKNGIKVNFKIRHNIEDGSVQLADHYQ
QNTPIGDGPVLLPDNHYLSYQSALSKDPNEKRDHMVLLEFVTAAGITLGMDELYK*"
/label=HA
/ApEinfo_fwdcolor=pink
/ApEinfo_revcolor=pink
/ApEinfo_graphicformat=arrow_data {{0 1 2 0 0 -1} {} 0}
width 5 offset 0
polyA_signal 9575..9709
/note="SV40 poly(A) signal"
/note="SV40 polyadenylation signal"

```
                 /label=SV40 poly(A) signal
                 /ApEinfo_fwdcolor=pink
                 /ApEinfo_revcolor=pink
                 /ApEinfo_graphicformat=arrow_data {{0 1 2 0 0 -1} {} 0}
                 width 5 offset 0
     gene            3961..4668
                 /gene="EYFP"
                 /translation="MGPKKKRKVGYPYDVPDYAIEGRHMLAVKMASNFTQFVLVDNGG
                 TGDVTVAPSNFANGIAEWISSNSRSQAYKVTCSVRQSSAQNRKYTIKVEVPKGAWRSY
                 LNMELTIPIFATNSDCELIVKAMQGLLKDGNPIPSAIAANSGIYADSRMVSKGEELFT
                 GVVPILVELDGDVNGHKFSVSGEGEGDATYGKLTLKFICTTGKLPVPWPTLVTTFGYG
                 LQCFARYPDHMKQHDFFKSAMPEGYVQERTIFFKDDGNYKTRAEVKFEGDTLVNRIEL
                 KGIDFKEDGNILGHKLEYNYNSHNVYIMADKQKNGIKVNFKIRHNIEDGSVQLADHYQ
                 QNTPIGDGPVLLPDNHYLSYQSALSKDPNEKRDHMVLLEFVTAAGITLGMDELYK*"
                 /label=EYFP
                 /ApEinfo_fwdcolor=#ff8040
                 /ApEinfo_revcolor=pink
                 /ApEinfo_graphicformat=arrow_data {{0 1 2 0 0 -1} {} 0}
                 width 5 offset 0
     misc_feature    3514..3537
                 /label=SV40 NLS
                 /ApEinfo_fwdcolor=#ff8080
                 /ApEinfo_revcolor=green
                 /ApEinfo_graphicformat=arrow_data {{0 1 2 0 0 -1} {} 0}
                 width 5 offset 0
ORIGIN
        1 tggaagggct aattcactcc caaagaagac aagatatcct tgatctgtgg atctaccaca
       61 cacaaggcta cttccctgat tagcagaact acacaccagg gccaggggtc agatatccac
      121 tgacctttgg atggtgctac aagctagtac cagttgagcc agataaggta gaagaggcca
      181 ataaaggaga gaacaccagc ttgttacacc ctgtgagcct gcatgggatg gatgacccgg
      241 agagagaagt gttagagtgg aggtttgaca gccgcctagc atttcatcac gtggcccgag
      301 agctgcatcc ggagtacttc aagaactgct gatatcgagc ttgctacaag ggactttccg
      361 ctggggactt tccagggagg cgtggcctgg gcgggactgg ggagtggcga gccctcagat
      421 cctgcatata agcagctgct ttttgcctgt actgggtctc tctggttaga ccagatctga
      481 gcctgggagc tctctggcta actagggaac ccactgctta agcctcaata aagcttgcct
      541 tgagtgcttc aagtagtgtg tgcccgtctg ttgtgtgact ctggtaacta gagatccctc
      601 agaccctttt agtcagtgtg gaaaatctct agcagtggcg cccgaacagg gacttgaaag
      661 cgaaagggaa accagaggag ctctctcgac gcaggactcg gcttgctgaa gcgcgcacgg
      721 caagaggcga ggggcggcga ctggtgagta cgccaaaaat tttgactagc ggaggctaga
      781 aggagagaga tgggtgcgag agcgtcagta ttaagcgggg gagaattaga tcgcgatggg
      841 aaaaaattcg gttaaggcca gggggaaaga aaaaatataa attaaaacat atagtatggg
      901 caagcaggga gctagaacga ttcgcagtta atcctggcct gttagaaaca tcagaaggct
      961 gtagacaaat actgggacag ctacaaccat cccttcagac aggatcagaa gaacttagat
     1021 cattatataa tacagtagca accctctatt gtgtgcatca aaggatagag ataaaagaca
     1081 ccaaggaagc tttagacaag atagaggaag agcaaaacaa aagtaagacc accgcacagc
     1141 aagcggccgg ccgctgatct tcagacctgg aggaggagat atgagggaca attggagaag
     1201 tgaattatat aaatataaag tagtaaaaat tgaaccatta ggagtagcac ccaccaaggc
     1261 aaagagaaga gtggtgcaga gagaaaaaag agcagtggga ataggagctt tgttccttgg
     1321 gttcttggga gcagcaggaa gcactatggg cgcagcgtca atgacgctga cggtacaggc
     1381 cagacaatta ttgtctggta tagtgcagca gcagaacaat ttgctgaggg ctattgaggc
     1441 gcaacagcat ctgttgcaac tcacagtctg gggcatcaag cagctccagg caagaatcct
     1501 ggctgtggaa agatacctaa aggatcaaca gctcctgggg atttggggtt gctctggaaa
     1561 actcatttgc accactgctg tgccttggaa tgctagttgg agtaataaat ctctggaaca
     1621 gatttggaat cacacgacct ggatggagtg ggacagagaa attaacaatt acacaagctt
     1681 aatacactcc ttaattgaag aatcgcaaaa ccagcaagaa aagaatgaac aagaattatt
     1741 ggaattagat aaatgggcaa gtttgtggaa ttggtttaac ataacaaatt ggctgtggta
     1801 tataaaatta ttcataatga tagtaggagg cttggtaggt ttaagaatag tttttgctgt
     1861 actttctata gtgaatagag ttaggcaggg atattcacca ttatcgtttc agacccacct
     1921 cccaaccccg aggggacccg acaggcccga aggaatagaa gaagaaggtg gagagagaga
     1981 cagagacaga tccattcgat tagtgaacgg atctcgacgg tatcgccttt aaaagaaaag
     2041 gggggattgg ggggtacagt gcaggggaaa gaatagtaga cataatagca acagacatac
     2101 aaactaaaga actacaaaaa caaattacaa aaattcaaaa ttttcgggtt tattacaggg
     2161 acagcagaga tccagtttat cgatgaggcc ctttcgtctt cactcgagGA TCTGGCCTCC
     2221 GCGCCGGGTT TTGGCGCCTC CCGCGGGCGC CCCCCTCCTC ACGGCGAGCG CTGCCACGTC
     2281 AGACGAAGGG CGCAGCGAGC GTCCTGATCC TTCCGCCCGG ACGCTCAGGA CAGCGGCCCG
     2341 CTGCTCATAA GACTCGGCCT TAGAACCCCA GTATCAGCAG AAGGACATTT TAGGACGGGA
     2401 CTTGGGTGAC TCTAGGGCAC TGGTTTTCTT TCCAGAGAGC GGAACAGGCG AGGAAAAGTA
     2461 GTCCCTTCTC GGCGATTCTG CGGAGGGATC TCCGTGGGGC GGTGAACGCC GATGATTATA
     2521 TAAGGACGCG CCGGGTGTGG CACAGCTAGT TCCGTCGCAG CCGGGATTTG GGTCGCGGTT
     2581 CTTGTTTGTG GATCGCTGTG ATCGTCACTT GGTGAGTAGC GGGCTGCTGG GCTGGCCGGG
```

2641 GCTTTCGTGG CCGCCGGGCC GCTCGGTGGG ACGGAAGCGT GTGGAGAGAC CGCCAAGGGC
2701 TGTAGTCTGG GTCCGCGAGC AAGGTTGCCC TGAACTGGGG GTTGGGGGGA GCGCAGCAAA
2761 ATGGCGGCTG TTCCCGAGTC TTGAATGGAA GACGCTTGTG AGGCGGGCTG TGAGGTCGTT
2821 GAAACAAGGT GGGGGGCATG GTGGGCGGCA AGAACCCAAG GTCTTGAGGC CTTCGCTAAT
2881 GCGGGAAAGC TCTTATTCGG GTGAGATGGG CTGGGGCACC ATCTGGGGAC CCTGACGTGA
2941 AGTTTGTCAC TGACTGGAGA ACTCGGGTTT GTCGTCTGGT TGCGGGGGCG GCAGTTATGC
3001 GGTGCCGTTG GGCAGTGCAC CCGTACCTTT GGGAGCGCGC GCCTCGTCGT GTCGTGACGT
3061 CACCCGTTCT GTTGGCTTAT AATGCAGGGT GGGGCCACCT GCCGGTAGGT GTGCGGTAGG
3121 CTTTTCTCCG TCGCAGGACG CAGGGTTCGG GCCTAGGGTA GGCTCTCCTG AATCGACAGG
3181 CGCCGGACCT CTGGTGAGGG GAGGGATAAG TGAGGCGTCA GTTTCTTTGG TCGGTTTTAT
3241 GTACCTATCT TCTTAAGTAG CTGAAGCTCC GGTTTTGAAC TATGCGCTCG GGGTTGGCGA
3301 GTGTGTTTTG TGAAGTTTTT TAGGCACCTT TTGAAATGTA ATCATTTGGG TCAATATGTA
3361 ATTTTCAGTG TTAGACTAGT AAATTGTCCG CTAAATTCTG GCCGTTTTTG GCTTTTTTGT
3421 TAGACGAAGC TTGGGCTGCA GGTCGAGCTC AAGCTTCGAA TTCTGCAGTC GACGGTACCG
3481 CGGGCCCGGG ATCCACCGGT CGCCACCATG GGCCCAAAAA AGAAAAGAAA AGTTGGCTAC
3541 CCCTACGACG TGCCCGACTA CGCCATCGAA GGCCGCCATA TGCTAGCCGT TAAAATGGCT
3601 TCTAACTTTA CTCAGTTCGT TCTCGTCGAC AATGGCGGAA CTGGCGACGT GACTGTCGCC
3661 CCAAGCAACT TCGCTAACGG GATCGCTGAA TGGATCAGCT CTAACTCGCG TTCACAGGCT
3721 TACAAAGTAA CCTGTAGCGT TCGTCAGAGC TCTGCGCAGA ATCGCAAATA CACCATCAAA
3781 GTCGAGGTGC CTAAAGGCGC CTGGCGTTCG TACTTAAATA TGGAACTAAC CATTCCAATT
3841 TTCGCCACGA ATTCCGACTG CGAGCTTATT GTTAAGGCAA TGCAAGGTCT CCTAAAAGAT
3901 GGAAACCCGA TTCCCTCAGC AATCGCAGCA AACTCCGGCA TCTACGCGGA TTCTAGAATG
3961 GTGAGCAAGG GCGAGGAGCT GTTCACCGGG GTGGTGCCCA TCCTGGTCGA GCTGGACGGC
4021 GACGTAAACG GCCACAAGTT CAGCGTGTCC GGCGAGGGCG AGGGCGATGC CACCTACGGC
4081 AAGCTGACCC TGAAGTTCAT CTGCACCACC GGCAAGCTGC CCGTGCCCTG GCCCACCCTC
4141 GTGACCACCT TCGGCTACGG CCTGCAGTGC TTCGCCCGCT ACCCCGACCA CATGAAGCAG
4201 CACGACTTCT TCAAGTCCGC CATGCCCGAA GGCTACGTCC AGGAGCGCAC CATCTTCTTC
4261 AAGGACGACG GCAACTACAA GACCCGCGCC GAGGTGAAGT TCGAGGGCGA CACCCTGGTG
4321 AACCGCATCG AGCTGAAGGG CATCGACTTC AAGGAGGACG GCAACATCCT GGGGCACAAG
4381 CTGGAGTACA ACTACAACAG CCACAACGTC TATATCATGG CCGACAAGCA GAAGAACGGC
4441 ATCAAGGTGA ACTTCAAGAT CCGCCACAAC ATCGAGGACG GCAGCGTGCA GCTCGCCGAC
4501 CACTACCAGC AGAACACCCC CATCGGCGAC GGCCCCGTGC TGCTGCCCGA CAACCACTAC
4561 CTGAGCTACC AGTCCGCCCT GAGCAAAGAC CCCAACGAGA AGCGCGATCA CATGGTCCTG
4621 CTGGAGTTCG TGACCGCCGC CGGGATCACT CTCGGCATGG ACGAGCTGTA CAAGTAAtaa
4681 ATAGTAATCA ATTACGGGga attctaccgg gtaggggagg cgcttttccc aaggcagtct
4741 ggagcatgcg ctttagcagc cccgctgggc acttggcgct acacaagtgg cctctggcct
4801 cgcacacatt ccacatccac cggtaggcgc caaccggctc cgttctttgg tggccccttc
4861 gcgccacctt ctactcctcc cctagtcagg aagttccccc ccgccccgca gctcgcgtcg
4921 tgcaggacgt gacaaatgga agtagcacgt ctcactagtc tcgtgcagat ggacagcacc
4981 gctgagcaat ggaagcgggt aggcctttgg ggcagcggcc aatagcagct ttgctccttc
5041 gctttctggg ctcagaggct gggaaggggt gggtccgggg gcgggctcag gggcgggctc
5101 aggggcgggg cgggcgcccg aaggtcctcc ggaggcccgg cattctgcac gcttcaaaag
5161 cgcacgtctg ccgcgctgtt ctcctcttcc tcatctccgg gcctttcgac ctgcagccca
5221 agcttaccat gaccgagtac aagcccacgg tgcgcctcgc cacccgcgac gacgtcccca
5281 gggccgtacg caccctcgcc gccgcgttcg ccgactaccc cgccacgcgc cacaccgtcg
5341 atccggaccg ccacatcgag cgggtcaccg agctgcaaga actcttcctc acgcgcgtcg
5401 ggctcgacat cggcaaggtg tgggtcgcgg acgacggcgc cgcggtggcg gtctggacca
5461 cgccggagag cgtcgaagcg ggggcggtgt tcgccgagat cggcccgcgc atggccgagt
5521 tgagcggttc ccggctggcc gcgcagcaac agatggaagg cctcctggcg ccgcaccggc
5581 ccaaggagcc cgcgtggttc ctggccaccg tcggcgtctc gcccgaccac cagggcaagg
5641 gtctgggcag cgccgtcgtg ctccccggag tggaggcggc cgagcgcgcc ggggtgcccg
5701 ccttcctgga gacctccgcg ccccgcaacc tccccttcta cgagcggctc ggcttcaccg
5761 tcaccgccga cgtcgaggtg cccgaaggac cgcgcacctg gtgcatgacc cgcaagcccg
5821 gtgcctgacg gggcgcgtct ggaacaatca acctctggat tacaaaattt gtgaaagatt
5881 gactggtatt cttaactatg ttgctccttt tacgctatgt ggatacgctg ctttaatgcc
5941 tttgtatcat gctattgctt cccgtatggc tttcattttc tcctccttgt ataaatcctg
6001 gttgctgtct ctttatgagg agttgtggcc cgttgtcagg caacgtggcg tggtgtgcac
6061 tgtgtttgct gacgcaaccc ccactggttg gggcattgcc accacctgtc agctcctttc
6121 cgggactttc gctttccccc tccctattgc cacggcggaa ctcatcgccg cctgccttgc
6181 ccgctgctgg acaggggctc ggctgttggg cactgacaat tccgtggtgt tgtcggggaa
6241 gctgacgtcc tttccatggc tgctcgcctg tgttgccacc tggattctgc gcgggacgtc
6301 cttctgctac gtcccttcgg ccctcaatcc agcggacctt ccttcccgcg gcctgctgcc
6361 ggctctgcgg cctcttccgc gtcttcgcct tcgccctcag acgagtcgga tctccctttg
6421 ggccgcctcc ccgcctggaa ttaattctgc agtcgagacc tagaaaaaca tggagcaatc
6481 acaagtagca atacagcagc taccaatgct gattgtgcct ggctagaagc acaagaggag
6541 gaggaggtgg gtttttccag tcacacctca ggtaccttta agaccaatga cttacaaggc
6601 agctgtagat cttagccact tttaaaaga aaagagggga ctggaagggc taattcactc
6661 ccaacgaaga caagatatcc ttgatctgtg gatctaccac acacaaggct acttccctga
6721 ttagcagaac tacacaccag ggccaggggt cagatatcca ctgacctttg gatggtgcta
6781 caagctagta ccagttgagc cagataaggt agaagaggcc aataaaggag agaacaccag

```
    6841 cttgttacac cctgtgagcc tgcatgggat ggatgacccg gagagagaag tgttagagtg
    6901 gaggtttgac agccgcctag catttcatca cgtggcccga gagctgcatc cggagtactt
    6961 caagaactgc tgatatcgag cttgctacaa gggactttcc gctggggact ttccagggag
    7021 gcgtggcctg ggcgggactg gggagtggcg agccctcaga tcctgcatat aagcagctgc
    7081 tttttgcctg tactgggtct ctctggttag accagatctg agcctgggag ctctctggct
    7141 aactagggaa cccactgctt aagcctcaat aaagcttgcc ttgagtgctt caagtagtgt
    7201 gtgcccgtct gttgtgtgac tctggtaact agagatccct cagacccttt tagtcagtgt
    7261 ggaaaatctc tagcagtagt agttcatgtc atcttattat tcagtattta taacttgcaa
    7321 agaaatgaat atcagagagt gagaggcctt gacattgcta gcgttttacc gtcgacctct
    7381 agctagagct tggcgtaatc atggtcatag ctgtttcctg tgtgaaattg ttatccgctc
    7441 acaattccac acaacatacg agccggaagc ataaagtgta aagcctgggg tgcctaatga
    7501 gtgagctaac tcacattaat tgcgttgcgc tcactgcccg ctttccagtc gggaaacctg
    7561 tcgtgccagc tgcattaatg aatcggccaa cgcgcgggga gaggcggttt gcgtattggg
    7621 cgctcttccg cttcctcgct cactgactcg ctgcgctcgg tcgttcggct gcggcgagcg
    7681 gtatcagctc actcaaaggc ggtaatacgg ttatccacag aatcagggga taacgcagga
    7741 aagaacatgt gagcaaaagg ccagcaaaag gccaggaacc gtaaaaaggc cgcgttgctg
    7801 gcgtttttcc ataggctccg cccccctgac gagcatcaca aaaatcgacg ctcaagtcag
    7861 aggtggcgaa acccgacagg actataaaga taccaggcgt ttccccctgg aagctccctc
    7921 gtgcgctctc ctgttccgac cctgccgctt accggatacc tgtccgcctt tctcccttcg
    7981 ggaagcgtgg cgctttctca tagctcacgc tgtaggtatc tcagttcggt gtaggtcgtt
    8041 cgctccaagc tgggctgtgt gcacgaaccc cccgttcagc ccgaccgctg cgccttatcc
    8101 ggtaactatc gtcttgagtc caacccggta agacacgact tatcgccact ggcagcagcc
    8161 actggtaaca ggattagcag agcgaggtat gtaggcggtg ctacagagtt cttgaagtgg
    8221 tggcctaact acggctacac tagaagaaca gtatttggta tctgcgctct gctgaagcca
    8281 gttaccttcg gaaaaagagt tggtagctct tgatccggca aacaaaccac cgctggtagc
    8341 ggtttttttg tttgcaagca gcagattacg cgcagaaaaa aaggatctca agaagatcct
    8401 ttgatctttt ctacggggtc tgacgctcag tggaacgaaa actcacgtta agggattttg
    8461 gtcatgagat tatcaaaaag gatcttcacc tagatccttt taaattaaaa atgaagtttt
    8521 aaatcaatct aaagtatata tgagtaaact tggtctgaca gttaccaatg cttaatcagt
    8581 gaggcaccta tctcagcgat ctgtctattt cgttcatcca tagttgcctg actccccgtc
    8641 gtgtagataa ctacgatacg ggagggctta ccatctggcc ccagtgctgc aatgataccg
    8701 cgagacccac gctcaccggc tccagattta tcagcaataa accagccagc cggaagggcc
    8761 gagcgcagaa gtggtcctgc aactttatcc gcctccatcc agtctattaa ttgttgccgg
    8821 gaagctagag taagtagttc gccagttaat agtttgcgca acgttgttgc cattgctaca
    8881 ggcatcgtgg tgtcacgctc gtcgtttggt atggcttcat tcagctccgg ttcccaacga
    8941 tcaaggcgag ttacatgatc ccccatgttg tgcaaaaaag cggttagctc cttcggtcct
    9001 ccgatcgttg tcagaagtaa gttggccgca gtgttatcac tcatggttat ggcagcactg
    9061 cataattctc ttactgtcat gccatccgta agatgctttt ctgtgactgg tgagtactca
    9121 accaagtcat tctgagaata gtgtatgcgg cgaccgagtt gctcttgccc ggcgtcaata
    9181 cgggataata ccgcgccaca tagcagaact ttaaaagtgc tcatcattgg aaaacgttct
    9241 tcggggcgaa aactctcaag gatcttaccg ctgttgagat ccagttcgat gtaacccact
    9301 cgtgcaccca actgatcttc agcatctttt actttcacca gcgtttctgg gtgagcaaaa
    9361 acaggaaggc aaaatgccgc aaaaaaggga ataagggcga cacggaaatg ttgaatactc
    9421 atactcttcc tttttcaata ttattgaagc atttatcagg gttattgtct catgagcgga
    9481 tacatatttg aatgtattta gaaaaataaa caaatagggg ttccgcgcac atttccccga
    9541 aaagtgccac ctgacgtcga cggatcggga gatcaacttg tttattgcag cttataatgg
    9601 ttacaaataa agcaatagca tcacaaattt cacaaataaa gcattttttt cactgcattc
    9661 tagttgtggt ttgtccaaac tcatcaatgt atcttatcat gtctggatca actggataac
    9721 tcaagctaac caaaatcatc ccaaacttcc caccccatac cctattacca ctgccaatta
    9781 cctagtggtt tcatttactc taaacctgtg attcctctga attattttca ttttaaagaa
    9841 attgtatttg ttaaatatgt actacaaact tagtagtttt taaagaaatt gtatttgtta
    9901 aatatgtact acaaacttag tagt
//
```

Sequence of pLVX-PCP-tagRFP in GenBank format

```
LOCUS       pLVX_PCP_tagRFP        9933 bp ds-DNA     circular     30-DEC-2015
DEFINITION  Lentiviral vector for doxycycline-inducible expression of a gene in
            cells containing the Tet-On 3G transactivator protein.
ACCESSION   .
VERSION     .
KEYWORDS    pLVX-TRE3G
```

```
SOURCE      synthetic DNA construct
  ORGANISM  synthetic DNA construct
REFERENCE   1  (bases 1 to 7819)
  AUTHORS   Clontech
  TITLE     Direct Submission
  JOURNAL   Exported Friday, Jun 27, 2014 from SnapGene Viewer 2.4.2
            http://www.snapgene.com
COMMENT
COMMENT
COMMENT     ApEinfo:methylated:1
FEATURES             Location/Qualifiers
     misc_feature    3508..4683
                     /label=PCP-tagRFP
                     /ApEinfo_fwdcolor=cyan
                     /ApEinfo_revcolor=green
                     /ApEinfo_graphicformat=arrow_data {{0 1 2 0 0 -1} {} 0}
                     width 5 offset 0
     LTR             1..634
                     /note="5' LTR"
                     /note="5' long terminal repeat (LTR) from HIV-1"
                     /label=5' LTR
                     /ApEinfo_fwdcolor=pink
                     /ApEinfo_revcolor=pink
                     /ApEinfo_graphicformat=arrow_data {{0 1 2 0 0 -1} {} 0}
                     width 5 offset 0
     misc_feature    681..806
                     /note="HIV-1 psi"
                     /note="packaging signal of human immunodeficiency virus
                     type 1"
                     /label=HIV-1 psi
                     /ApEinfo_fwdcolor=pink
                     /ApEinfo_revcolor=pink
                     /ApEinfo_graphicformat=arrow_data {{0 1 2 0 0 -1} {} 0}
                     width 5 offset 0
     misc_feature    1303..1536
                     /note="RRE"
                     /note="The Rev response element (RRE) of HIV-1 allows for
                     Rev-dependent mRNA export from the nucleus to the
                     cytoplasm."
                     /label=RRE
                     /ApEinfo_fwdcolor=pink
                     /ApEinfo_revcolor=pink
                     /ApEinfo_graphicformat=arrow_data {{0 1 2 0 0 -1} {} 0}
                     width 5 offset 0
     misc_feature    2028..2144
                     /note="cPPT/CTS"
                     /note="central polypurine tract and central termination
                     sequence of HIV-1 (lacking the first T)"
                     /label=cPPT/CTS
                     /ApEinfo_fwdcolor=pink
                     /ApEinfo_revcolor=pink
                     /ApEinfo_graphicformat=arrow_data {{0 1 2 0 0 -1} {} 0}
                     width 5 offset 0
     misc_feature    3514..3537
                     /translation="MGPKKKRKVGYPYDVPDYAIEGRHMLASKTIVLSVGEATRTLTE
                     IQSTADRQIFEEKVGPLVGRLRLTASLRQNGAKTAYRVNLKLDQADVVDSGLPKVRYT
                     QVWSHDVTIVANSTEASRKSLYDLTKSLVATSQVEDLVVNLVPLGRADPLASKTIVLS
                     VGEATRTLTEIQSTADRQIFEEKVGPLVGRLRLTASLRQNGAKTAYRVNLKLDQADVV
                     DSGLPKVRYTQVWSHDVTIVANSTEASRKSLYDLTKSLVATSQVEDLVVNLVPLGRPP
                     VATMVSKGEELFTGVVPILVELDGDVNGHKFSVSGEGEGDATYGKLTLKFICTTGKLP
                     VPWPTLVTTLTYGVQCFSRYPDHMKQHDFFKSAMPEGYVQERTIFFKDDGNYKTRAEV
                     KFEGDTLVNRIELKGIDFKEDGNILGHKLEYNYNSHNVYIMADKQKNGIKVNFKIRHN
                     IEDGSVQLADHYQQNTPIGDGPVLLPDNHYLSTQSALSKDPNEKRDHMVLLEFVTAAG
                     ITLGMDELYK*"
                     /label=SV40 NLS
                     /ApEinfo_fwdcolor=pink
                     /ApEinfo_revcolor=pink
                     /ApEinfo_graphicformat=arrow_data {{0 1 2 0 0 -1} {} 0}
                     width 5 offset 0
     misc_feature    3538..3564
```

/translation="MGPKKKRKVGYPYDVPDYAIEGRHMLASKTIVLSVGEATRTLTE
IQSTADRQIFEEKVGPLVGRLRLTASLRQNGAKTAYRVNLKLDQADVVDSGLPKVRYT
QVWSHDVTIVANSTEASRKSLYDLTKSLVATSQVEDLVVNLVPLGRADPLASKTIVLS
VGEATRTLTEIQSTADRQIFEEKVGPLVGRLRLTASLRQNGAKTAYRVNLKLDQADVV
DSGLPKVRYTQVWSHDVTIVANSTEASRKSLYDLTKSLVATSQVEDLVVNLVPLGRPP
VATMVSKGEELFTGVVPILVELDGDVNGHKFSVSGEGEGDATYGKLTLKFICTTGKLP
VPWPTLVTTLTYGVQCFSRYPDHMKQHDFFKSAMPEGYVQERTIFFKDDGNYKTRAEV
KFEGDTLVNRIELKGIDFKEDGNILGHKLEYNYNSHNVYIMADKQKNGIKVNFKIRHN
IEDGSVQLADHYQQNTPIGDGPVLLPDNHYLSTQSALSKDPNEKRDHMVLLEFVTAAG
ITLGMDELYK*"
/label=HA
/ApEinfo_fwdcolor=pink
/ApEinfo_revcolor=pink
/ApEinfo_graphicformat=arrow_data {{0 1 2 0 0 -1} {} 0}
width 5 offset 0
promoter 4718..5217
/note="PGK promoter"
/note="mouse phosphoglycerate kinase 1 promoter"
/label=PGK promoter
/ApEinfo_fwdcolor=pink
/ApEinfo_revcolor=pink
/ApEinfo_graphicformat=arrow_data {{0 1 2 0 0 -1} {} 0}
width 5 offset 0
promoter 2209..3425
/label=hUbC_promoter
/ApEinfo_fwdcolor=pink
/ApEinfo_revcolor=pink
/ApEinfo_graphicformat=arrow_data {{0 1 2 0 0 -1} {} 0}
width 5 offset 0
CDS 5238..5837
/codon_start=1
/gene="pac from Streptomyces alboniger"
/product="puromycin N-acetyltransferase"
/note="PuroR"
/note="confers resistance to puromycin"
/translation="MTEYKPTVRLATRDDVPRAVRTLAAAFADYPATRHTVDPDRHIE
R
VTELQELFLTRVGLDIGKVWVADDGAAVAVWTTPESVEAGAVFAEIGPRMAELSGSRL
A
AQQQMEGLLAPHRPKEPAWFLATVGVSPDHQGKGLGSAVVLPGVEAAERAGVPAFLET
S APRNLPFYERLGFTVTADVEVPEGPRTWCMTRKPGA"
/label=puromycin N-acetyltransferase
/ApEinfo_fwdcolor=pink
/ApEinfo_revcolor=pink
/ApEinfo_graphicformat=arrow_data {{0 1 2 0 0 -1} {} 0}
width 5 offset 0
misc_feature 5855..6443
/note="WPRE"
/note="woodchuck hepatitis virus posttranscriptional
regulatory element"
/label=WPRE
/ApEinfo_fwdcolor=pink
/ApEinfo_revcolor=pink
/ApEinfo_graphicformat=arrow_data {{0 1 2 0 0 -1} {} 0}
width 5 offset 0
LTR 6651..7284
/note="3' LTR"
/note="3' long terminal repeat (LTR) from HIV-1"
/label=3' LTR
/ApEinfo_fwdcolor=pink
/ApEinfo_revcolor=pink
/ApEinfo_graphicformat=arrow_data {{0 1 2 0 0 -1} {} 0}
width 5 offset 0
rep_origin complement(7815..8400)
/direction=LEFT
/note="ori"
/note="high-copy-number ColE1/pMB1/pBR322/pUC origin of
replication"
/label=ori
/ApEinfo_fwdcolor=pink

/ApEinfo_revcolor=pink
/ApEinfo_graphicformat=arrow_data {{0 1 2 0 0 -1} {} 0}
width 5 offset 0
CDS complement(8571..9431)
/codon_start=1
/gene="bla"
/product="beta-lactamase"
/note="AmpR"
/note="confers resistance to ampicillin, carbenicillin,
and related antibiotics"
/translation="MSIQHFRVALIPFFAAFCLPVFAHPETLVKVKDAEDQLGARVGY
I
ELDLNSGKILESFRPEERFPMMSTFKVLLCGAVLSRIDAGQEQLGRRIHYSQNDLVEY
S
PVTEKHLTDGMTVRELCSAAITMSDNTAANLLLTTIGGPKELTAFLHNMGDHVTRLDR
W
EPELNEAIPNDERDTTMPVAMATTLRKLLTGELLTLASRQQLIDWMEADKVAGPLLRS
A
LPAGWFIADKSGAGERGSRGIIAALGPDGKPSRIVVIYTTGSQATMDERNRQIAEIGA
S LIKHW"
/label=beta-lactamase
/ApEinfo_fwdcolor=pink
/ApEinfo_revcolor=pink
/ApEinfo_graphicformat=arrow_data {{0 1 2 0 0 -1} {} 0}
width 5 offset 0
polyA_signal 9584..9718
/note="SV40 poly(A) signal"
/note="SV40 polyadenylation signal"
/label=SV40 poly(A) signal
/ApEinfo_fwdcolor=pink
/ApEinfo_revcolor=pink
/ApEinfo_graphicformat=arrow_data {{0 1 2 0 0 -1} {} 0}
width 5 offset 0
misc_feature 3970..4677
/label=TagRFP
/ApEinfo_fwdcolor=#ff0000
/ApEinfo_revcolor=green
/ApEinfo_graphicformat=arrow_data {{0 1 2 0 0 -1} {} 0}
width 5 offset 0
ORIGIN
1 tggaagggct aattcactcc caaagaagac aagatatcct tgatctgtgg atctaccaca
61 cacaaggcta cttccctgat tagcagaact acacaccagg gccaggggtc agatatccac
121 tgacctttgg atggtgctac aagctagtac cagttgagcc agataaggta gaagaggcca
181 ataaaggaga gaacaccagc ttgttacacc ctgtgagcct gcatgggatg gatgacccgg
241 agagagaagt gttagagtgg aggtttgaca gccgcctagc atttcatcac gtggcccgag
301 agctgcatcc ggagtacttc aagaactgct gatatcgagc ttgctacaag ggactttccg
361 ctggggactt tccagggagg cgtggcctgg gcgggactgg ggagtggcga gccctcagat
421 cctgcatata agcagctgct ttttgcctgt actgggtctc tctggttaga ccagatctga
481 gcctgggagc tctctggcta actagggaac ccactgctta agcctcaata aagcttgcct
541 tgagtgcttc aagtagtgtg tgcccgtctg ttgtgtgact ctggtaacta gagatccctc
601 agaccctttt agtcagtgtg gaaaatctct agcagtggcg cccgaacagg gacttgaaag
661 cgaaagggaa accagaggag ctctctcgac gcaggactcg gcttgctgaa gcgcgcacgg
721 caagaggcga ggggcggcga ctggtgagta cgccaaaaat tttgactagc ggaggctaga
781 aggagagaga tgggtgcgag agcgtcagta ttaagcgggg gagaattaga tcgcgatggg
841 aaaaaattcg gttaaggcca gggggaaaga aaaaatataa attaaaacat atagtatggg
901 caagcaggga gctagaacga ttcgcagtta atcctggcct gttagaaaca tcagaaggct
961 gtagacaaat actgggacag ctacaaccat cccttcagac aggatcagaa gaacttagat
1021 cattatataa tacagtagca accctctatt gtgtgcatca aaggatagag ataaaagaca
1081 ccaaggaagc tttagacaag atagaggaag agcaaaacaa aagtaagacc accgcacagc
1141 aagcggccgg ccgctgatct tcagacctgg aggaggagat atgagggaca attggagaag
1201 tgaattatat aaatataaag tagtaaaaat tgaaccatta ggagtagcac ccaccaaggc
1261 aaagagaaga gtggtgcaga gagaaaaaag agcagtggga ataggagctt tgttccttgg
1321 gttcttggga gcagcaggaa gcactatggg cgcagcgtca atgacgctga cggtacaggc
1381 cagacaatta ttgtctggta tagtgcagca gcagaacaat ttgctgaggg ctattgaggc
1441 gcaacagcat ctgttgcaac tcacagtctg gggcatcaag cagctccagg caagaatcct
1501 ggctgtggaa agatacctaa aggatcaaca gctcctgggg atttggggtt gctctggaaa
1561 actcatttgc accactgctg tgccttggaa tgctagttgg agtaataaat ctctggaaca
1621 gatttggaat cacacgacct ggatggagtg ggacagagaa attaacaatt acacaagctt
1681 aatacactcc ttaattgaag aatcgcaaaa ccagcaagaa aagaatgaac aagaattatt
1741 ggaattagat aaatgggcaa gtttgtggaa ttggtttaac ataacaaatt ggctgtggta

1801 tataaaatta ttcataatga tagtaggagg cttggtaggt ttaagaatag tttttgctgt
1861 actttctata gtgaatagag ttaggcaggg atattcacca ttatcgtttc agacccacct
1921 cccaaccccg aggggacccg acaggcccga aggaatagaa gaagaaggtg gagagagaga
1981 cagagacaga tccattcgat tagtgaacgg atctcgacgg tatcgccttt aaaagaaaag
2041 gggggattgg ggggtacagt gcaggggaaa gaatagtaga cataatagca acagacatac
2101 aaactaaaga actacaaaaa caaattacaa aaattcaaaa ttttcgggtt tattacaggg
2161 acagcagaga tccagtttat cgatgaggcc ctttcgtctt cactcgagGA TCTGGCCTCC
2221 GCGCCGGGTT TTGGCGCCTC CCGCGGGCGC CCCCCTCCTC ACGGCGAGCG CTGCCACGTC
2281 AGACGAAGGG CGCAGCGAGC GTCCTGATCC TTCCGCCCGG ACGCTCAGGA CAGCGGCCCG
2341 CTGCTCATAA GACTCGGCCT TAGAACCCCA GTATCAGCAG AAGGACATTT TAGGACGGGA
2401 CTTGGGTGAC TCTAGGGCAC TGGTTTTCTT TCCAGAGAGC GGAACAGGCG AGGAAAAGTA
2461 GTCCCTTCTC GGCGATTCTG CGGAGGGATC TCCGTGGGGC GGTGAACGCC GATGATTATA
2521 TAAGGACGCG CCGGGTGTGG CACAGCTAGT TCCGTCGCAG CCGGGATTTG GGTCGCGGTT
2581 CTTGTTTGTG GATCGCTGTG ATCGTCACTT GGTGAGTAGC GGGCTGCTGG GCTGGCCGGG
2641 GCTTTCGTGG CCGCCGGGCC GCTCGGTGGG ACGGAAGCGT GTGGAGAGAC CGCCAAGGGC
2701 TGTAGTCTGG GTCCGCGAGC AAGGTTGCCC TGAACTGGGG GTTGGGGGGA GCGCAGCAAA
2761 ATGGCGGCTG TTCCCGAGTC TTGAATGGAA GACGCTTGTG AGGCGGGCTG TGAGGTCGTT
2821 GAAACAAGGT GGGGGGCATG GTGGGCGGCA AGAACCCAAG GTCTTGAGGC CTTCGCTAAT
2881 GCGGGAAAGC TCTTATTCGG GTGAGATGGG CTGGGGCACC ATCTGGGGAC CCTGACGTGA
2941 AGTTTGTCAC TGACTGGAGA ACTCGGGTTT GTCGTCTGGT TGCGGGGGCG GCAGTTATGC
3001 GGTGCCGTTG GGCAGTGCAC CCGTACCTTT GGGAGCGCGC GCCTCGTCGT GTCGTGACGT
3061 CACCCGTTCT GTTGGCTTAT AATGCAGGGT GGGGCCACCT GCCGGTAGGT GTGCGGTAGG
3121 CTTTTCTCCG TCGCAGGACG CAGGGTTCGG GCCTAGGGTA GGCTCTCCTG AATCGACAGG
3181 CGCCGGACCT CTGGTGAGGG GAGGGATAAG TGAGGCGTCA GTTTCTTTGG TCGGTTTTAT
3241 GTACCTATCT TCTTAAGTAG CTGAAGCTCC GGTTTTGAAC TATGCGCTCG GGGTTGGCGA
3301 GTGTGTTTTG TGAAGTTTTT TAGGCACCTT TTGAAATGTA ATCATTTGGG TCAATATGTA
3361 ATTTTCAGTG TTAGACTAGT AAATTGTCCG CTAAATTCTG GCCGTTTTTG GCTTTTTTGT
3421 TAGACGAAGC TTGGGCTGCA GGTCGAGCTC AAGCTTCGAA TTCTGCAGTC GACGGTACCG
3481 CGGGCCCGGG ATCCACCGGT CGCCACCATG GGCCCAAAAA AGAAAAGAAA AGTTGGCTAC
3541 CCCTACGACG TGCCCGACTA CGCCATCGAA GGCCGCCATA TGCTAGCCTC CAAAACCATC
3601 GTTCTTTCGG TCGGCGAGGC TACTCGCACT CTGACTGAGA TCCAGTCCAC CGCAGACCGT
3661 CAGATCTTCG AAGAGAAGGT CGGGCCTCTG GTGGGTCGGC TGCGCCTCAC GGCTTCGCTC
3721 CGTCAAAACG GAGCCAAGAC CGCGTATCGC GTCAACCTAA AACTGGATCA GGCGGACGTC
3781 GTTGATTCCG GACTTCCGAA AGTGCGCTAC ACTCAGGTAT GGTCGCACGA CGTGACAATC
3841 GTTGCGAATA GCACCGAGGC CTCGCGCAAA TCGTTGTACG ATTTGACCAA GTCCCTCGTC
3901 GCGACCTCGC AGGTCGAAGA TCTTGTCGTC AACCTTGTGC CGCTGGGCCG TCCACCGGTC
3961 GCCACCATGG TGTCTAAGGG CGAAGAGCTG ATTAAGGAGA ACATGCACAT GAAGCTGTAC
4021 ATGGAGGGCA CCGTGAACAA CCACCACTTC AAGTGCACAT CCGAGGGCGA AGGCAAGCCC
4081 TACGAGGGCA CCCAGACCAT GAGAATCAAG GTGGTCGAGG GCGGCCCTCT CCCCTTCGCC
4141 TTCGACATCC TGGCTACCAG CTTCATGTAC GGCAGCAGAA CCTTCATCAA CCACACCCAG
4201 GGCATCCCCG ACTTCTTTAA GCAGTCCTTC CCTGAGGGCT TCACATGGGA GAGAGTCACC
4261 ACATACGAAG ACGGGGGCGT GCTGACCGCT ACCCAGGACA CCAGCCTCCA GGACGGCTGC
4321 CTCATCTACA ACGTCAAGAT CAGAGGGGTG AACTTCCCAT CCAACGGCCC TGTGATGCAG
4381 AAGAAAACAC TCGGCTGGGA GGCCAACACC GAGATGCTGT ACCCCGCTGA CGGCGGCCTG
4441 GAAGGCAGAA GCGACATGGC CCTGAAGCTC GTGGGCGGGG GCCACCTGAT CTGCAACTTC
4501 AAGACCACAT ACAGATCCAA GAAACCCGCT AAGAACCTCA AGATGCCCGG CGTCTACTAT
4561 GTGGACCACA GACTGGAAAG AATCAAGGAG GCCGACAAAG AGACCTACGT CGAGCAGCAC
4621 GAGGTGGCTG TGGCCAGATA CTGCGACCTC CCTAGCAAAC TGGGGCACAA ACTTAATTAC
4681 AAGTAAtaaA TAGTAATCAA TTACGGGgaa ttctaccggg taggggaggc gcttttccca
4741 aggcagtctg gagcatgcgc tttagcagcc ccgctgggca cttggcgcta cacaagtggc
4801 ctctggcctc gcacacattc cacatccacc ggtaggcgcc aaccggctcc gttctttggt
4861 ggccccttcg cgccaccttc tactcctccc ctagtcagga agttcccccc cgccccgcag
4921 ctcgcgtcgt gcaggacgtg acaaatggaa gtagcacgtc tcactagtct cgtgcagatg
4981 gacagcaccg ctgagcaatg gaagcgggta ggcctttggg gcagcggcca atagcagctt
5041 tgctccttcg ctttctgggc tcagaggctg ggaaggggtg ggtccggggg cgggctcagg
5101 ggcgggctca ggggcggggc gggcgcccga aggtcctccg gaggcccggc attctgcacg
5161 cttcaaaagc gcacgtctgc cgcgctgttc tcctcttcct catctccggg cctttcgacc
5221 tgcagcccaa gcttaccatg accgagtaca agcccacggt gcgcctcgcc acccgcgacg
5281 acgtccccag ggccgtacgc accctcgccg ccgcgttcgc cgactacccc gccacgcgcc
5341 acaccgtcga tccggaccgc cacatcgagc gggtcaccga gctgcaagaa ctcttcctca
5401 cgcgcgtcgg gctcgacatc ggcaaggtgt gggtcgcgga cgacggcgcc gcggtggcgg
5461 tctggaccac gccggagagc gtcgaagcgg gggcggtgtt cgccgagatc ggcccgcgca
5521 tggccgagtt gagcggttcc cggctggccg cgcagcaaca gatggaaggc ctcctggcgc
5581 cgcaccggcc caaggagccc gcgtggttcc tggccaccgt cggcgtctcg cccgaccacc
5641 agggcaaggg tctgggcagc gccgtcgtgc tccccggagt ggaggcggcc gagcgcgccg
5701 gggtgcccgc cttcctggag acctccgcgc cccgcaacct ccccttctac gagcggctcg
5761 gcttcaccgt caccgccgac gtcgaggtgc ccgaaggacc gcgcacctgg tgcatgaccc
5821 gcaagcccgg tgcctgacgg ggcgcgtctg gaacaatcaa cctctggatt acaaaatttg
5881 tgaaagattg actggtattc ttaactatgt tgctcctttt acgctatgtg gatacgctgc
5941 tttaatgcct ttgtatcatg ctattgcttc ccgtatggct ttcattttct cctccttgta

6001 taaatcctgg ttgctgtctc tttatgagga gttgtggccc gttgtcaggc aacgtggcgt
6061 ggtgtgcact gtgtttgctg acgcaacccc cactggttgg ggcattgcca ccacctgtca
6121 gctcctttcc gggactttcg ctttccccct ccctattgcc acggcggaac tcatcgccgc
6181 ctgccttgcc cgctgctgga caggggctcg gctgttgggc actgacaatt ccgtggtgtt
6241 gtcggggaag ctgacgtcct ttccatggct gctcgcctgt gttgccacct ggattctgcg
6301 cgggacgtcc ttctgctacg tcccttcggc cctcaatcca gcggaccttc cttcccgcgg
6361 cctgctgccg gctctgcggc ctcttccgcg tcttcgcctt cgccctcaga cgagtcggat
6421 ctccctttgg gccgcctccc cgcctggaat taattctgca gtcgagacct agaaaaacat
6481 ggagcaatca caagtagcaa tacagcagct accaatgctg attgtgcctg gctagaagca
6541 caagaggagg aggaggtggg tttttccagt cacacctcag gtacctttaa gaccaatgac
6601 ttacaaggca gctgtagatc ttagccactt tttaaaagaa aagaggggac tggaagggct
6661 aattcactcc caacgaagac aagatatcct tgatctgtgg atctaccaca cacaaggcta
6721 cttccctgat tagcagaact acacaccagg gccaggggtc agatatccac tgacctttgg
6781 atggtgctac aagctagtac cagttgagcc agataaggta gaagaggcca ataaaggaga
6841 gaacaccagc ttgttacacc ctgtgagcct gcatgggatg gatgacccgg agagagaagt
6901 gttagagtgg aggtttgaca gccgcctagc atttcatcac gtggcccgag agctgcatcc
6961 ggagtacttc aagaactgct gatatcgagc ttgctacaag ggactttccg ctggggactt
7021 tccagggagg cgtggcctgg gcgggactgg ggagtggcga gccctcagat cctgcatata
7081 agcagctgct ttttgcctgt actgggtctc tctggttaga ccagatctga gcctgggagc
7141 tctctggcta actagggaac ccactgctta agcctcaata aagcttgcct tgagtgcttc
7201 aagtagtgtg tgcccgtctg ttgtgtgact ctggtaacta gagatccctc agaccctttt
7261 agtcagtgtg gaaaatctct agcagtagta gttcatgtca tcttattatt cagtatttat
7321 aacttgcaaa gaaatgaata tcagagagtg agaggccttg acattgctag cgttttaccg
7381 tcgacctcta gctagagctt ggcgtaatca tggtcatagc tgtttcctgt gtgaaattgt
7441 tatccgctca caattccaca caacatacga gccggaagca taaagtgtaa agcctggggt
7501 gcctaatgag tgagctaact cacattaatt gcgttgcgct cactgcccgc tttccagtcg
7561 ggaaacctgt cgtgccagct gcattaatga atcggccaac gcgcggggag aggcggtttg
7621 cgtattgggc gctcttccgc ttcctcgctc actgactcgc tgcgctcggt cgttcggctg
7681 cggcgagcgg tatcagctca ctcaaaggcg gtaatacggt tatccacaga atcaggggat
7741 aacgcaggaa agaacatgtg agcaaaaggc cagcaaaagg ccaggaaccg taaaaaggcc
7801 gcgttgctgg cgtttttcca taggctccgc ccccctgacg agcatcacaa aaatcgacgc
7861 tcaagtcaga ggtggcgaaa cccgacagga ctataaagat accaggcgtt tccccctgga
7921 agctccctcg tgcgctctcc tgttccgacc ctgccgctta ccggatacct gtccgccttt
7981 ctcccttcgg gaagcgtggc gctttctcat agctcacgct gtaggtatct cagttcggtg
8041 taggtcgttc gctccaagct gggctgtgtg cacgaacccc ccgttcagcc cgaccgctgc
8101 gccttatccg gtaactatcg tcttgagtcc aacccggtaa gacacgactt atcgccactg
8161 gcagcagcca ctggtaacag gattagcaga gcgaggtatg taggcggtgc tacagagttc
8221 ttgaagtggt ggcctaacta cggctacact agaagaacag tatttggtat ctgcgctctg
8281 ctgaagccag ttaccttcgg aaaaagagtt ggtagctctt gatccggcaa acaaaccacc
8341 gctggtagcg gttttttgt ttgcaagcag cagattacgc gcagaaaaaa aggatctcaa
8401 gaagatcctt tgatcttttc tacggggtct gacgctcagt ggaacgaaaa ctcacgttaa
8461 gggattttgg tcatgagatt atcaaaaagg atcttcacct agatcctttt aaattaaaaa
8521 tgaagtttta aatcaatcta aagtatatat gagtaaactt ggtctgacag ttaccaatgc
8581 ttaatcagtg aggcacctat ctcagcgatc tgtctatttc gttcatccat agttgcctga
8641 ctccccgtcg tgtagataac tacgatacgg gagggcttac catctggccc cagtgctgca
8701 atgataccgc gagacccacg ctcaccggct ccagatttat cagcaataaa ccagccagcc
8761 ggaagggccg agcgcagaag tggtcctgca actttatccg cctccatcca gtctattaat
8821 tgttgccggg aagctagagt aagtagttcg ccagttaata gtttgcgcaa cgttgttgcc
8881 attgctacag gcatcgtggt gtcacgctcg tcgtttggta tggcttcatt cagctccggt
8941 tcccaacgat caaggcgagt tacatgatcc cccatgttgt gcaaaaaagc ggttagctcc
9001 ttcggtcctc cgatcgttgt cagaagtaag ttggccgcag tgttatcact catggttatg
9061 gcagcactgc ataattctct tactgtcatg ccatccgtaa gatgcttttc tgtgactggt
9121 gagtactcaa ccaagtcatt ctgagaatag tgtatgcggc gaccgagttg ctcttgcccg
9181 gcgtcaatac gggataatac cgcgccacat agcagaactt taaaagtgct catcattgga
9241 aaacgttctt cggggcgaaa actctcaagg atcttaccgc tgttgagatc cagttcgatg
9301 taacccactc gtgcacccaa ctgatcttca gcatctttta ctttcaccag cgtttctggg
9361 tgagcaaaaa caggaaggca aaatgccgca aaaaagggaa taagggcgac acggaaatgt
9421 tgaatactca tactcttcct ttttcaatat tattgaagca tttatcaggg ttattgtctc
9481 atgagcggat acatatttga atgtatttag aaaaataaac aaataggggt tccgcgcaca
9541 tttccccgaa aagtgccacc tgacgtcgac ggatcgggag atcaacttgt ttattgcagc
9601 ttataatggt tacaaataaa gcaatagcat cacaaatttc acaaataaag catttttttc
9661 actgcattct agttgtggtt tgtccaaact catcaatgta tcttatcatg tctggatcaa
9721 ctggataact caagctaacc aaaatcatcc caaacttccc accccatacc ctattaccac
9781 tgccaattac ctagtggttt catttactct aaacctgtga ttcctctgaa ttattttcat
9841 tttaaagaaa ttgtatttgt taaatatgta ctacaaactt agtagttttt aaagaaattg
9901 tatttgttaa atatgtacta caaacttagt agt
//

Sequence of psgRNA1.0-6xMS2-telo in GenBank format

```
LOCUS       psgRNA1_0_6xMS2        4117 bp ds-DNA     circular     02-JAN-2016
DEFINITION  pEYFP-N1
ACCESSION
KEYWORDS
  ORGANISM  other sequences; artificial sequences; vectors.
COMMENT
COMMENT     ApEinfo:methylated:1
FEATURES             Location/Qualifiers
     misc_feature    259..783
                     /label=sgRNA
                     /ApEinfo_fwdcolor=#ffff80
                     /ApEinfo_revcolor=green
                     /ApEinfo_graphicformat=arrow_data {{0 1 2 0 0 -1} {} 0}
                     width 5 offset 0
     misc_feature    386..407
                     /label=MS2SL
                     /ApEinfo_fwdcolor=#008000
                     /ApEinfo_revcolor=green
                     /ApEinfo_graphicformat=arrow_data {{0 1 2 0 0 -1} {} 0}
                     width 5 offset 0
     misc_feature    456..477
                     /label=MS2SL(1)
                     /ApEinfo_label=MS2SL
                     /ApEinfo_fwdcolor=#008000
                     /ApEinfo_revcolor=green
                     /ApEinfo_graphicformat=arrow_data {{0 1 2 0 0 -1} {} 0}
                     width 5 offset 0
     rep_origin      complement(1165..1471)
                     /label=f1_origin
                     /ApEinfo_fwdcolor=pink
                     /ApEinfo_revcolor=pink
                     /ApEinfo_graphicformat=arrow_data {{0 1 2 0 0 -1} {} 0}
                     width 5 offset 0
     misc_feature    666..687
                     /label=MS2SL(2)
                     /ApEinfo_label=MS2SL
                     /ApEinfo_fwdcolor=#008000
                     /ApEinfo_revcolor=green
                     /ApEinfo_graphicformat=arrow_data {{0 1 2 0 0 -1} {} 0}
                     width 5 offset 0
     promoter        1550..1578
                     /label=AmpR_promoter
                     /ApEinfo_fwdcolor=pink
                     /ApEinfo_revcolor=pink
                     /ApEinfo_graphicformat=arrow_data {{0 1 2 0 0 -1} {} 0}
                     width 5 offset 0
     misc_feature    596..617
                     /label=MS2SL(3)
                     /ApEinfo_label=MS2SL
                     /ApEinfo_fwdcolor=#008000
                     /ApEinfo_revcolor=green
                     /ApEinfo_graphicformat=arrow_data {{0 1 2 0 0 -1} {} 0}
                     width 5 offset 0
     misc_feature    complement(1650..1865)
                     /label=SV40_enhancer
                     /ApEinfo_fwdcolor=pink
                     /ApEinfo_revcolor=pink
                     /ApEinfo_graphicformat=arrow_data {{0 1 2 0 0 -1} {} 0}
                     width 5 offset 0
     promoter        10..258
                     /dnas_title="Human U6 Promoter"
                     /vntifkey="29"
                     /label=Human U6 Promoter
                     /ApEinfo_fwdcolor=pink
                     /ApEinfo_revcolor=pink
                     /ApEinfo_graphicformat=arrow_data {{0 1 2 0 0 -1} {} 0}
                     width 5 offset 0
```

```
     promoter        1662..1930
                     /label=SV40_promoter
                     /ApEinfo_fwdcolor=pink
                     /ApEinfo_revcolor=pink
                     /ApEinfo_graphicformat=arrow_data {{0 1 2 0 0 -1} {} 0}
                     width 5 offset 0
     rep_origin      1829..1906
                     /label=SV40_origin
                     /ApEinfo_fwdcolor=pink
                     /ApEinfo_revcolor=pink
                     /ApEinfo_graphicformat=arrow_data {{0 1 2 0 0 -1} {} 0}
                     width 5 offset 0
     CDS             2013..2807
                     /gene="NeoR/KanR"
                     /note="ORF frame 1"
                     /translation="MIEQDGLHAGSPAAWVERLFGYDWAQQTIGCSDAAVFRLSAQGR
                     PVLFVKTDLSGALNELQDEAARLSWLATTGVPCAAVLDVVTEAGRDWLLLGEVPGQDL
                     LSSHLAPAEKVSIMADAMRRLHTLDPATCPFDHQAKHRIERARTRMEAGLVDQDDLDE
                     EHQGLAPAELFARLKASMPDGEDLVVTHGDACLPNIMVENGRFSGFIDCGRLGVADRY
                     QDIALATRDIAEELGGEWADRFLVLYGIAAPDSQRIAFYRLLDEFF*"
                     /label=NeoR/KanR
                     /ApEinfo_fwdcolor=pink
                     /ApEinfo_revcolor=pink
                     /ApEinfo_graphicformat=arrow_data {{0 1 2 0 0 -1} {} 0}
                     width 5 offset 0
     terminator      2982..3251
                     /label=TK_PA_terminator
                     /ApEinfo_fwdcolor=pink
                     /ApEinfo_revcolor=pink
                     /ApEinfo_graphicformat=arrow_data {{0 1 2 0 0 -1} {} 0}
                     width 5 offset 0
     rep_origin      3399..4018
                     /label=pBR322_origin
                     /ApEinfo_fwdcolor=pink
                     /ApEinfo_revcolor=pink
                     /ApEinfo_graphicformat=arrow_data {{0 1 2 0 0 -1} {} 0}
                     width 5 offset 0
     misc_feature    526..547
                     /label=MS2SL(4)
                     /ApEinfo_label=MS2SL
                     /ApEinfo_fwdcolor=#008000
                     /ApEinfo_revcolor=green
                     /ApEinfo_graphicformat=arrow_data {{0 1 2 0 0 -1} {} 0}
                     width 5 offset 0
     misc_feature    736..757
                     /label=MS2SL(5)
                     /ApEinfo_label=MS2SL
                     /ApEinfo_fwdcolor=#008000
                     /ApEinfo_revcolor=green
                     /ApEinfo_graphicformat=arrow_data {{0 1 2 0 0 -1} {} 0}
                     width 5 offset 0
     misc_feature    259..280
                     /label=Telomere target sequence
                     /ApEinfo_fwdcolor=cyan
                     /ApEinfo_revcolor=green
                     /ApEinfo_graphicformat=arrow_data {{0 1 2 0 0 -1} {} 0}
                     width 5 offset 0
ORIGIN
        1 TAGTTATTAg agggcctatt tcccatgatt ccttcatatt tgcatatacg atacaaggct
       61 gttagagaga taattggaat taatttgact gtaaacacaa agatattagt acaaaatacg
      121 tgacgtagaa agtaataatt tcttgggtag tttgcagttt taaaattatg ttttaaaatg
      181 gactatcata tgcttaccgt aacttgaaag tatttcgatt tcttggcttt atatatcttG
      241 TGGAAAGGAC GAAACACCGT TAGGGTTAGG GTTAGGGTTA gtttAagagc taTGCTGgaa
      301 aCAGCAtagc aagttTaaat aaggctagtc cgttatcaac ttgaaaaagt ggcaccgagt
      361 cggtgcTCTG CAGGTCGACT CTAGAAAACA TGAGGATCAC CCATGTCTGC AGTATTCCCG
      421 GGTTCATTAG ATCCTAAGGT ACCTAATTGC CTAGAAAACA TGAGGATCAC CCATGTCCGA
      481 CACCGAACGT GCGACAAAGC TAAGTGCGCC GTACGGAGAT CCGATAAACA TGAGGATCAC
      541 CCATGTCCTT GCGAGATTAC GCGGCACATT ATTGGTCGAC GCCGCGAGCT ATCGTAAACA
      601 TGAGGATCAC CCATGTCTCT GTTTGACGCG CTGGACGCCC ATGATCGTCC GATCTGGCCC
      661 GCGTTAAACA TGAGGATCAC CCATGTCGCT CCGCGGCTTG AAACCATACA CCGAACGAGA
```

721 CGGTCAATCG CGCAGAAACA TGAGGATCAC CCATGTCGGA CCGAGGGCTC TAGAAATTTT
781 TTTGCGGCCG CGACTCTAGA TCATAATCAG CCATACCACA TTTGTAGAGG TTTTACTTGC
841 TTTAAAAAAC CTCCCACACC TCCCCCTGAA CCTGAAACAT AAAATGAATG CAATTGTTGT
901 TGTTAACTTG TTTATTGCAG CTTATAATGG TTACAAATAA AGCAATAGCA TCACAAATTT
961 CACAAATAAA GCATTTTTTT CACTGCATTC TAGTTGTGGT TTGTCCAAAC TCATCAATGT
1021 ATCTTAAGGC GTAAATTGTA AGCGTTAATA TTTTGTTAAA ATTCGCGTTA AATTTTTGTT
1081 AAATCAGCTC ATTTTTTAAC CAATAGGCCG AAATCGGCAA AATCCCTTAT AAATCAAAAG
1141 AATAGACCGA GATAGGGTTG AGTGTTGTTC CAGTTTGGAA CAAGAGTCCA CTATTAAAGA
1201 ACGTGGACTC CAACGTCAAA GGGCGAAAAA CCGTCTATCA GGGCGATGGC CCACTACGTG
1261 AACCATCACC CTAATCAAGT TTTTTGGGGT CGAGGTGCCG TAAAGCACTA AATCGGAACC
1321 CTAAAGGGAG CCCCCGATTT AGAGCTTGAC GGGGAAAGCC GGCGAACGTG GCGAGAAAGG
1381 AAGGGAAGAA AGCGAAAGGA GCGGGCGCTA GGGCGCTGGC AAGTGTAGCG GTCACGCTGC
1441 GCGTAACCAC CACACCCGCC GCGCTTAATG CGCCGCTACA GGGCGCGTCA GGTGGCACTT
1501 TTCGGGGAAA TGTGCGCGGA ACCCCTATTT GTTTATTTTT CTAAATACAT TCAAATATGT
1561 ATCCGCTCAT GAGACAATAA CCCTGATAAA TGCTTCAATA ATATTGAAAA AGGAAGAGTC
1621 CTGAGGCGGA AAGAACCAGC TGTGGAATGT GTGTCAGTTA GGGTGTGGAA AGTCCCCAGG
1681 CTCCCCAGCA GGCAGAAGTA TGCAAAGCAT GCATCTCAAT TAGTCAGCAA CCAGGTGTGG
1741 AAAGTCCCCA GGCTCCCCAG CAGGCAGAAG TATGCAAAGC ATGCATCTCA ATTAGTCAGC
1801 AACCATAGTC CCGCCCCTAA CTCCGCCCAT CCCGCCCCTA ACTCCGCCCA GTTCCGCCCA
1861 TTCTCCGCCC CATGGCTGAC TAATTTTTTT TATTTATGCA GAGGCCGAGG CCGCCTCGGC
1921 CTCTGAGCTA TTCCAGAAGT AGTGAGGAGG CTTTTTTGGA GGCCTAGGCT TTTGCAAAGA
1981 TCGATCAAGA GACAGGATGA GGATCGTTTC GCATGATTGA ACAAGATGGA TTGCACGCAG
2041 GTTCTCCGGC CGCTTGGGTG GAGAGGCTAT TCGGCTATGA CTGGGCACAA CAGACAATCG
2101 GCTGCTCTGA TGCCGCCGTG TTCCGGCTGT CAGCGCAGGG GCGCCCGGTT CTTTTTGTCA
2161 AGACCGACCT GTCCGGTGCC CTGAATGAAC TGCAAGACGA GGCAGCGCGG CTATCGTGGC
2221 TGGCCACGAC GGGCGTTCCT TGCGCAGCTG TGCTCGACGT TGTCACTGAA GCGGGAAGGG
2281 ACTGGCTGCT ATTGGGCGAA GTGCCGGGGC AGGATCTCCT GTCATCTCAC CTTGCTCCTG
2341 CCGAGAAAGT ATCCATCATG GCTGATGCAA TGCGGCGGCT GCATACGCTT GATCCGGCTA
2401 CCTGCCCATT CGACCACCAA GCGAAACATC GCATCGAGCG AGCACGTACT CGGATGGAAG
2461 CCGGTCTTGT CGATCAGGAT GATCTGGACG AAGAGCATCA GGGGCTCGCG CCAGCCGAAC
2521 TGTTCGCCAG GCTCAAGGCG AGCATGCCCG ACGGCGAGGA TCTCGTCGTG ACCCATGGCG
2581 ATGCCTGCTT GCCGAATATC ATGGTGGAAA ATGGCCGCTT TTCTGGATTC ATCGACTGTG
2641 GCCGGCTGGG TGTGGCGGAC CGCTATCAGG ACATAGCGTT GGCTACCCGT GATATTGCTG
2701 AAGAGCTTGG CGGCGAATGG GCTGACCGCT TCCTCGTGCT TTACGGTATC GCCGCTCCCG
2761 ATTCGCAGCG CATCGCCTTC TATCGCCTTC TTGACGAGTT CTTCTGAGCG GGACTCTGGG
2821 GTTCGAAATG ACCGACCAAG CGACGCCCAA CCTGCCATCA CGAGATTTCG ATTCCACCGC
2881 CGCCTTCTAT GAAAGGTTGG GCTTCGGAAT CGTTTTCCGG GACGCCGGCT GGATGATCCT
2941 CCAGCGCGGG GATCTCATGC TGGAGTTCTT CGCCCACCCT AGGGGGAGGC TAACTGAAAC
3001 ACGGAAGGAG ACAATACCGG AAGGAACCCG CGCTATGACG GCAATAAAAA GACAGAATAA
3061 AACGCACGGT GTTGGGTCGT TTGTTCATAA ACGCGGGGTT CGGTCCCAGG GCTGGCACTC
3121 TGTCGATACC CCACCGAGAC CCCATTGGGG CCAATACGCC CGCGTTTCTT CCTTTTCCCC
3181 ACCCCACCCC CCAAGTTCGG GTGAAGGCCC AGGGCTCGCA GCCAACGTCG GGGCGGCAGG
3241 CCCTGCCATA GCCTCAGGTT ACTCATATAT ACTTTAGATT GATTTAAAAC TTCATTTTTA
3301 ATTTAAAAGG ATCTAGGTGA AGATCCTTTT TGATAATCTC ATGACCAAAA TCCCTTAACG
3361 TGAGTTTTCG TTCCACTGAG CGTCAGACCC CGTAGAAAAG ATCAAAGGAT CTTCTTGAGA
3421 TCCTTTTTTT CTGCGCGTAA TCTGCTGCTT GCAAACAAAA AAACCACCGC TACCAGCGGT
3481 GGTTTGTTTG CCGGATCAAG AGCTACCAAC TCTTTTTCCG AAGGTAACTG GCTTCAGCAG
3541 AGCGCAGATA CCAAATACTG TCCTTCTAGT GTAGCCGTAG TTAGGCCACC ACTTCAAGAA
3601 CTCTGTAGCA CCGCCTACAT ACCTCGCTCT GCTAATCCTG TTACCAGTGG CTGCTGCCAG
3661 TGGCGATAAG TCGTGTCTTA CCGGGTTGGA CTCAAGACGA TAGTTACCGG ATAAGGCGCA
3721 GCGGTCGGGC TGAACGGGGG GTTCGTGCAC ACAGCCCAGC TTGGAGCGAA CGACCTACAC
3781 CGAACTGAGA TACCTACAGC GTGAGCTATG AGAAAGCGCC ACGCTTCCCG AAGGGAGAAA
3841 GGCGGACAGG TATCCGGTAA GCGGCAGGGT CGGAACAGGA GAGCGCACGA GGGAGCTTCC
3901 AGGGGGAAAC GCCTGGTATC TTTATAGTCC TGTCGGGTTT CGCCACCTCT GACTTGAGCG
3961 TCGATTTTTG TGATGCTCGT CAGGGGGGCG GAGCCTATGG AAAAACGCCA GCAACGCGGC
4021 CTTTTTACGG TTCCTGGCCT TTTGCTGGCC TTTTGCTCAC ATGTTCTTTC CTGCGTTATC
4081 CCCTGATTCT GTGGATAACC GTATTACCGC CATGCAT
//

Sequence of psgRNA1.0-6xPP7-telo in GenBank format

```
LOCUS       psgRNA1_0_6xPP7        4135 bp ds-DNA     circular     02-JAN-2016
DEFINITION  pEYFP-N1
ACCESSION
KEYWORDS
  ORGANISM  other sequences; artificial sequences; vectors.
COMMENT
COMMENT     ApEinfo:methylated:1
FEATURES             Location/Qualifiers
     misc_feature    259..801
                     /label=sgRNA
                     /ApEinfo_fwdcolor=#ffff80
                     /ApEinfo_revcolor=green
                     /ApEinfo_graphicformat=arrow_data {{0 1 2 0 0 -1} {} 0}
                     width 5 offset 0
     misc_feature    386..410
                     /label=PP7
                     /ApEinfo_fwdcolor=cyan
                     /ApEinfo_revcolor=green
                     /ApEinfo_graphicformat=arrow_data {{0 1 2 0 0 -1} {} 0}
                     width 5 offset 0
     rep_origin      complement(1183..1489)
                     /label=f1_origin
                     /ApEinfo_fwdcolor=pink
                     /ApEinfo_revcolor=pink
                     /ApEinfo_graphicformat=arrow_data {{0 1 2 0 0 -1} {} 0}
                     width 5 offset 0
     misc_feature    678..702
                     /label=PP7(1)
                     /ApEinfo_label=PP7
                     /ApEinfo_fwdcolor=cyan
                     /ApEinfo_revcolor=green
                     /ApEinfo_graphicformat=arrow_data {{0 1 2 0 0 -1} {} 0}
                     width 5 offset 0
     promoter        1568..1596
                     /label=AmpR_promoter
                     /ApEinfo_fwdcolor=pink
                     /ApEinfo_revcolor=pink
                     /ApEinfo_graphicformat=arrow_data {{0 1 2 0 0 -1} {} 0}
                     width 5 offset 0
     misc_feature    605..629
                     /label=PP7(2)
                     /ApEinfo_label=PP7
                     /ApEinfo_fwdcolor=cyan
                     /ApEinfo_revcolor=green
                     /ApEinfo_graphicformat=arrow_data {{0 1 2 0 0 -1} {} 0}
                     width 5 offset 0
     misc_feature    751..775
                     /label=PP7(3)
                     /ApEinfo_label=PP7
                     /ApEinfo_fwdcolor=cyan
                     /ApEinfo_revcolor=green
                     /ApEinfo_graphicformat=arrow_data {{0 1 2 0 0 -1} {} 0}
                     width 5 offset 0
     misc_feature    complement(1668..1883)
                     /label=SV40_enhancer
                     /ApEinfo_fwdcolor=pink
                     /ApEinfo_revcolor=pink
                     /ApEinfo_graphicformat=arrow_data {{0 1 2 0 0 -1} {} 0}
                     width 5 offset 0
     promoter        10..258
                     /dnas_title="Human U6 Promoter"
                     /vntifkey="29"
                     /label=Human U6 Promoter
                     /ApEinfo_fwdcolor=pink
                     /ApEinfo_revcolor=pink
                     /ApEinfo_graphicformat=arrow_data {{0 1 2 0 0 -1} {} 0}
                     width 5 offset 0
```

```
     promoter        1680..1948
                     /label=SV40_promoter
                     /ApEinfo_fwdcolor=pink
                     /ApEinfo_revcolor=pink
                     /ApEinfo_graphicformat=arrow_data {{0 1 2 0 0 -1} {} 0}
                     width 5 offset 0
     rep_origin      1847..1924
                     /label=SV40_origin
                     /ApEinfo_fwdcolor=pink
                     /ApEinfo_revcolor=pink
                     /ApEinfo_graphicformat=arrow_data {{0 1 2 0 0 -1} {} 0}
                     width 5 offset 0
     CDS             2031..2825
                     /gene="NeoR/KanR"
                     /note="ORF frame 1"
                     /translation="MIEQDGLHAGSPAAWVERLFGYDWAQQTIGCSDAAVFRLSAQGR
                     PVLFVKTDLSGALNELQDEAARLSWLATTGVPCAAVLDVVTEAGRDWLLLGEVPGQDL
                     LSSHLAPAEKVSIMADAMRRLHTLDPATCPFDHQAKHRIERARTRMEAGLVDQDDLDE
                     EHQGLAPAELFARLKASMPDGEDLVVTHGDACLPNIMVENGRFSGFIDCGRLGVADRY
                     QDIALATRDIAEELGGEWADRFLVLYGIAAPDSQRIAFYRLLDEFF*"
                     /label=NeoR/KanR
                     /ApEinfo_fwdcolor=pink
                     /ApEinfo_revcolor=pink
                     /ApEinfo_graphicformat=arrow_data {{0 1 2 0 0 -1} {} 0}
                     width 5 offset 0
     terminator      3000..3269
                     /label=TK_PA_terminator
                     /ApEinfo_fwdcolor=pink
                     /ApEinfo_revcolor=pink
                     /ApEinfo_graphicformat=arrow_data {{0 1 2 0 0 -1} {} 0}
                     width 5 offset 0
     rep_origin      3417..4036
                     /label=pBR322_origin
                     /ApEinfo_fwdcolor=pink
                     /ApEinfo_revcolor=pink
                     /ApEinfo_graphicformat=arrow_data {{0 1 2 0 0 -1} {} 0}
                     width 5 offset 0
     misc_feature    532..556
                     /label=PP7(4)
                     /ApEinfo_label=PP7
                     /ApEinfo_fwdcolor=cyan
                     /ApEinfo_revcolor=green
                     /ApEinfo_graphicformat=arrow_data {{0 1 2 0 0 -1} {} 0}
                     width 5 offset 0
     misc_feature    459..483
                     /label=PP7(5)
                     /ApEinfo_label=PP7
                     /ApEinfo_fwdcolor=cyan
                     /ApEinfo_revcolor=green
                     /ApEinfo_graphicformat=arrow_data {{0 1 2 0 0 -1} {} 0}
                     width 5 offset 0
     misc_feature    259..280
                     /label=Telomere target sequence
                     /ApEinfo_fwdcolor=#008000
                     /ApEinfo_revcolor=green
                     /ApEinfo_graphicformat=arrow_data {{0 1 2 0 0 -1} {} 0}
                     width 5 offset 0
ORIGIN
        1 TAGTTATTAg agggcctatt tcccatgatt ccttcatatt tgcatatacg atacaaggct
       61 gttagagaga taattggaat taatttgact gtaaacacaa agatattagt acaaaatacg
      121 tgacgtagaa agtaataatt tcttgggtag tttgcagttt taaaattatg ttttaaaatg
      181 gactatcata tgcttaccgt aacttgaaag tatttcgatt tcttggcttt atatatcttG
      241 TGGAAAGGAC GAAACACCGT TAGGGTTAGG GTTAGGGTTA gtttAagagc taTGCTGgaa
      301 aCAGCAtagc aagttTaaat aaggctagtc cgttatcaac ttgaaaaagt ggcaccgagt
      361 cggtgcTCTG CAGGTCGACT CTAGAGGAGC AGACGATATG GCGTCGCTCC TGCAGTATTC
      421 CCGGGTTCAT TAGATCCTAA GGTACCTAAT TGCCTAGACC AGCAGAGCAT ATGGGCTCGC
      481 TGGCGACACC GAACGTGCGA CAAAGCTAAG TGCGCCGTAC GGAGATCCGA TGGAGCAGAC
      541 GATATGGCGT CGCTCCCTTG CGAGATTACG CGGCACATTA TTGGTCGACG CCGCGAGCTA
      601 TCGTCCAGCA GAGCATATGG GCTCGCTGGT CTGTTTGACG CGCTGGACGC CCATGATCGT
      661 CCGATCTGGC CCGCGTTGGA GCAGACGATA TGGCGTCGCT CCGCTCCGCG GCTTGAAACC
```

721 ATACACCGAA CGAGACGGTC AATCGCGCAG CCAGCAGAGC ATATGGGCTC GCTGGGGACC
781 GAGGGCTCTA GAAATTTTTT TGCGGCCGCG ACTCTAGATC ATAATCAGCC ATACCACATT
841 TGTAGAGGTT TTACTTGCTT TAAAAAACCT CCCACACCTC CCCCTGAACC TGAAACATAA
901 AATGAATGCA ATTGTTGTTG TTAACTTGTT TATTGCAGCT TATAATGGTT ACAAATAAAG
961 CAATAGCATC ACAAATTTCA CAAATAAAGC ATTTTTTTCA CTGCATTCTA GTTGTGGTTT
1021 GTCCAAACTC ATCAATGTAT CTTAAGGCGT AAATTGTAAG CGTTAATATT TTGTTAAAAT
1081 TCGCGTTAAA TTTTTGTTAA ATCAGCTCAT TTTTTAACCA ATAGGCCGAA ATCGGCAAAA
1141 TCCCTTATAA ATCAAAAGAA TAGACCGAGA TAGGGTTGAG TGTTGTTCCA GTTTGGAACA
1201 AGAGTCCACT ATTAAAGAAC GTGGACTCCA ACGTCAAAGG GCGAAAAACC GTCTATCAGG
1261 GCGATGGCCC ACTACGTGAA CCATCACCCT AATCAAGTTT TTTGGGGTCG AGGTGCCGTA
1321 AAGCACTAAA TCGGAACCCT AAAGGGAGCC CCCGATTTAG AGCTTGACGG GGAAAGCCGG
1381 CGAACGTGGC GAGAAAGGAA GGGAAGAAAG CGAAAGGAGC GGGCGCTAGG GCGCTGGCAA
1441 GTGTAGCGGT CACGCTGCGC GTAACCACCA CACCCGCCGC GCTTAATGCG CCGCTACAGG
1501 GCGCGTCAGG TGGCACTTTT CGGGGAAATG TGCGCGGAAC CCCTATTTGT TTATTTTTCT
1561 AAATACATTC AAATATGTAT CCGCTCATGA GACAATAACC CTGATAAATG CTTCAATAAT
1621 ATTGAAAAAG GAAGAGTCCT GAGGCGGAAA GAACCAGCTG TGGAATGTGT GTCAGTTAGG
1681 GTGTGGAAAG TCCCCAGGCT CCCCAGCAGG CAGAAGTATG CAAAGCATGC ATCTCAATTA
1741 GTCAGCAACC AGGTGTGGAA AGTCCCCAGG CTCCCCAGCA GGCAGAAGTA TGCAAAGCAT
1801 GCATCTCAAT TAGTCAGCAA CCATAGTCCC GCCCCTAACT CCGCCCATCC CGCCCCTAAC
1861 TCCGCCCAGT TCCGCCCATT CTCCGCCCCA TGGCTGACTA ATTTTTTTTA TTTATGCAGA
1921 GGCCGAGGCC GCCTCGGCCT CTGAGCTATT CCAGAAGTAG TGAGGAGGCT TTTTTGGAGG
1981 CCTAGGCTTT TGCAAAGATC GATCAAGAGA CAGGATGAGG ATCGTTTCGC ATGATTGAAC
2041 AAGATGGATT GCACGCAGGT TCTCCGGCCG CTTGGGTGGA GAGGCTATTC GGCTATGACT
2101 GGGCACAACA GACAATCGGC TGCTCTGATG CCGCCGTGTT CCGGCTGTCA GCGCAGGGGC
2161 GCCCGGTTCT TTTTGTCAAG ACCGACCTGT CCGGTGCCCT GAATGAACTG CAAGACGAGG
2221 CAGCGCGGCT ATCGTGGCTG GCCACGACGG GCGTTCCTTG CGCAGCTGTG CTCGACGTTG
2281 TCACTGAAGC GGGAAGGGAC TGGCTGCTAT TGGGCGAAGT GCCGGGGCAG GATCTCCTGT
2341 CATCTCACCT TGCTCCTGCC GAGAAAGTAT CCATCATGGC TGATGCAATG CGGCGGCTGC
2401 ATACGCTTGA TCCGGCTACC TGCCCATTCG ACCACCAAGC GAAACATCGC ATCGAGCGAG
2461 CACGTACTCG GATGGAAGCC GGTCTTGTCG ATCAGGATGA TCTGGACGAA GAGCATCAGG
2521 GGCTCGCGCC AGCCGAACTG TTCGCCAGGC TCAAGGCGAG CATGCCCGAC GGCGAGGATC
2581 TCGTCGTGAC CCATGGCGAT GCCTGCTTGC CGAATATCAT GGTGGAAAAT GGCCGCTTTT
2641 CTGGATTCAT CGACTGTGGC CGGCTGGGTG TGGCGGACCG CTATCAGGAC ATAGCGTTGG
2701 CTACCCGTGA TATTGCTGAA GAGCTTGGCG GCGAATGGGC TGACCGCTTC CTCGTGCTTT
2761 ACGGTATCGC CGCTCCCGAT TCGCAGCGCA TCGCCTTCTA TCGCCTTCTT GACGAGTTCT
2821 TCTGAGCGGG ACTCTGGGGT TCGAAATGAC CGACCAAGCG ACGCCCAACC TGCCATCACG
2881 AGATTTCGAT TCCACCGCCG CCTTCTATGA AAGGTTGGGC TTCGGAATCG TTTTCCGGGA
2941 CGCCGGCTGG ATGATCCTCC AGCGCGGGGA TCTCATGCTG GAGTTCTTCG CCCACCCTAG
3001 GGGGAGGCTA ACTGAAACAC GGAAGGAGAC AATACCGGAA GGAACCCGCG CTATGACGGC
3061 AATAAAAAGA CAGAATAAAA CGCACGGTGT TGGGTCGTTT GTTCATAAAC GCGGGGTTCG
3121 GTCCCAGGGC TGGCACTCTG TCGATACCCC ACCGAGACCC CATTGGGGCC AATACGCCCG
3181 CGTTTCTTCC TTTTCCCCAC CCCACCCCCC AAGTTCGGGT GAAGGCCCAG GGCTCGCAGC
3241 CAACGTCGGG GCGGCAGGCC CTGCCATAGC CTCAGGTTAC TCATATATAC TTTAGATTGA
3301 TTTAAAACTT CATTTTTAAT TTAAAAGGAT CTAGGTGAAG ATCCTTTTTG ATAATCTCAT
3361 GACCAAAATC CCTTAACGTG AGTTTTCGTT CCACTGAGCG TCAGACCCCG TAGAAAAGAT
3421 CAAAGGATCT TCTTGAGATC CTTTTTTTCT GCGCGTAATC TGCTGCTTGC AAACAAAAAA
3481 ACCACCGCTA CCAGCGGTGG TTTGTTTGCC GGATCAAGAG CTACCAACTC TTTTTCCGAA
3541 GGTAACTGGC TTCAGCAGAG CGCAGATACC AAATACTGTC CTTCTAGTGT AGCCGTAGTT
3601 AGGCCACCAC TTCAAGAACT CTGTAGCACC GCCTACATAC CTCGCTCTGC TAATCCTGTT
3661 ACCAGTGGCT GCTGCCAGTG GCGATAAGTC GTGTCTTACC GGGTTGGACT CAAGACGATA
3721 GTTACCGGAT AAGGCGCAGC GGTCGGGCTG AACGGGGGGT TCGTGCACAC AGCCCAGCTT
3781 GGAGCGAACG ACCTACACCG AACTGAGATA CCTACAGCGT GAGCTATGAG AAAGCGCCAC
3841 GCTTCCCGAA GGGAGAAAGG CGGACAGGTA TCCGGTAAGC GGCAGGGTCG GAACAGGAGA
3901 GCGCACGAGG GAGCTTCCAG GGGGAAACGC CTGGTATCTT TATAGTCCTG TCGGGTTTCG
3961 CCACCTCTGA CTTGAGCGTC GATTTTTGTG ATGCTCGTCA GGGGGGCGGA GCCTATGGAA
4021 AAACGCCAGC AACGCGGCCT TTTTACGGTT CCTGGCCTTT TGCTGGCCTT TTGCTCACAT
4081 GTTCTTTCCT GCGTTATCCC CTGATTCTGT GGATAACCGT ATTACCGCCA TGCAT
//

Sequence of psgRNA2.0-6xMS2-telo in GenBank format

```
LOCUS       psgRNA2_0_6xMS2         4027 bp ds-DNA     circular     02-JAN-2016
DEFINITION  pEYFP-N1
ACCESSION
KEYWORDS
  ORGANISM  other sequences; artificial sequences; vectors.
COMMENT
COMMENT     ApEinfo:methylated:1
FEATURES             Location/Qualifiers
     misc_feature    259..693
                     /label=sgRNA
                     /ApEinfo_fwdcolor=#ffff80
                     /ApEinfo_revcolor=green
                     /ApEinfo_graphicformat=arrow_data {{0 1 2 0 0 -1} {} 0}
                     width 5 offset 0
     misc_feature    436..457
                     /label=MS2SL
                     /ApEinfo_fwdcolor=#008000
                     /ApEinfo_revcolor=green
                     /ApEinfo_graphicformat=arrow_data {{0 1 2 0 0 -1} {} 0}
                     width 5 offset 0
     misc_feature    506..527
                     /label=MS2SL(1)
                     /ApEinfo_label=MS2SL
                     /ApEinfo_fwdcolor=#008000
                     /ApEinfo_revcolor=green
                     /ApEinfo_graphicformat=arrow_data {{0 1 2 0 0 -1} {} 0}
                     width 5 offset 0
     rep_origin      complement(1075..1381)
                     /label=f1_origin
                     /ApEinfo_fwdcolor=pink
                     /ApEinfo_revcolor=pink
                     /ApEinfo_graphicformat=arrow_data {{0 1 2 0 0 -1} {} 0}
                     width 5 offset 0
     promoter        1460..1488
                     /label=AmpR_promoter
                     /ApEinfo_fwdcolor=pink
                     /ApEinfo_revcolor=pink
                     /ApEinfo_graphicformat=arrow_data {{0 1 2 0 0 -1} {} 0}
                     width 5 offset 0
     misc_feature    646..667
                     /label=MS2SL(2)
                     /ApEinfo_label=MS2SL
                     /ApEinfo_fwdcolor=#008000
                     /ApEinfo_revcolor=green
                     /ApEinfo_graphicformat=arrow_data {{0 1 2 0 0 -1} {} 0}
                     width 5 offset 0
     misc_feature    complement(1560..1775)
                     /label=SV40_enhancer
                     /ApEinfo_fwdcolor=pink
                     /ApEinfo_revcolor=pink
                     /ApEinfo_graphicformat=arrow_data {{0 1 2 0 0 -1} {} 0}
                     width 5 offset 0
     promoter        10..258
                     /dnas_title="Human U6 Promoter"
                     /vntifkey="29"
                     /label=Human U6 Promoter
                     /ApEinfo_fwdcolor=pink
                     /ApEinfo_revcolor=pink
                     /ApEinfo_graphicformat=arrow_data {{0 1 2 0 0 -1} {} 0}
                     width 5 offset 0
     promoter        1572..1840
                     /label=SV40_promoter
                     /ApEinfo_fwdcolor=pink
                     /ApEinfo_revcolor=pink
                     /ApEinfo_graphicformat=arrow_data {{0 1 2 0 0 -1} {} 0}
                     width 5 offset 0
     rep_origin      1739..1816
```

```
                    /label=SV40_origin
                    /ApEinfo_fwdcolor=pink
                    /ApEinfo_revcolor=pink
                    /ApEinfo_graphicformat=arrow_data {{0 1 2 0 0 -1} {} 0}
                    width 5 offset 0
     CDS            1923..2717
                    /gene="NeoR/KanR"
                    /note="ORF frame 1"
                    /translation="MIEQDGLHAGSPAAWVERLFGYDWAQQTIGCSDAAVFRLSAQGR
                    PVLFVKTDLSGALNELQDEAARLSWLATTGVPCAAVLDVVTEAGRDWLLLGEVPGQDL
                    LSSHLAPAEKVSIMADAMRRLHTLDPATCPFDHQAKHRIERARTRMEAGLVDQDDLDE
                    EHQGLAPAELFARLKASMPDGEDLVVTHGDACLPNIMVENGRFSGFIDCGRLGVADRY
                    QDIALATRDIAEELGGEWADRFLVLYGIAAPDSQRIAFYRLLDEFF*"
                    /label=NeoR/KanR
                    /ApEinfo_fwdcolor=pink
                    /ApEinfo_revcolor=pink
                    /ApEinfo_graphicformat=arrow_data {{0 1 2 0 0 -1} {} 0}
                    width 5 offset 0
     terminator     2892..3161
                    /label=TK_PA_terminator
                    /ApEinfo_fwdcolor=pink
                    /ApEinfo_revcolor=pink
                    /ApEinfo_graphicformat=arrow_data {{0 1 2 0 0 -1} {} 0}
                    width 5 offset 0
     rep_origin     3309..3928
                    /label=pBR322_origin
                    /ApEinfo_fwdcolor=pink
                    /ApEinfo_revcolor=pink
                    /ApEinfo_graphicformat=arrow_data {{0 1 2 0 0 -1} {} 0}
                    width 5 offset 0
     misc_feature   576..597
                    /label=MS2SL(3)
                    /ApEinfo_label=MS2SL
                    /ApEinfo_fwdcolor=#008000
                    /ApEinfo_revcolor=green
                    /ApEinfo_graphicformat=arrow_data {{0 1 2 0 0 -1} {} 0}
                    width 5 offset 0
     misc_feature   293..326
                    /label=MS2SL(4)
                    /ApEinfo_label=MS2SL
                    /ApEinfo_fwdcolor=#008000
                    /ApEinfo_revcolor=green
                    /ApEinfo_graphicformat=arrow_data {{0 1 2 0 0 -1} {} 0}
                    width 5 offset 0
     misc_feature   363..396
                    /label=MS2SL(5)
                    /ApEinfo_label=MS2SL
                    /ApEinfo_fwdcolor=#008000
                    /ApEinfo_revcolor=green
                    /ApEinfo_graphicformat=arrow_data {{0 1 2 0 0 -1} {} 0}
                    width 5 offset 0
     misc_feature   259..280
                    /label=Telomere target sequence
                    /ApEinfo_fwdcolor=cyan
                    /ApEinfo_revcolor=green
                    /ApEinfo_graphicformat=arrow_data {{0 1 2 0 0 -1} {} 0}
                    width 5 offset 0
ORIGIN
        1 TAGTTATTAg agggcctatt tcccatgatt ccttcatatt tgcatatacg atacaaggct
       61 gttagagaga taattggaat taatttgact gtaaacacaa agatattagt acaaaatacg
      121 tgacgtagaa agtaataatt tcttgggtag tttgcagttt taaaattatg ttttaaaatg
      181 gactatcata tgcttaccgt aacttgaaag tatttcgatt tcttggcttt atatatcttG
      241 TGGAAAGGAC GAAACACCGT TAGGGTTAGG GTTAGGGTTA gtttAagagc taGGCCAACA
      301 TGAGGATCAC CCATGTCTGC AGGGCCtagc aagttTaaat aaggctagtc cgttatcaac
      361 ttGGCCAACA TGAGGATCAC CCATGTCTGC AGGGCCaagt ggcaccgagt cggtgcTCTG
      421 CAGGTCGACT CTAGAAAACA TGAGGATCAC CCATGTCTGC AGTATTCCCG GGTTCATTAG
      481 ATCCTAAGGT ACCTAATTGC CTAGAAAACA TGAGGATCAC CCATGTCCGA CACCGAACGT
      541 GCGACAAAGC TAAGTGCGCC GTACGGAGAT CCGATAAACA TGAGGATCAC CCATGTCCTT
      601 GCGAGATTAC GCGGCACATT ATTGGTCGAC GCCGCGAGCT ATCGTAAACA TGAGGATCAC
      661 CCATGTCGGA CCGAGGGCTC TAGAAATTTT TTTGCGGCCG CGACTCTAGA TCATAATCAG
```

```
 721 CCATACCACA TTTGTAGAGG TTTTACTTGC TTTAAAAAAC CTCCCACACC TCCCCCTGAA
 781 CCTGAAACAT AAAATGAATG CAATTGTTGT TGTTAACTTG TTTATTGCAG CTTATAATGG
 841 TTACAAATAA AGCAATAGCA TCACAAATTT CACAAATAAA GCATTTTTTT CACTGCATTC
 901 TAGTTGTGGT TTGTCCAAAC TCATCAATGT ATCTTAAGGC GTAAATTGTA AGCGTTAATA
 961 TTTTGTTAAA ATTCGCGTTA AATTTTTGTT AAATCAGCTC ATTTTTTAAC CAATAGGCCG
1021 AAATCGGCAA AATCCCTTAT AAATCAAAAG AATAGACCGA GATAGGGTTG AGTGTTGTTC
1081 CAGTTTGGAA CAAGAGTCCA CTATTAAAGA ACGTGGACTC CAACGTCAAA GGGCGAAAAA
1141 CCGTCTATCA GGGCGATGGC CCACTACGTG AACCATCACC CTAATCAAGT TTTTTGGGGT
1201 CGAGGTGCCG TAAAGCACTA AATCGGAACC CTAAAGGGAG CCCCCGATTT AGAGCTTGAC
1261 GGGGAAAGCC GGCGAACGTG GCGAGAAAGG AAGGGAAGAA AGCGAAAGGA GCGGGCGCTA
1321 GGGCGCTGGC AAGTGTAGCG GTCACGCTGC GCGTAACCAC CACACCCGCC GCGCTTAATG
1381 CGCCGCTACA GGGCGCGTCA GGTGGCACTT TTCGGGGAAA TGTGCGCGGA ACCCCTATTT
1441 GTTTATTTTT CTAAATACAT TCAAATATGT ATCCGCTCAT GAGACAATAA CCCTGATAAA
1501 TGCTTCAATA ATATTGAAAA AGGAAGAGTC CTGAGGCGGA AAGAACCAGC TGTGGAATGT
1561 GTGTCAGTTA GGGTGTGGAA AGTCCCCAGG CTCCCCAGCA GGCAGAAGTA TGCAAAGCAT
1621 GCATCTCAAT TAGTCAGCAA CCAGGTGTGG AAAGTCCCCA GGCTCCCCAG CAGGCAGAAG
1681 TATGCAAAGC ATGCATCTCA ATTAGTCAGC AACCATAGTC CCGCCCCTAA CTCCGCCCAT
1741 CCCGCCCCTA ACTCCGCCCA GTTCCGCCCA TTCTCCGCCC CATGGCTGAC TAATTTTTTT
1801 TATTTATGCA GAGGCCGAGG CCGCCTCGGC CTCTGAGCTA TTCCAGAAGT AGTGAGGAGG
1861 CTTTTTTGGA GGCCTAGGCT TTTGCAAAGA TCGATCAAGA GACAGGATGA GGATCGTTTC
1921 GCATGATTGA ACAAGATGGA TTGCACGCAG GTTCTCCGGC CGCTTGGGTG GAGAGGCTAT
1981 TCGGCTATGA CTGGGCACAA CAGACAATCG GCTGCTCTGA TGCCGCCGTG TTCCGGCTGT
2041 CAGCGCAGGG GCGCCCGGTT CTTTTTGTCA AGACCGACCT GTCCGGTGCC CTGAATGAAC
2101 TGCAAGACGA GGCAGCGCGG CTATCGTGGC TGGCCACGAC GGGCGTTCCT TGCGCAGCTG
2161 TGCTCGACGT TGTCACTGAA GCGGGAAGGG ACTGGCTGCT ATTGGGCGAA GTGCCGGGGC
2221 AGGATCTCCT GTCATCTCAC CTTGCTCCTG CCGAGAAAGT ATCCATCATG GCTGATGCAA
2281 TGCGGCGGCT GCATACGCTT GATCCGGCTA CCTGCCCATT CGACCACCAA GCGAAACATC
2341 GCATCGAGCG AGCACGTACT CGGATGGAAG CCGGTCTTGT CGATCAGGAT GATCTGGACG
2401 AAGAGCATCA GGGGCTCGCG CCAGCCGAAC TGTTCGCCAG GCTCAAGGCG AGCATGCCCG
2461 ACGGCGAGGA TCTCGTCGTG ACCCATGGCG ATGCCTGCTT GCCGAATATC ATGGTGGAAA
2521 ATGGCCGCTT TTCTGGATTC ATCGACTGTG GCCGGCTGGG TGTGGCGGAC CGCTATCAGG
2581 ACATAGCGTT GGCTACCCGT GATATTGCTG AAGAGCTTGG CGGCGAATGG GCTGACCGCT
2641 TCCTCGTGCT TTACGGTATC GCCGCTCCCG ATTCGCAGCG CATCGCCTTC TATCGCCTTC
2701 TTGACGAGTT CTTCTGAGCG GGACTCTGGG GTTCGAAATG ACCGACCAAG CGACGCCCAA
2761 CCTGCCATCA CGAGATTTCG ATTCCACCGC CGCCTTCTAT GAAAGGTTGG GCTTCGGAAT
2821 CGTTTTCCGG GACGCCGGCT GGATGATCCT CCAGCGCGGG GATCTCATGC TGGAGTTCTT
2881 CGCCCACCCT AGGGGGAGGC TAACTGAAAC ACGGAAGGAG ACAATACCGG AAGGAACCCG
2941 CGCTATGACG GCAATAAAAA GACAGAATAA AACGCACGGT GTTGGGTCGT TTGTTCATAA
3001 ACGCGGGGTT CGGTCCCAGG GCTGGCACTC TGTCGATACC CCACCGAGAC CCCATTGGGG
3061 CCAATACGCC CGCGTTTCTT CCTTTTCCCC ACCCCACCCC CCAAGTTCGG GTGAAGGCCC
3121 AGGGCTCGCA GCCAACGTCG GGGCGGCAGG CCCTGCCATA GCCTCAGGTT ACTCATATAT
3181 ACTTTAGATT GATTTAAAAC TTCATTTTTA ATTTAAAAGG ATCTAGGTGA AGATCCTTTT
3241 TGATAATCTC ATGACCAAAA TCCCTTAACG TGAGTTTTCG TTCCACTGAG CGTCAGACCC
3301 CGTAGAAAAG ATCAAAGGAT CTTCTTGAGA TCCTTTTTTT CTGCGCGTAA TCTGCTGCTT
3361 GCAAACAAAA AAACCACCGC TACCAGCGGT GGTTTGTTTG CCGGATCAAG AGCTACCAAC
3421 TCTTTTTCCG AAGGTAACTG GCTTCAGCAG AGCGCAGATA CCAAATACTG TCCTTCTAGT
3481 GTAGCCGTAG TTAGGCCACC ACTTCAAGAA CTCTGTAGCA CCGCCTACAT ACCTCGCTCT
3541 GCTAATCCTG TTACCAGTGG CTGCTGCCAG TGGCGATAAG TCGTGTCTTA CCGGGTTGGA
3601 CTCAAGACGA TAGTTACCGG ATAAGGCGCA GCGGTCGGGC TGAACGGGGG GTTCGTGCAC
3661 ACAGCCCAGC TTGGAGCGAA CGACCTACAC CGAACTGAGA TACCTACAGC GTGAGCTATG
3721 AGAAAGCGCC ACGCTTCCCG AAGGGAGAAA GGCGGACAGG TATCCGGTAA GCGGCAGGGT
3781 CGGAACAGGA GAGCGCACGA GGGAGCTTCC AGGGGGAAAC GCCTGGTATC TTTATAGTCC
3841 TGTCGGGTTT CGCCACCTCT GACTTGAGCG TCGATTTTTG TGATGCTCGT CAGGGGGGCG
3901 GAGCCTATGG AAAAACGCCA GCAACGCGGC CTTTTTACGG TTCCTGGCCT TTTGCTGGCC
3961 TTTTGCTCAC ATGTTCTTTC CTGCGTTATC CCCTGATTCT GTGGATAACC GTATTACCGC
4021 CATGCAT
//
```

Sequence of psgRNA2.0-6xPP7-telo in GenBank format

```
LOCUS       psgRNA2_0_6xPP7        4021 bp ds-DNA     circular     02-JAN-2016
DEFINITION  pEYFP-N1
ACCESSION
KEYWORDS
  ORGANISM  other sequences; artificial sequences; vectors.
COMMENT
COMMENT
COMMENT     ApEinfo:methylated:1
FEATURES             Location/Qualifiers
     misc_feature    259..687
                     /label=sgRNA
                     /ApEinfo_fwdcolor=#ffff80
                     /ApEinfo_revcolor=green
                     /ApEinfo_graphicformat=arrow_data {{0 1 2 0 0 -1} {} 0}
                     width 5 offset 0
     misc_feature    418..442
                     /label=PP7
                     /ApEinfo_fwdcolor=cyan
                     /ApEinfo_revcolor=green
                     /ApEinfo_graphicformat=arrow_data {{0 1 2 0 0 -1} {} 0}
                     width 5 offset 0
     misc_feature    293..317
                     /label=PP7(1)
                     /ApEinfo_label=PP7
                     /ApEinfo_fwdcolor=cyan
                     /ApEinfo_revcolor=green
                     /ApEinfo_graphicformat=arrow_data {{0 1 2 0 0 -1} {} 0}
                     width 5 offset 0
     rep_origin      complement(1069..1375)
                     /label=f1_origin
                     /ApEinfo_fwdcolor=pink
                     /ApEinfo_revcolor=pink
                     /ApEinfo_graphicformat=arrow_data {{0 1 2 0 0 -1} {} 0}
                     width 5 offset 0
     promoter        1454..1482
                     /label=AmpR_promoter
                     /ApEinfo_fwdcolor=pink
                     /ApEinfo_revcolor=pink
                     /ApEinfo_graphicformat=arrow_data {{0 1 2 0 0 -1} {} 0}
                     width 5 offset 0
     misc_feature    637..661
                     /label=PP7(2)
                     /ApEinfo_label=PP7
                     /ApEinfo_fwdcolor=cyan
                     /ApEinfo_revcolor=green
                     /ApEinfo_graphicformat=arrow_data {{0 1 2 0 0 -1} {} 0}
                     width 5 offset 0
     misc_feature    complement(1554..1769)
                     /label=SV40_enhancer
                     /ApEinfo_fwdcolor=pink
                     /ApEinfo_revcolor=pink
                     /ApEinfo_graphicformat=arrow_data {{0 1 2 0 0 -1} {} 0}
                     width 5 offset 0
     promoter        10..258
                     /dnas_title="Human U6 Promoter"
                     /vntifkey="29"
                     /label=Human U6 Promoter
                     /ApEinfo_fwdcolor=pink
                     /ApEinfo_revcolor=pink
                     /ApEinfo_graphicformat=arrow_data {{0 1 2 0 0 -1} {} 0}
                     width 5 offset 0
     promoter        1566..1834
                     /label=SV40_promoter
                     /ApEinfo_fwdcolor=pink
                     /ApEinfo_revcolor=pink
                     /ApEinfo_graphicformat=arrow_data {{0 1 2 0 0 -1} {} 0}
                     width 5 offset 0
```

```
     rep_origin      1733..1810
                     /label=SV40_origin
                     /ApEinfo_fwdcolor=pink
                     /ApEinfo_revcolor=pink
                     /ApEinfo_graphicformat=arrow_data {{0 1 2 0 0 -1} {} 0}
                     width 5 offset 0
     CDS             1917..2711
                     /gene="NeoR/KanR"
                     /note="ORF frame 1"
                     /translation="MIEQDGLHAGSPAAWVERLFGYDWAQQTIGCSDAAVFRLSAQGR
                     PVLFVKTDLSGALNELQDEAARLSWLATTGVPCAAVLDVVTEAGRDWLLLGEVPGQDL
                     LSSHLAPAEKVSIMADAMRRLHTLDPATCPFDHQAKHRIERARTRMEAGLVDQDDLDE
                     EHQGLAPAELFARLKASMPDGEDLVVTHGDACLPNIMVENGRFSGFIDCGRLGVADRY
                     QDIALATRDIAEELGGEWADRFLVLYGIAAPDSQRIAFYRLLDEFF*"
                     /label=NeoR/KanR
                     /ApEinfo_fwdcolor=pink
                     /ApEinfo_revcolor=pink
                     /ApEinfo_graphicformat=arrow_data {{0 1 2 0 0 -1} {} 0}
                     width 5 offset 0
     terminator      2886..3155
                     /label=TK_PA_terminator
                     /ApEinfo_fwdcolor=pink
                     /ApEinfo_revcolor=pink
                     /ApEinfo_graphicformat=arrow_data {{0 1 2 0 0 -1} {} 0}
                     width 5 offset 0
     rep_origin      3303..3922
                     /label=pBR322_origin
                     /ApEinfo_fwdcolor=pink
                     /ApEinfo_revcolor=pink
                     /ApEinfo_graphicformat=arrow_data {{0 1 2 0 0 -1} {} 0}
                     width 5 offset 0
     misc_feature    564..588
                     /label=PP7(3)
                     /ApEinfo_label=PP7
                     /ApEinfo_fwdcolor=cyan
                     /ApEinfo_revcolor=green
                     /ApEinfo_graphicformat=arrow_data {{0 1 2 0 0 -1} {} 0}
                     width 5 offset 0
     misc_feature    491..515
                     /label=PP7(4)
                     /ApEinfo_label=PP7
                     /ApEinfo_fwdcolor=cyan
                     /ApEinfo_revcolor=green
                     /ApEinfo_graphicformat=arrow_data {{0 1 2 0 0 -1} {} 0}
                     width 5 offset 0
     misc_feature    354..378
                     /label=PP7(5)
                     /ApEinfo_label=PP7
                     /ApEinfo_fwdcolor=cyan
                     /ApEinfo_revcolor=green
                     /ApEinfo_graphicformat=arrow_data {{0 1 2 0 0 -1} {} 0}
                     width 5 offset 0
     misc_feature    259..280
                     /label=telomere target sequence
                     /ApEinfo_fwdcolor=#008000
                     /ApEinfo_revcolor=green
                     /ApEinfo_graphicformat=arrow_data {{0 1 2 0 0 -1} {} 0}
                     width 5 offset 0
ORIGIN
        1 TAGTTATTAg agggcctatt tcccatgatt ccttcatatt tgcatatacg atacaaggct
       61 gttagagaga taattggaat taatttgact gtaaacacaa agatattagt acaaaatacg
      121 tgacgtagaa agtaataatt tcttgggtag tttgcagttt taaaattatg ttttaaaatg
      181 gactatcata tgcttaccgt aacttgaaag tatttcgatt tcttggcttt atatatcttG
      241 TGGAAAGGAC GAAACACCGT TAGGGTTAGG GTTAGGGTTA gtttAagagc taGGAGCAGA
      301 CGATATGGCG TCGCTCCtag caagttTaaa taaggctagt ccgttatcaa cttCCAGCAG
      361 AGCATATGGG CTCGCTGGaa gtggcaccga gtcggtgcTC TGCAGGTCGA CTCTAGAGGA
      421 GCAGACGATA TGGCGTCGCT CCTGCAGTAT TCCCGGGTTC ATTAGATCCT AAGGTACCTA
      481 ATTGCCTAGA CCAGCAGAGC ATATGGGCTC GCTGGCGACA CCGAACGTGC GACAAAGCTA
      541 AGTGCGCCGT ACGGAGATCC GATGGAGCAG ACGATATGGC GTCGCTCCCT TGCGAGATTA
      601 CGCGGCACAT TATTGGTCGA CGCCGCGAGC TATCGTCCAG CAGAGCATAT GGGCTCGCTG
```

661 GGGACCGAGG GCTCTAGAAA TTTTTTTGCG GCCGCGACTC TAGATCATAA TCAGCCATAC
721 CACATTTGTA GAGGTTTTAC TTGCTTTAAA AAACCTCCCA CACCTCCCCC TGAACCTGAA
781 ACATAAAATG AATGCAATTG TTGTTGTTAA CTTGTTTATT GCAGCTTATA ATGGTTACAA
841 ATAAAGCAAT AGCATCACAA ATTTCACAAA TAAAGCATTT TTTTCACTGC ATTCTAGTTG
901 TGGTTTGTCC AAACTCATCA ATGTATCTTA AGGCGTAAAT TGTAAGCGTT AATATTTTGT
961 TAAAATTCGC GTTAAATTTT TGTTAAATCA GCTCATTTTT TAACCAATAG GCCGAAATCG
1021 GCAAAATCCC TTATAAATCA AAAGAATAGA CCGAGATAGG GTTGAGTGTT GTTCCAGTTT
1081 GGAACAAGAG TCCACTATTA AAGAACGTGG ACTCCAACGT CAAAGGGCGA AAAACCGTCT
1141 ATCAGGGCGA TGGCCCACTA CGTGAACCAT CACCCTAATC AAGTTTTTTG GGGTCGAGGT
1201 GCCGTAAAGC ACTAAATCGG AACCCTAAAG GGAGCCCCCG ATTTAGAGCT TGACGGGGAA
1261 AGCCGGCGAA CGTGGCGAGA AAGGAAGGGA AGAAAGCGAA AGGAGCGGGC GCTAGGGCGC
1321 TGGCAAGTGT AGCGGTCACG CTGCGCGTAA CCACCACACC CGCCGCGCTT AATGCGCCGC
1381 TACAGGGCGC GTCAGGTGGC ACTTTTCGGG GAAATGTGCG CGGAACCCCT ATTTGTTTAT
1441 TTTTCTAAAT ACATTCAAAT ATGTATCCGC TCATGAGACA ATAACCCTGA TAAATGCTTC
1501 AATAATATTG AAAAAGGAAG AGTCCTGAGG CGGAAAGAAC CAGCTGTGGA ATGTGTGTCA
1561 GTTAGGGTGT GGAAAGTCCC CAGGCTCCCC AGCAGGCAGA AGTATGCAAA GCATGCATCT
1621 CAATTAGTCA GCAACCAGGT GTGGAAAGTC CCCAGGCTCC CCAGCAGGCA GAAGTATGCA
1681 AAGCATGCAT CTCAATTAGT CAGCAACCAT AGTCCCGCCC CTAACTCCGC CCATCCCGCC
1741 CCTAACTCCG CCCAGTTCCG CCCATTCTCC GCCCCATGGC TGACTAATTT TTTTTATTTA
1801 TGCAGAGGCC GAGGCCGCCT CGGCCTCTGA GCTATTCCAG AAGTAGTGAG GAGGCTTTTT
1861 TGGAGGCCTA GGCTTTTGCA AAGATCGATC AAGAGACAGG ATGAGGATCG TTTCGCATGA
1921 TTGAACAAGA TGGATTGCAC GCAGGTTCTC CGGCCGCTTG GGTGGAGAGG CTATTCGGCT
1981 ATGACTGGGC ACAACAGACA ATCGGCTGCT CTGATGCCGC CGTGTTCCGG CTGTCAGCGC
2041 AGGGGCGCCC GGTTCTTTTT GTCAAGACCG ACCTGTCCGG TGCCCTGAAT GAACTGCAAG
2101 ACGAGGCAGC GCGGCTATCG TGGCTGGCCA CGACGGGCGT TCCTTGCGCA GCTGTGCTCG
2161 ACGTTGTCAC TGAAGCGGGA AGGGACTGGC TGCTATTGGG CGAAGTGCCG GGGCAGGATC
2221 TCCTGTCATC TCACCTTGCT CCTGCCGAGA AAGTATCCAT CATGGCTGAT GCAATGCGGC
2281 GGCTGCATAC GCTTGATCCG GCTACCTGCC CATTCGACCA CCAAGCGAAA CATCGCATCG
2341 AGCGAGCACG TACTCGGATG GAAGCCGGTC TTGTCGATCA GGATGATCTG GACGAAGAGC
2401 ATCAGGGGCT CGCGCCAGCC GAACTGTTCG CCAGGCTCAA GGCGAGCATG CCCGACGGCG
2461 AGGATCTCGT CGTGACCCAT GGCGATGCCT GCTTGCCGAA TATCATGGTG GAAAATGGCC
2521 GCTTTTCTGG ATTCATCGAC TGTGGCCGGC TGGGTGTGGC GGACCGCTAT CAGGACATAG
2581 CGTTGGCTAC CCGTGATATT GCTGAAGAGC TTGGCGGCGA ATGGGCTGAC CGCTTCCTCG
2641 TGCTTTACGG TATCGCCGCT CCCGATTCGC AGCGCATCGC CTTCTATCGC CTTCTTGACG
2701 AGTTCTTCTG AGCGGGACTC TGGGGTTCGA AATGACCGAC CAAGCGACGC CCAACCTGCC
2761 ATCACGAGAT TTCGATTCCA CCGCCGCCTT CTATGAAAGG TTGGGCTTCG GAATCGTTTT
2821 CCGGGACGCC GGCTGGATGA TCCTCCAGCG CGGGGATCTC ATGCTGGAGT TCTTCGCCCA
2881 CCCTAGGGGG AGGCTAACTG AAACACGGAA GGAGACAATA CCGGAAGGAA CCCGCGCTAT
2941 GACGGCAATA AAAAGACAGA ATAAAACGCA CGGTGTTGGG TCGTTTGTTC ATAAACGCGG
3001 GGTTCGGTCC CAGGGCTGGC ACTCTGTCGA TACCCCACCG AGACCCCATT GGGGCCAATA
3061 CGCCCGCGTT TCTTCCTTTT CCCCACCCCA CCCCCCAAGT TCGGGTGAAG GCCCAGGGCT
3121 CGCAGCCAAC GTCGGGGCGG CAGGCCCTGC CATAGCCTCA GGTTACTCAT ATATACTTTA
3181 GATTGATTTA AAACTTCATT TTTAATTTAA AAGGATCTAG GTGAAGATCC TTTTTGATAA
3241 TCTCATGACC AAAATCCCTT AACGTGAGTT TTCGTTCCAC TGAGCGTCAG ACCCCGTAGA
3301 AAAGATCAAA GGATCTTCTT GAGATCCTTT TTTTCTGCGC GTAATCTGCT GCTTGCAAAC
3361 AAAAAAACCA CCGCTACCAG CGGTGGTTTG TTTGCCGGAT CAAGAGCTAC CAACTCTTTT
3421 TCCGAAGGTA ACTGGCTTCA GCAGAGCGCA GATACCAAAT ACTGTCCTTC TAGTGTAGCC
3481 GTAGTTAGGC CACCACTTCA AGAACTCTGT AGCACCGCCT ACATACCTCG CTCTGCTAAT
3541 CCTGTTACCA GTGGCTGCTG CCAGTGGCGA TAAGTCGTGT CTTACCGGGT TGGACTCAAG
3601 ACGATAGTTA CCGGATAAGG CGCAGCGGTC GGGCTGAACG GGGGGTTCGT GCACACAGCC
3661 CAGCTTGGAG CGAACGACCT ACACCGAACT GAGATACCTA CAGCGTGAGC TATGAGAAAG
3721 CGCCACGCTT CCCGAAGGGA GAAAGGCGGA CAGGTATCCG GTAAGCGGCA GGGTCGGAAC
3781 AGGAGAGCGC ACGAGGGAGC TTCCAGGGGG AAACGCCTGG TATCTTTATA GTCCTGTCGG
3841 GTTTCGCCAC CTCTGACTTG AGCGTCGATT TTTGTGATGC TCGTCAGGGG GGCGGAGCCT
3901 ATGGAAAAAC GCCAGCAACG CGGCCTTTTT ACGGTTCCTG GCCTTTTGCT GGCCTTTTGC
3961 TCACATGTTC TTTCCTGCGT TATCCCCTGA TTCTGTGGAT AACCGTATTA CCGCCATGCA
4021 T
//

Sequence of psgRNA2.0-6xPP7-cent in GenBank format

```
LOCUS       psgRNA2_0_6xPP7        4018 bp ds-DNA     circular     10-MAR-2016
DEFINITION  pEYFP-N1
ACCESSION
KEYWORDS
  ORGANISM  other sequences; artificial sequences; vectors.
COMMENT
COMMENT     ApEinfo:methylated:1
FEATURES             Location/Qualifiers
     misc_feature    join(259..259,278..684)
                     /label=sgRNA
                     /ApEinfo_fwdcolor=#ffff80
                     /ApEinfo_revcolor=green
                     /ApEinfo_graphicformat=arrow_data {{0 1 2 0 0 -1} {} 0}
                     width 5 offset 0
     misc_feature    415..439
                     /label=PP7
                     /ApEinfo_fwdcolor=cyan
                     /ApEinfo_revcolor=green
                     /ApEinfo_graphicformat=arrow_data {{0 1 2 0 0 -1} {} 0}
                     width 5 offset 0
     misc_feature    290..314
                     /label=PP7(1)
                     /ApEinfo_label=PP7
                     /ApEinfo_fwdcolor=cyan
                     /ApEinfo_revcolor=green
                     /ApEinfo_graphicformat=arrow_data {{0 1 2 0 0 -1} {} 0}
                     width 5 offset 0
     rep_origin      complement(1066..1372)
                     /label=f1_origin
                     /ApEinfo_fwdcolor=pink
                     /ApEinfo_revcolor=pink
                     /ApEinfo_graphicformat=arrow_data {{0 1 2 0 0 -1} {} 0}
                     width 5 offset 0
     promoter        1451..1479
                     /label=AmpR_promoter
                     /ApEinfo_fwdcolor=pink
                     /ApEinfo_revcolor=pink
                     /ApEinfo_graphicformat=arrow_data {{0 1 2 0 0 -1} {} 0}
                     width 5 offset 0
     misc_feature    634..658
                     /label=PP7(2)
                     /ApEinfo_label=PP7
                     /ApEinfo_fwdcolor=cyan
                     /ApEinfo_revcolor=green
                     /ApEinfo_graphicformat=arrow_data {{0 1 2 0 0 -1} {} 0}
                     width 5 offset 0
     misc_feature    complement(1551..1766)
                     /label=SV40_enhancer
                     /ApEinfo_fwdcolor=pink
                     /ApEinfo_revcolor=pink
                     /ApEinfo_graphicformat=arrow_data {{0 1 2 0 0 -1} {} 0}
                     width 5 offset 0
     promoter        10..258
                     /dnas_title="Human U6 Promoter"
                     /vntifkey="29"
                     /label=Human U6 Promoter
                     /ApEinfo_fwdcolor=pink
                     /ApEinfo_revcolor=pink
                     /ApEinfo_graphicformat=arrow_data {{0 1 2 0 0 -1} {} 0}
                     width 5 offset 0
     promoter        1563..1831
                     /label=SV40_promoter
                     /ApEinfo_fwdcolor=pink
                     /ApEinfo_revcolor=pink
                     /ApEinfo_graphicformat=arrow_data {{0 1 2 0 0 -1} {} 0}
                     width 5 offset 0
     rep_origin      1730..1807
```

```
                     /label=SV40_origin
                     /ApEinfo_fwdcolor=pink
                     /ApEinfo_revcolor=pink
                     /ApEinfo_graphicformat=arrow_data {{0 1 2 0 0 -1} {} 0}
                     width 5 offset 0
     CDS             1914..2708
                     /gene="NeoR/KanR"
                     /note="ORF frame 1"
                     /translation="MIEQDGLHAGSPAAWVERLFGYDWAQQTIGCSDAAVFRLSAQGR
                     PVLFVKTDLSGALNELQDEAARLSWLATTGVPCAAVLDVVTEAGRDWLLLGEVPGQDL
                     LSSHLAPAEKVSIMADAMRRLHTLDPATCPFDHQAKHRIERARTRMEAGLVDQDDLDE
                     EHQGLAPAELFARLKASMPDGEDLVVTHGDACLPNIMVENGRFSGFIDCGRLGVADRY
                     QDIALATRDIAEELGGEWADRFLVLYGIAAPDSQRIAFYRLLDEFF*"
                     /label=NeoR/KanR
                     /ApEinfo_fwdcolor=pink
                     /ApEinfo_revcolor=pink
                     /ApEinfo_graphicformat=arrow_data {{0 1 2 0 0 -1} {} 0}
                     width 5 offset 0
     terminator      2883..3152
                     /label=TK_PA_terminator
                     /ApEinfo_fwdcolor=pink
                     /ApEinfo_revcolor=pink
                     /ApEinfo_graphicformat=arrow_data {{0 1 2 0 0 -1} {} 0}
                     width 5 offset 0
     rep_origin      3300..3919
                     /label=pBR322_origin
                     /ApEinfo_fwdcolor=pink
                     /ApEinfo_revcolor=pink
                     /ApEinfo_graphicformat=arrow_data {{0 1 2 0 0 -1} {} 0}
                     width 5 offset 0
     misc_feature    561..585
                     /label=PP7(3)
                     /ApEinfo_label=PP7
                     /ApEinfo_fwdcolor=cyan
                     /ApEinfo_revcolor=green
                     /ApEinfo_graphicformat=arrow_data {{0 1 2 0 0 -1} {} 0}
                     width 5 offset 0
     misc_feature    488..512
                     /label=PP7(4)
                     /ApEinfo_label=PP7
                     /ApEinfo_fwdcolor=cyan
                     /ApEinfo_revcolor=green
                     /ApEinfo_graphicformat=arrow_data {{0 1 2 0 0 -1} {} 0}
                     width 5 offset 0
     misc_feature    351..375
                     /label=PP7(5)
                     /ApEinfo_label=PP7
                     /ApEinfo_fwdcolor=cyan
                     /ApEinfo_revcolor=green
                     /ApEinfo_graphicformat=arrow_data {{0 1 2 0 0 -1} {} 0}
                     width 5 offset 0
     misc_feature    260..277
                     /label=centromere targeting sequence
                     /ApEinfo_fwdcolor=cyan
                     /ApEinfo_revcolor=green
                     /ApEinfo_graphicformat=arrow_data {{0 1 2 0 0 -1} {} 0}
                     width 5 offset 0
ORIGIN
        1 TAGTTATTAg agggcctatt tcccatgatt ccttcatatt tgcatatacg atacaaggct
       61 gttagagaga taattggaat taatttgact gtaaacacaa agatattagt acaaaatacg
      121 tgacgtagaa agtaataatt tcttgggtag tttgcagttt taaaattatg ttttaaaatg
      181 gactatcata tgcttaccgt aacttgaaag tatttcgatt tcttggcttt atatatcttG
      241 TGGAAAGGAC GAAACACCGA ATCTGCAAGT GGATATTgtt tAagagctaG GAGCAGACGA
      301 TATGGCGTCG CTCCtagcaa gttTaaataa ggctagtccg ttatcaactt CCAGCAGAGC
      361 ATATGGGCTC GCTGGaagtg gcaccgagtc ggtgcTCTGC AGGTCGACTC TAGAGGAGCA
      421 GACGATATGG CGTCGCTCCT GCAGTATTCC CGGGTTCATT AGATCCTAAG GTACCTAATT
      481 GCCTAGACCA GCAGAGCATA TGGGCTCGCT GGCGACACCG AACGTGCGAC AAAGCTAAGT
      541 GCGCCGTACG GAGATCCGAT GGAGCAGACG ATATGGCGTC GCTCCCTTGC GAGATTACGC
      601 GGCACATTAT TGGTCGACGC CGCGAGCTAT CGTCCAGCAG AGCATATGGG CTCGCTGGGG
      661 ACCGAGGGCT CTAGAAATTT TTTTGCGGCC GCGACTCTAG ATCATAATCA GCCATACCAC
```

721 ATTTGTAGAG GTTTTACTTG CTTTAAAAAA CCTCCCACAC CTCCCCCTGA ACCTGAAACA
781 TAAAATGAAT GCAATTGTTG TTGTTAACTT GTTTATTGCA GCTTATAATG GTTACAAATA
841 AAGCAATAGC ATCACAAATT TCACAAATAA AGCATTTTTT TCACTGCATT CTAGTTGTGG
901 TTTGTCCAAA CTCATCAATG TATCTTAAGG CGTAAATTGT AAGCGTTAAT ATTTTGTTAA
961 AATTCGCGTT AAATTTTTGT TAAATCAGCT CATTTTTTAA CCAATAGGCC GAAATCGGCA
1021 AAATCCCTTA TAAATCAAAA GAATAGACCG AGATAGGGTT GAGTGTTGTT CCAGTTTGGA
1081 ACAAGAGTCC ACTATTAAAG AACGTGGACT CCAACGTCAA AGGGCGAAAA ACCGTCTATC
1141 AGGGCGATGG CCCACTACGT GAACCATCAC CCTAATCAAG TTTTTTGGGG TCGAGGTGCC
1201 GTAAAGCACT AAATCGGAAC CCTAAAGGGA GCCCCCGATT TAGAGCTTGA CGGGGAAAGC
1261 CGGCGAACGT GGCGAGAAAG GAAGGGAAGA AAGCGAAAGG AGCGGGCGCT AGGGCGCTGG
1321 CAAGTGTAGC GGTCACGCTG CGCGTAACCA CCACACCCGC CGCGCTTAAT GCGCCGCTAC
1381 AGGGCGCGTC AGGTGGCACT TTTCGGGGAA ATGTGCGCGG AACCCCTATT TGTTTATTTT
1441 TCTAAATACA TTCAAATATG TATCCGCTCA TGAGACAATA ACCCTGATAA ATGCTTCAAT
1501 AATATTGAAA AAGGAAGAGT CCTGAGGCGG AAAGAACCAG CTGTGGAATG TGTGTCAGTT
1561 AGGGTGTGGA AAGTCCCCAG GCTCCCCAGC AGGCAGAAGT ATGCAAAGCA TGCATCTCAA
1621 TTAGTCAGCA ACCAGGTGTG GAAAGTCCCC AGGCTCCCCA GCAGGCAGAA GTATGCAAAG
1681 CATGCATCTC AATTAGTCAG CAACCATAGT CCCGCCCCTA ACTCCGCCCA TCCCGCCCCT
1741 AACTCCGCCC AGTTCCGCCC ATTCTCCGCC CCATGGCTGA CTAATTTTTT TTATTTATGC
1801 AGAGGCCGAG GCCGCCTCGG CCTCTGAGCT ATTCCAGAAG TAGTGAGGAG GCTTTTTTGG
1861 AGGCCTAGGC TTTTGCAAAG ATCGATCAAG AGACAGGATG AGGATCGTTT CGCATGATTG
1921 AACAAGATGG ATTGCACGCA GGTTCTCCGG CCGCTTGGGT GGAGAGGCTA TTCGGCTATG
1981 ACTGGGCACA ACAGACAATC GGCTGCTCTG ATGCCGCCGT GTTCCGGCTG TCAGCGCAGG
2041 GGCGCCCGGT TCTTTTTGTC AAGACCGACC TGTCCGGTGC CCTGAATGAA CTGCAAGACG
2101 AGGCAGCGCG GCTATCGTGG CTGGCCACGA CGGGCGTTCC TTGCGCAGCT GTGCTCGACG
2161 TTGTCACTGA AGCGGGAAGG GACTGGCTGC TATTGGGCGA AGTGCCGGGG CAGGATCTCC
2221 TGTCATCTCA CCTTGCTCCT GCCGAGAAAG TATCCATCAT GGCTGATGCA ATGCGGCGGC
2281 TGCATACGCT TGATCCGGCT ACCTGCCCAT TCGACCACCA AGCGAAACAT CGCATCGAGC
2341 GAGCACGTAC TCGGATGGAA GCCGGTCTTG TCGATCAGGA TGATCTGGAC GAAGAGCATC
2401 AGGGGCTCGC GCCAGCCGAA CTGTTCGCCA GGCTCAAGGC GAGCATGCCC GACGGCGAGG
2461 ATCTCGTCGT GACCCATGGC GATGCCTGCT TGCCGAATAT CATGGTGGAA AATGGCCGCT
2521 TTTCTGGATT CATCGACTGT GGCCGGCTGG GTGTGGCGGA CCGCTATCAG GACATAGCGT
2581 TGGCTACCCG TGATATTGCT GAAGAGCTTG GCGGCGAATG GGCTGACCGC TTCCTCGTGC
2641 TTTACGGTAT CGCCGCTCCC GATTCGCAGC GCATCGCCTT CTATCGCCTT CTTGACGAGT
2701 TCTTCTGAGC GGGACTCTGG GGTTCGAAAT GACCGACCAA GCGACGCCCA ACCTGCCATC
2761 ACGAGATTTC GATTCCACCG CCGCCTTCTA TGAAAGGTTG GGCTTCGGAA TCGTTTTCCG
2821 GGACGCCGGC TGGATGATCC TCCAGCGCGG GGATCTCATG CTGGAGTTCT TCGCCCACCC
2881 TAGGGGGAGG CTAACTGAAA CACGGAAGGA GACAATACCG GAAGGAACCC GCGCTATGAC
2941 GGCAATAAAA AGACAGAATA AAACGCACGG TGTTGGGTCG TTTGTTCATA AACGCGGGGT
3001 TCGGTCCCAG GGCTGGCACT CTGTCGATAC CCCACCGAGA CCCCATTGGG GCCAATACGC
3061 CCGCGTTTCT TCCTTTTCCC CACCCCACCC CCCAAGTTCG GGTGAAGGCC CAGGGCTCGC
3121 AGCCAACGTC GGGGCGGCAG GCCCTGCCAT AGCCTCAGGT TACTCATATA TACTTTAGAT
3181 TGATTTAAAA CTTCATTTTT AATTTAAAAG GATCTAGGTG AAGATCCTTT TTGATAATCT
3241 CATGACCAAA ATCCCTTAAC GTGAGTTTTC GTTCCACTGA GCGTCAGACC CCGTAGAAAA
3301 GATCAAAGGA TCTTCTTGAG ATCCTTTTTT TCTGCGCGTA ATCTGCTGCT TGCAAACAAA
3361 AAAACCACCG CTACCAGCGG TGGTTTGTTT GCCGGATCAA GAGCTACCAA CTCTTTTTCC
3421 GAAGGTAACT GGCTTCAGCA GAGCGCAGAT ACCAAATACT GTCCTTCTAG TGTAGCCGTA
3481 GTTAGGCCAC CACTTCAAGA ACTCTGTAGC ACCGCCTACA TACCTCGCTC TGCTAATCCT
3541 GTTACCAGTG GCTGCTGCCA GTGGCGATAA GTCGTGTCTT ACCGGGTTGG ACTCAAGACG
3601 ATAGTTACCG GATAAGGCGC AGCGGTCGGG CTGAACGGGG GGTTCGTGCA CACAGCCCAG
3661 CTTGGAGCGA ACGACCTACA CCGAACTGAG ATACCTACAG CGTGAGCTAT GAGAAAGCGC
3721 CACGCTTCCC GAAGGGAGAA AGGCGGACAG GTATCCGGTA AGCGGCAGGG TCGGAACAGG
3781 AGAGCGCACG AGGGAGCTTC CAGGGGGAAA CGCCTGGTAT CTTTATAGTC CTGTCGGGTT
3841 TCGCCACCTC TGACTTGAGC GTCGATTTTT GTGATGCTCG TCAGGGGGGC GGAGCCTATG
3901 GAAAAACGCC AGCAACGCGG CCTTTTTACG GTTCCTGGCC TTTTGCTGGC CTTTTGCTCA
3961 CATGTTCTTT CCTGCGTTAT CCCCTGATTC TGTGGATAAC CGTATTACCG CCATGCAT
//

Sequence of psgRNA2.0-6xMS2-cent in GenBank format

```
LOCUS       psgRNA2_0_6xMS2        4024 bp ds-DNA     circular     10-MAR-2016
DEFINITION  pEYFP-N1
ACCESSION
KEYWORDS
  ORGANISM  other sequences; artificial sequences; vectors.
COMMENT
COMMENT
COMMENT     ApEinfo:methylated:1
FEATURES             Location/Qualifiers
     misc_feature    join(259..259,278..690)
                     /label=sgRNA
                     /ApEinfo_fwdcolor=#ffff80
                     /ApEinfo_revcolor=green
                     /ApEinfo_graphicformat=arrow_data {{0 1 2 0 0 -1} {} 0}
                     width 5 offset 0
     misc_feature    433..454
                     /label=MS2SL
                     /ApEinfo_fwdcolor=#008000
                     /ApEinfo_revcolor=green
                     /ApEinfo_graphicformat=arrow_data {{0 1 2 0 0 -1} {} 0}
                     width 5 offset 0
     misc_feature    503..524
                     /label=MS2SL(1)
                     /ApEinfo_label=MS2SL
                     /ApEinfo_fwdcolor=#008000
                     /ApEinfo_revcolor=green
                     /ApEinfo_graphicformat=arrow_data {{0 1 2 0 0 -1} {} 0}
                     width 5 offset 0
     rep_origin      complement(1072..1378)
                     /label=f1_origin
                     /ApEinfo_fwdcolor=pink
                     /ApEinfo_revcolor=pink
                     /ApEinfo_graphicformat=arrow_data {{0 1 2 0 0 -1} {} 0}
                     width 5 offset 0
     promoter        1457..1485
                     /label=AmpR_promoter
                     /ApEinfo_fwdcolor=pink
                     /ApEinfo_revcolor=pink
                     /ApEinfo_graphicformat=arrow_data {{0 1 2 0 0 -1} {} 0}
                     width 5 offset 0
     misc_feature    643..664
                     /label=MS2SL(2)
                     /ApEinfo_label=MS2SL
                     /ApEinfo_fwdcolor=#008000
                     /ApEinfo_revcolor=green
                     /ApEinfo_graphicformat=arrow_data {{0 1 2 0 0 -1} {} 0}
                     width 5 offset 0
     misc_feature    complement(1557..1772)
                     /label=SV40_enhancer
                     /ApEinfo_fwdcolor=pink
                     /ApEinfo_revcolor=pink
                     /ApEinfo_graphicformat=arrow_data {{0 1 2 0 0 -1} {} 0}
                     width 5 offset 0
     promoter        10..258
                     /dnas_title="Human U6 Promoter"
                     /vntifkey="29"
                     /label=Human U6 Promoter
                     /ApEinfo_fwdcolor=pink
                     /ApEinfo_revcolor=pink
                     /ApEinfo_graphicformat=arrow_data {{0 1 2 0 0 -1} {} 0}
                     width 5 offset 0
     promoter        1569..1837
                     /label=SV40_promoter
                     /ApEinfo_fwdcolor=pink
                     /ApEinfo_revcolor=pink
                     /ApEinfo_graphicformat=arrow_data {{0 1 2 0 0 -1} {} 0}
                     width 5 offset 0
```

```
     rep_origin      1736..1813
                     /label=SV40_origin
                     /ApEinfo_fwdcolor=pink
                     /ApEinfo_revcolor=pink
                     /ApEinfo_graphicformat=arrow_data {{0 1 2 0 0 -1} {} 0}
                     width 5 offset 0
     CDS             1920..2714
                     /gene="NeoR/KanR"
                     /note="ORF frame 1"
                     /translation="MIEQDGLHAGSPAAWVERLFGYDWAQQTIGCSDAAVFRLSAQGR
                     PVLFVKTDLSGALNELQDEAARLSWLATTGVPCAAVLDVVTEAGRDWLLLGEVPGQDL
                     LSSHLAPAEKVSIMADAMRRLHTLDPATCPFDHQAKHRIERARTRMEAGLVDQDDLDE
                     EHQGLAPAELFARLKASMPDGEDLVVTHGDACLPNIMVENGRFSGFIDCGRLGVADRY
                     QDIALATRDIAEELGGEWADRFLVLYGIAAPDSQRIAFYRLLDEFF*"
                     /label=NeoR/KanR
                     /ApEinfo_fwdcolor=pink
                     /ApEinfo_revcolor=pink
                     /ApEinfo_graphicformat=arrow_data {{0 1 2 0 0 -1} {} 0}
                     width 5 offset 0
     terminator      2889..3158
                     /label=TK_PA_terminator
                     /ApEinfo_fwdcolor=pink
                     /ApEinfo_revcolor=pink
                     /ApEinfo_graphicformat=arrow_data {{0 1 2 0 0 -1} {} 0}
                     width 5 offset 0
     rep_origin      3306..3925
                     /label=pBR322_origin
                     /ApEinfo_fwdcolor=pink
                     /ApEinfo_revcolor=pink
                     /ApEinfo_graphicformat=arrow_data {{0 1 2 0 0 -1} {} 0}
                     width 5 offset 0
     misc_feature    573..594
                     /label=MS2SL(3)
                     /ApEinfo_label=MS2SL
                     /ApEinfo_fwdcolor=#008000
                     /ApEinfo_revcolor=green
                     /ApEinfo_graphicformat=arrow_data {{0 1 2 0 0 -1} {} 0}
                     width 5 offset 0
     misc_feature    290..323
                     /label=MS2SL(4)
                     /ApEinfo_label=MS2SL
                     /ApEinfo_fwdcolor=#008000
                     /ApEinfo_revcolor=green
                     /ApEinfo_graphicformat=arrow_data {{0 1 2 0 0 -1} {} 0}
                     width 5 offset 0
     misc_feature    360..393
                     /label=MS2SL(5)
                     /ApEinfo_label=MS2SL
                     /ApEinfo_fwdcolor=#008000
                     /ApEinfo_revcolor=green
                     /ApEinfo_graphicformat=arrow_data {{0 1 2 0 0 -1} {} 0}
                     width 5 offset 0
     misc_feature    260..277
                     /label=centromere targeting sequence
                     /ApEinfo_fwdcolor=cyan
                     /ApEinfo_revcolor=green
                     /ApEinfo_graphicformat=arrow_data {{0 1 2 0 0 -1} {} 0}
                     width 5 offset 0
ORIGIN
        1 TAGTTATTAg agggcctatt tcccatgatt ccttcatatt tgcatatacg atacaaggct
       61 gttagagaga taattggaat taatttgact gtaaacacaa agatattagt acaaaatacg
      121 tgacgtagaa agtaataatt tcttgggtag tttgcagttt taaaattatg ttttaaaatg
      181 gactatcata tgcttaccgt aacttgaaag tatttcgatt tcttggcttt atatatcttG
      241 TGGAAAGGAC GAAACACCGA ATCTGCAAGT GGATATTgtt tAagagctaG GCCAACATGA
      301 GGATCACCCA TGTCTGCAGG GCCtagcaag ttTaaataag gctagtccgt tatcaacttG
      361 GCCAACATGA GGATCACCCA TGTCTGCAGG GCCaagtggc accgagtcgg tgcTCTGCAG
      421 GTCGACTCTA GAAAACATGA GGATCACCCA TGTCTGCAGT ATTCCCGGGT TCATTAGATC
      481 CTAAGGTACC TAATTGCCTA GAAAACATGA GGATCACCCA TGTCCGACAC CGAACGTGCG
      541 ACAAAGCTAA GTGCGCCGTA CGGAGATCCG ATAAACATGA GGATCACCCA TGTCCTTGCG
      601 AGATTACGCG GCACATTATT GGTCGACGCC GCGAGCTATC GTAAACATGA GGATCACCCA
```

661 TGTCGGACCG AGGGCTCTAG AAATTTTTTT GCGGCCGCGA CTCTAGATCA TAATCAGCCA
721 TACCACATTT GTAGAGGTTT TACTTGCTTT AAAAAACCTC CCACACCTCC CCCTGAACCT
781 GAAACATAAA ATGAATGCAA TTGTTGTTGT TAACTTGTTT ATTGCAGCTT ATAATGGTTA
841 CAAATAAAGC AATAGCATCA CAAATTTCAC AAATAAAGCA TTTTTTTCAC TGCATTCTAG
901 TTGTGGTTTG TCCAAACTCA TCAATGTATC TTAAGGCGTA AATTGTAAGC GTTAATATTT
961 TGTTAAAATT CGCGTTAAAT TTTTGTTAAA TCAGCTCATT TTTTAACCAA TAGGCCGAAA
1021 TCGGCAAAAT CCCTTATAAA TCAAAAGAAT AGACCGAGAT AGGGTTGAGT GTTGTTCCAG
1081 TTTGGAACAA GAGTCCACTA TTAAAGAACG TGGACTCCAA CGTCAAAGGG CGAAAAACCG
1141 TCTATCAGGG CGATGGCCCA CTACGTGAAC CATCACCCTA ATCAAGTTTT TTGGGGTCGA
1201 GGTGCCGTAA AGCACTAAAT CGGAACCCTA AAGGGAGCCC CCGATTTAGA GCTTGACGGG
1261 GAAAGCCGGC GAACGTGGCG AGAAAGGAAG GGAAGAAAGC GAAAGGAGCG GGCGCTAGGG
1321 CGCTGGCAAG TGTAGCGGTC ACGCTGCGCG TAACCACCAC ACCCGCCGCG CTTAATGCGC
1381 CGCTACAGGG CGCGTCAGGT GGCACTTTTC GGGGAAATGT GCGCGGAACC CCTATTTGTT
1441 TATTTTTCTA AATACATTCA AATATGTATC CGCTCATGAG ACAATAACCC TGATAAATGC
1501 TTCAATAATA TTGAAAAAGG AAGAGTCCTG AGGCGGAAAG AACCAGCTGT GGAATGTGTG
1561 TCAGTTAGGG TGTGGAAAGT CCCCAGGCTC CCCAGCAGGC AGAAGTATGC AAAGCATGCA
1621 TCTCAATTAG TCAGCAACCA GGTGTGGAAA GTCCCCAGGC TCCCCAGCAG GCAGAAGTAT
1681 GCAAAGCATG CATCTCAATT AGTCAGCAAC CATAGTCCCG CCCCTAACTC CGCCCATCCC
1741 GCCCCTAACT CCGCCCAGTT CCGCCCATTC TCCGCCCCAT GGCTGACTAA TTTTTTTTAT
1801 TTATGCAGAG GCCGAGGCCG CCTCGGCCTC TGAGCTATTC CAGAAGTAGT GAGGAGGCTT
1861 TTTTGGAGGC CTAGGCTTTT GCAAAGATCG ATCAAGAGAC AGGATGAGGA TCGTTTCGCA
1921 TGATTGAACA AGATGGATTG CACGCAGGTT CTCCGGCCGC TTGGGTGGAG AGGCTATTCG
1981 GCTATGACTG GGCACAACAG ACAATCGGCT GCTCTGATGC CGCCGTGTTC CGGCTGTCAG
2041 CGCAGGGGCG CCCGGTTCTT TTTGTCAAGA CCGACCTGTC CGGTGCCCTG AATGAACTGC
2101 AAGACGAGGC AGCGCGGCTA TCGTGGCTGG CCACGACGGG CGTTCCTTGC GCAGCTGTGC
2161 TCGACGTTGT CACTGAAGCG GGAAGGGACT GGCTGCTATT GGGCGAAGTG CCGGGGCAGG
2221 ATCTCCTGTC ATCTCACCTT GCTCCTGCCG AGAAAGTATC CATCATGGCT GATGCAATGC
2281 GGCGGCTGCA TACGCTTGAT CCGGCTACCT GCCCATTCGA CCACCAAGCG AAACATCGCA
2341 TCGAGCGAGC ACGTACTCGG ATGGAAGCCG GTCTTGTCGA TCAGGATGAT CTGGACGAAG
2401 AGCATCAGGG GCTCGCGCCA GCCGAACTGT TCGCCAGGCT CAAGGCGAGC ATGCCCGACG
2461 GCGAGGATCT CGTCGTGACC CATGGCGATG CCTGCTTGCC GAATATCATG GTGGAAAATG
2521 GCCGCTTTTC TGGATTCATC GACTGTGGCC GGCTGGGTGT GGCGGACCGC TATCAGGACA
2581 TAGCGTTGGC TACCCGTGAT ATTGCTGAAG AGCTTGGCGG CGAATGGGCT GACCGCTTCC
2641 TCGTGCTTTA CGGTATCGCC GCTCCCGATT CGCAGCGCAT CGCCTTCTAT CGCCTTCTTG
2701 ACGAGTTCTT CTGAGCGGGA CTCTGGGGTT CGAAATGACC GACCAAGCGA CGCCCAACCT
2761 GCCATCACGA GATTTCGATT CCACCGCCGC CTTCTATGAA AGGTTGGGCT TCGGAATCGT
2821 TTTCCGGGAC GCCGGCTGGA TGATCCTCCA GCGCGGGGAT CTCATGCTGG AGTTCTTCGC
2881 CCACCCTAGG GGGAGGCTAA CTGAAACACG GAAGGAGACA ATACCGGAAG GAACCCGCGC
2941 TATGACGGCA ATAAAAAGAC AGAATAAAAC GCACGGTGTT GGGTCGTTTG TTCATAAACG
3001 CGGGGTTCGG TCCCAGGGCT GGCACTCTGT CGATACCCCA CCGAGACCCC ATTGGGGCCA
3061 ATACGCCCGC GTTTCTTCCT TTTCCCCACC CCACCCCCCA AGTTCGGGTG AAGGCCCAGG
3121 GCTCGCAGCC AACGTCGGGG CGGCAGGCCC TGCCATAGCC TCAGGTTACT CATATATACT
3181 TTAGATTGAT TTAAAACTTC ATTTTTAATT TAAAAGGATC TAGGTGAAGA TCCTTTTTGA
3241 TAATCTCATG ACCAAAATCC CTTAACGTGA GTTTTCGTTC CACTGAGCGT CAGACCCCGT
3301 AGAAAAGATC AAAGGATCTT CTTGAGATCC TTTTTTTCTG CGCGTAATCT GCTGCTTGCA
3361 AACAAAAAAA CCACCGCTAC CAGCGGTGGT TTGTTTGCCG GATCAAGAGC TACCAACTCT
3421 TTTTCCGAAG GTAACTGGCT TCAGCAGAGC GCAGATACCA AATACTGTCC TTCTAGTGTA
3481 GCCGTAGTTA GGCCACCACT TCAAGAACTC TGTAGCACCG CCTACATACC TCGCTCTGCT
3541 AATCCTGTTA CCAGTGGCTG CTGCCAGTGG CGATAAGTCG TGTCTTACCG GGTTGGACTC
3601 AAGACGATAG TTACCGGATA AGGCGCAGCG GTCGGGCTGA ACGGGGGGTT CGTGCACACA
3661 GCCCAGCTTG GAGCGAACGA CCTACACCGA ACTGAGATAC CTACAGCGTG AGCTATGAGA
3721 AAGCGCCACG CTTCCCGAAG GGAGAAAGGC GGACAGGTAT CCGGTAAGCG GCAGGGTCGG
3781 AACAGGAGAG CGCACGAGGG AGCTTCCAGG GGGAAACGCC TGGTATCTTT ATAGTCCTGT
3841 CGGGTTTCGC CACCTCTGAC TTGAGCGTCG ATTTTTGTGA TGCTCGTCAG GGGGGCGGAG
3901 CCTATGGAAA AACGCCAGCA ACGCGGCCTT TTTACGGTTC CTGGCCTTTT GCTGGCCTTT
3961 TGCTCACATG TTCTTTCCTG CGTTATCCCC TGATTCTGTG GATAACCGTA TTACCGCCAT
4021 GCAT
//
